# Heavy Alkaline Earth Cyclic (Alkyl)(Amino)Carbene Complexes Supported by Aryl–Silyl Amides

**DOI:** 10.1021/acs.inorgchem.4c03494

**Published:** 2024-11-06

**Authors:** Alex W. J. Bowles, Yu Liu, Matthew P. Stevens, Fabrizio Ortu

**Affiliations:** † School of Chemistry, University of Leicester, University Road, Leicester LE1 7RH, U.K.; ‡ Department of Chemistry, University of Bath, Claverton Down, Bath BA2 7AY, U.K.

## Abstract

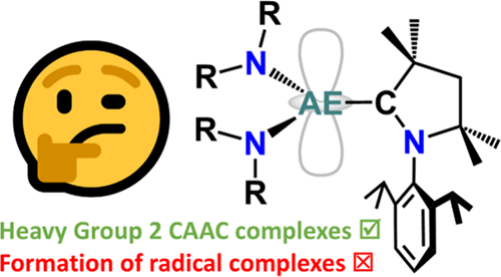

A series of 8 trigonal planar, heavy alkaline earth (AE = Ca–Ba) metal complexes containing cyclic (alkyl)(amino)carbene (CAAC) ligands were prepared from AE bis(amide) species. Complexation can be achieved by first generating the free carbene *in situ* or by direct addition of the free carbene, with the former route giving rise to unexpected mixed-amide AE complexes. The frontier molecular orbitals of the highly equatorial, 3-coordinate AE-CAAC species were also probed computationally, revealing the lowest unoccupied molecular orbital (LUMO) consisting predominantly of the π* system located on the carbene ligand.

## Abstract 2

A series of heavy Group 2 complexes featuring cyclic (alkyl)(amino)carbene ligands have been synthesized and characterized using spectroscopic techniques and single-crystal X-ray diffraction. Reductions of such complexes do not yield low-oxidation state compounds, and DFT analysis shows some metal-based *d*-character in the frontier orbitals.

## 1 Introduction

**Figure 1:**
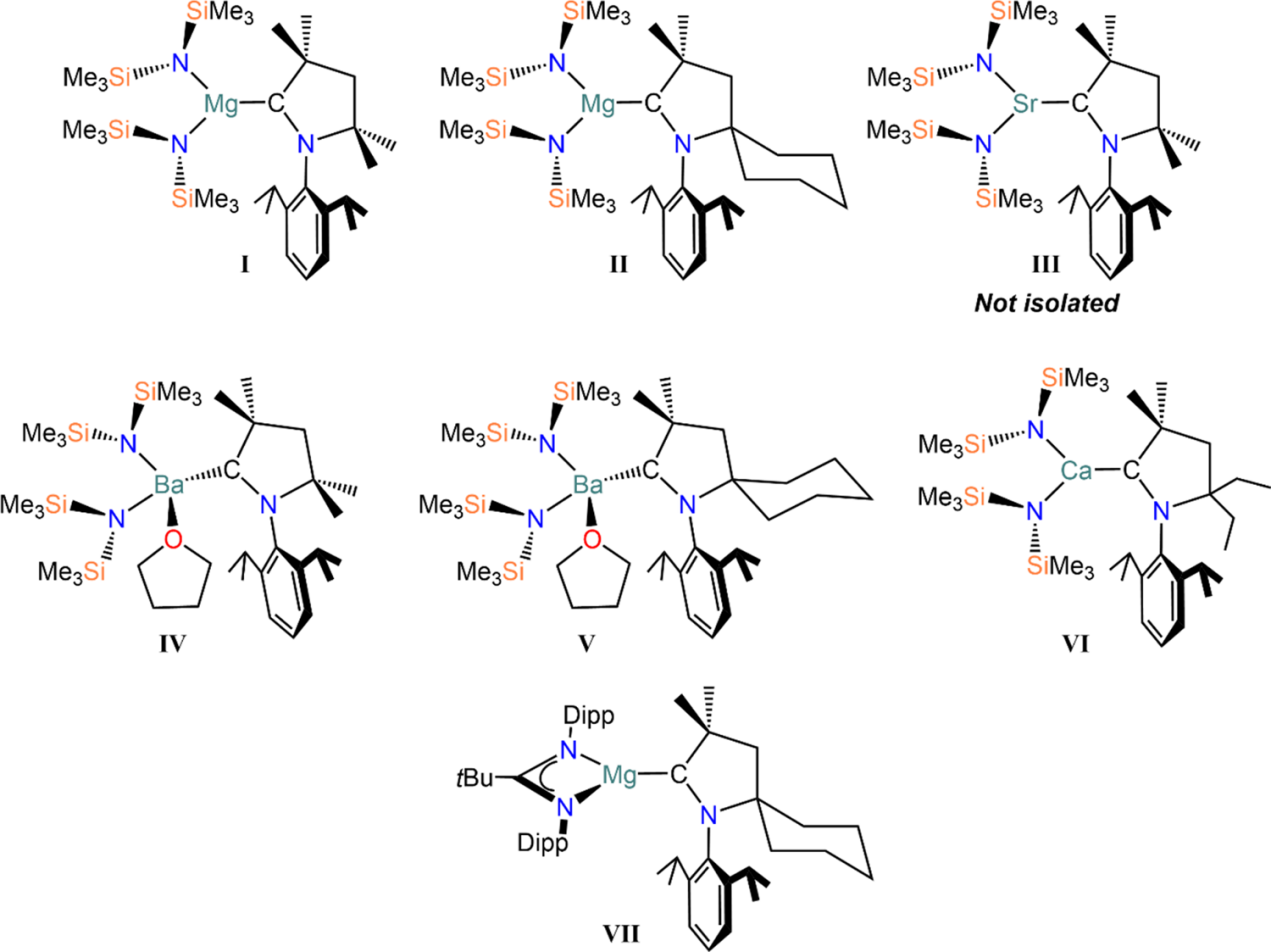
Examples of amide-stabilized AE-CAAC complexes reported by Turner (**I–V**),^9^ Gilliard (**VI**),^4^ and Harder (**VII**).^10^

Carbene ligands have been used extensively in Group 2 chemistry. In particular, *N*-heterocyclic carbenes (NHCs)^1^ have been used to stabilize low-coordinate AE complexes supported by alkyl,^2^ amide,^3,4^ cyclopentadienyl,^5^ and aryloxide ligands.^6^ In recent years, cyclic (alkyl)(amino)carbenes ({:CC(R)_2_CH_2_C(Me)_2_N(Dipp)} – CAAC^R^; R = methyl, ethyl, cyclohexyl, Dipp = 2,6-diisopropylphenyl) have emerged as a very important class of carbene donors.^7,8^ Several AE complexes containing CAAC ligands have previously been reported by Turner (**I–V**)^9^ and Gilliard (**VI**),^4^ featuring the ubiquitous hexamethyldisilylamide ligand, {N(SiMe_3_)_2_}^−^ (N″). Braunschweig and Gillard have extended the use of CAAC ligands to Be, and reported a series of complexes ([Be(CAAC)_2_], [Be(CAAC)(CAAC^H^)] (CAAC^H^ = {CHC(Et)_2_CH_2_C(Me)_2_N(Dipp)}^−^) and [Be(CAAC)_2_]^−^)^10−13^ where the carbene ligands are formally reduced to the CAAC^•–^ radical anion.^14^ Turner attempted the isolation of an analogous Mg complex, [Mg(CAAC)_2_], which resulted in decomposition of the carbene ligand.^15^ Furthermore, Harder and co-workers reported the isolation of a monomeric magnesium radical [Mg{*^t^*BuC(N-Dipp)_2_}(CAAC^Cy^)] (**VII**, Figure 1) via mechanochemical reduction.^10,16^ Similarly to previous examples of CAAC-containing Be radicals, **VII** is better described as a [Mg{*^t^*BuC(N-Dipp)_2_}]^+^ cation and a CAAC^•–^ radical anion due to the considerable spin density situated on the carbene ligand.^14^

**Scheme 1:**
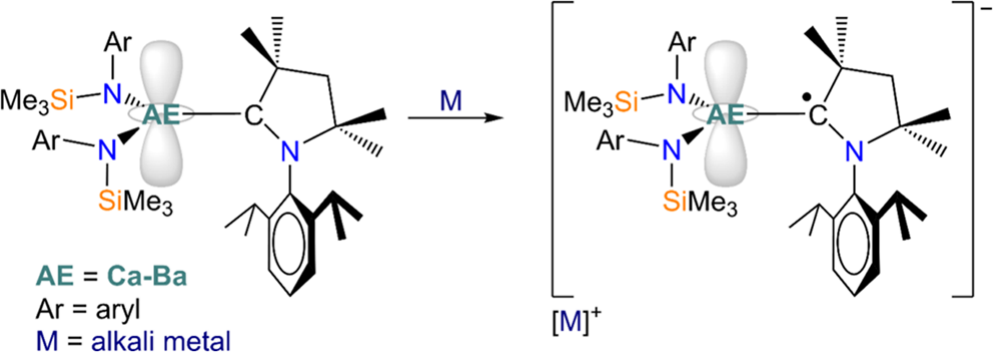
Proposed Reduction of AE(II) Precursors Complexes [AE(L)_2_(CAAC)] in Pursuit of Carbene AE(II) Radical Complexes

The use of CAAC ligands in low-oxidation state AE chemistry is particularly interesting, owing to the great advances in the stabilization of AE(I) and AE(0) complexes over the last three decades.^17−24^ In particular, Mg(I) dimers have been established as bespoke reducing agents since their initial isolation in 2007,^25^ and have demonstrated several advantages over traditional alkali metal reducing agents (e.g., high solubility, more precise reductive chemistry) which have led to remarkable applications in small molecule activation^24,26^ and low-oxidation state main group chemistry.^23,27^ However, despite the increasing popularity and useful applications of dimeric Mg(I) complexes, analogous chemistry with the heavier Group 2 elements (Ca–Ba) is hugely underdeveloped; to date, the inverse sandwich complex [(THF)_3_Ca{μ-C_6_H_3_-1,3,5-Ph_3_}Ca(THF)_3_] reported by Westerhausen and co-workers is the only example of a structurally authenticated heavy AE(I) complex,^28^ though the oxidation state assignment of the calcium centers has been disputed.^29^ Additionally, attempts to cleave the metal–metal bond in Mg(I) dimers and isolate a formally monomeric Mg(I) species have so far been unsuccessful.^10,16,21,30^ Recently, we have reported that complexes of the heavier AE elements (Ca–Ba) with highly equatorial ligand environments display a lowest unoccupied molecular orbital (LUMO) with significant *d-*character, and hypothesized that accessible *d*-orbitals could be exploited for the isolation of a heavy AE(I) species.^31^ Our theoretical calculations showed that the reduction of anionic complexes [AE(L)_3_]^−^ (L = monodentate amide) to putative AE(I) dianions [AE(L)_3_]^2–^ is exergonic; nonetheless, attempts to synthesize and isolate these species were unsuccessful. We reasoned that one issue associated with these reductions could arise from the high negative charge of the target dianions [AE(L)_3_]^2–^, thus making neutral precursors more promising candidates for reductive chemistry. In light of this, we updated these design criteria by combining a neutral donor with two sterically demanding, monodentate amide ligands—N″, {N(Mes)(SiMe_3_)}^−^ (Mes = 2,4,6-trimethylphenyl), {N(Dipp)(SiMe_3_)}^−^—in order to achieve the desired trigonal planar geometry and overall neutral charge (Scheme 1). In this work, we aim to further probe the stabilization of the *d*-orbital manifold in heavy AE metals, and the potential effect on the stabilization of carbene radical complexes.

## 2 Results and Discussion

### 2.1 Synthesis and Characterization of Bis(amide) Precursors **1-AE** and **2-AE**

**Scheme 2:**
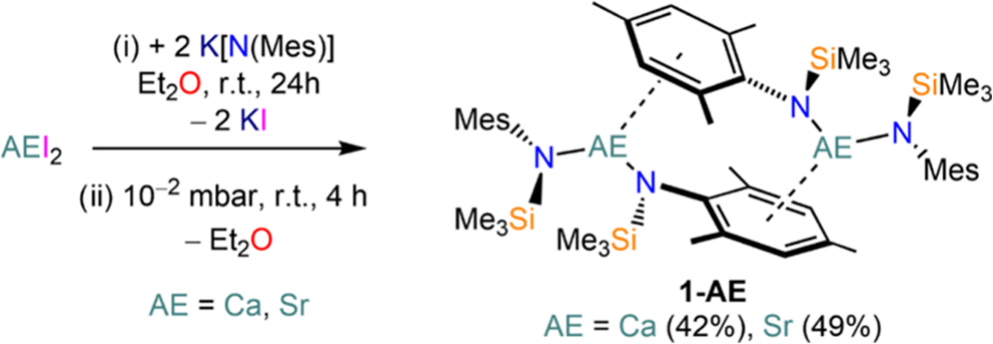
Synthesis of Lewis Base-Free Bis(amide) Complexes **1-Ca** and **1-Sr**

The AE bis(amide) starting materials [AE{N(Mes)(SiMe_3_)}_2_(THF)_2_] (**1-AE·(THF)**_**2**_; AE = Ca–Ba) and [AE{N(Dipp)(SiMe_3_)}_2_(THF)_2_] (**2-AE·(THF)**_**2**_; AE = Ca–Ba) were prepared according to modified literature procedures.^32,33^ A polymorph of **2-AE·(THF)**_**2**_ was also obtained (see Supporting information (SI)).^32^ We also prepared the Lewis base-free bis(amide) complexes [AE{N(Mes)(SiMe_3_)}_2_] (**1-AE**; AE = Ca, Sr) to investigate their suitability as precursors to three-coordinate AE–CAAC complexes. Addition of two equivalents of K[N(Mes)(SiMe_3_)] to the respective AEI_2_ salt in diethyl ether yielded the solvated bis(amide) [AE{N(Mes)(SiMe_3_)}_2_(Et_2_O)] (Scheme 2) which can be easily desolvated *in vacuo*, as confirmed by ^1^H NMR spectroscopy. In some instances, the tris(amide) calciate or strontiate complexes [AE{N(Mes)(SiMe_3_)}_3_K] were identified in the product mixture,^31^ especially in the case of strontium and when working on larger scales (>1 mmol of AEI_2_). To counter this, we found that a slight excess of the AEI_2_ (5%) and longer reaction times of up to 48 h resulted in the clean formation of **1-AE** as the thermodynamic product. The solubility of **1-Ca** and **1-Sr** is greatly reduced compared with their solvated counterparts, **1-AE·(THF)**_**2**_. The ^1^H NMR spectrum of **1-Ca** suggests inequivalence of the two {N(Mes)(SiMe_3_)}^−^ ligands with two sets of signals for each proton environment with equal integrations. An analogous ^1^H NMR spectrum is obtained for **1-Sr**, however, the resonances are much broader owing to its poor solubility in C_6_D_6_. The insolubility of **1-Sr** in C_6_D_6_ prevented the collection of interpretable ^13^C{^1^H} and ^29^Si{^1^H} NMR spectra. Addition of a few drops of THF to samples of **1-Ca** and **1-Sr** fully dissolves the material and gives rise to the highly symmetric NMR spectra of the respective, known monomeric bis(amide) solvates, **1-AE·(THF)**_**2**_.^33^

**Figure 2:**
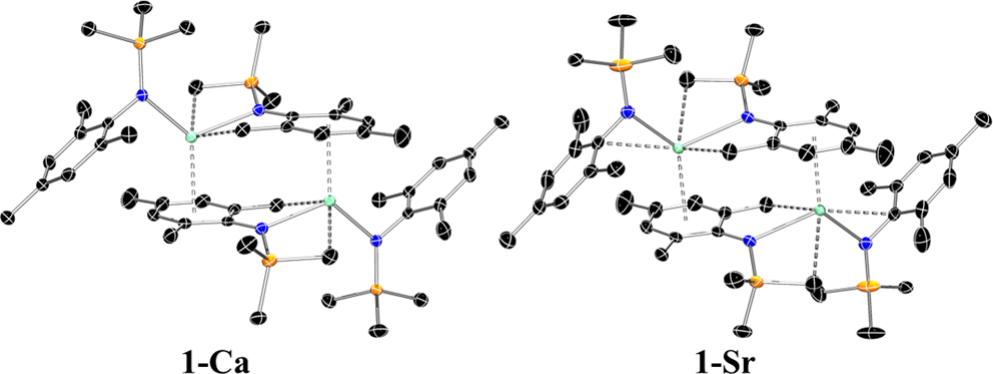
Crystal structures of **1-Ca** (left) and **1-Sr** (right). Ellipsoids are set at the 50% probability level. Hydrogen atoms have been omitted for clarity. The asymmetric unit contains only one-half of the molecule, the full molecule has been reproduced in this figure using symmetry operations to generate equivalent atoms: *i =* −*x*, −*y*, −*z*. Legend: carbon (black), nitrogen (blue), silicon (orange), AE metal (aquamarine).

Crystals of **1-Ca** and **1-Sr** suitable for single-crystal X-ray diffraction (XRD) studies were obtained from saturated hexane solutions (see Experimental Section for full details). They both crystallize in the triclinic *P*1̅ space group with only half of the dimer present in the asymmetric unit and are essentially isostructural (Figure 2). In the absence of coordinating solvents, the electropositive AE^2+^ centers interact with a mesityl ring from a neighboring unit in an η^6^-coordination mode [AE···Centroid_mesityl_: 2.5399(8) Å **1-Ca**; 2.6849(10) Å **1-Sr**] giving rise to dimeric units. This bridging mode is in contrast to AE bis(amide) complexes that do not possess an aryl substituent, where usually the *N*-donor atoms of the ligands bridge between metal centers.^34,35^ Interactions with aryl moieties are not uncommon for the Group 2 metals and have been shown to stabilize AE complexes with low coordination numbers, together with preventing Schlenk redistribution products.^36−38^ Although complexes of the heavier AE elements featuring (aryl)(silyl)amide ligands are known,^32,33,39^ to the best of our knowledge complexes **1-Ca** and **1-Sr** are the first structurally authenticated, solvent-free examples of this kind. The AE–N bond distances [Ca–N 2.287(1) and 2.3830(9) Å; Sr–N 2.422(2) and 2.538(1) Å] are within the expected range for each metal.^33−35^ In both cases, the AE–N distance of the terminal amide is marginally shorter than that of the bridging amide. The N–AE–N bond angles in **1-Ca** [118.64(4)°] and **1-Sr** [115.39(6)°] vary due to the differing ionic radii of the metal cations.

In the absence of coordinating solvent molecules, the dimeric structures of **1-Ca** and **1-Sr** are retained in solution, thereby explaining their asymmetric ^1^H NMR spectra. The unsolvated forms are noticeably more sensitive to air and moisture than their corresponding THF adducts owing to the more exposed AE^2+^ metal center. Repeated attempts to prepare the lighter congener **1-Mg** via an alkane elimination reaction between ^n^Bu_2_Mg and HN(Mes)(SiMe_3_) yielded only the bis(THF) adduct [AE{N(Mes)(SiMe_3_)}_2_(THF)_2_],^33^ even when using newly opened ^n^Bu_2_Mg in heptane and freshly distilled amine. We attribute this to residual THF in the dibutyl magnesium reagent carried over from its production process.^40^ Attempts to desolvate [AE{N(Mes)(SiMe_3_)}_2_(THF)_2_] *in vacuo* with heating (<10^–2^ mbar, 100 °C) were partially successful yielding the monosolvated complex [AE{N(Mes)(SiMe_3_)}_2_(THF)].

### 2.2 Synthesis and Characterization of Mixed-Amide CAAC Complexes **3-AE** and **4-AE**

**Scheme 3:**
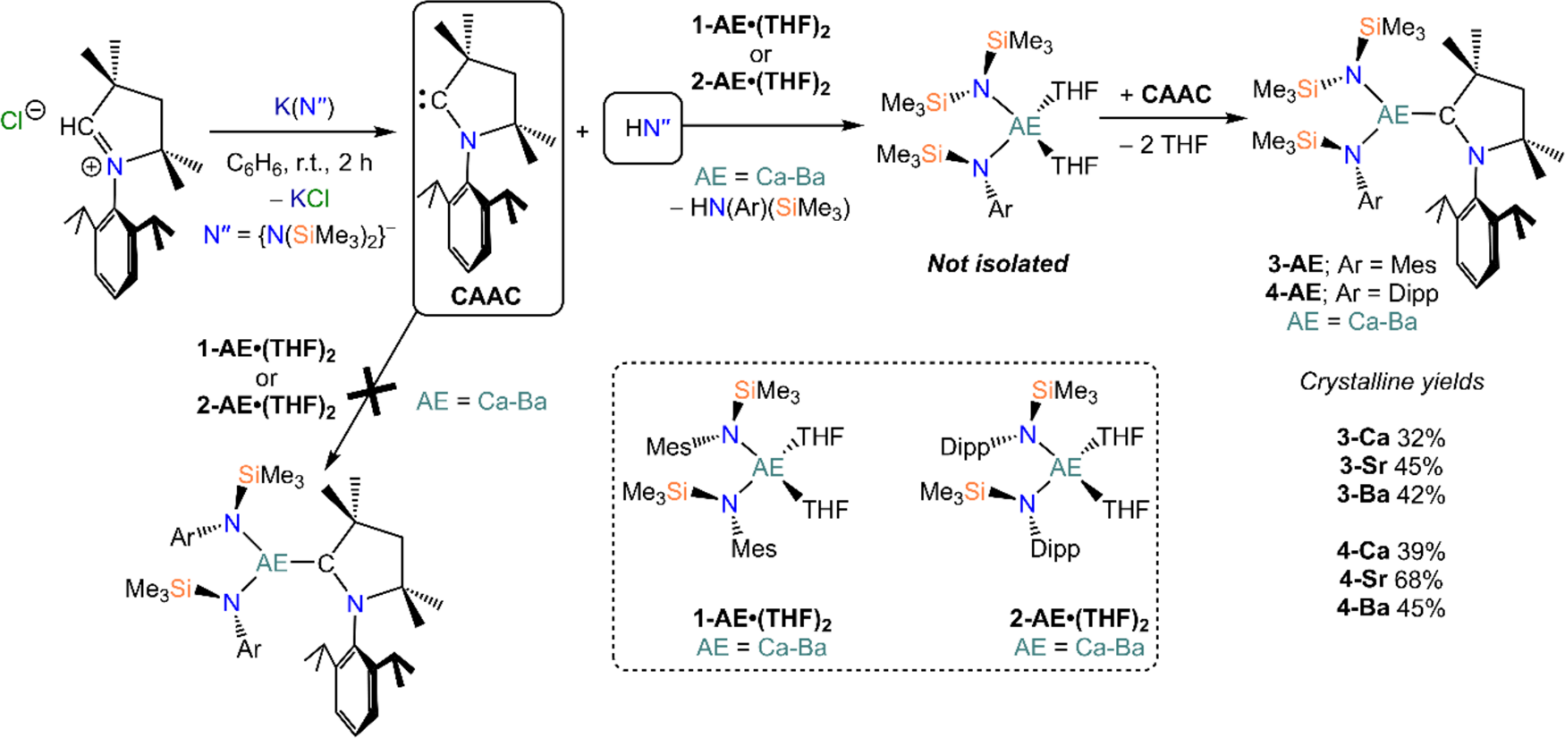
Synthetic Route to Complexes **3-AE** and **4-AE** AE = Ca–Ba; Mes = 2,4,6-trimethylphenyl; Dipp = 2,6-diisopropylphenyl.

The free-carbene CAAC was generated *in situ* from the corresponding iminium salt by addition of K(N″) before being added to the respective AE bis(amide) starting material and stirred for 2 h at room temperature, akin to previous studies (Scheme 3).^9,41^ Upon removal of the volatile components, oily residues were obtained in all cases even after extended drying under reduced pressure (<10^–2^ mbar, 55 °C). Colorless crystals were obtained from extracting the crude reaction material with portions of hexane or pentane (see Experimental Section for recrystallization details). Single-crystal XRD studies revealed the formation of the mixed-amide CAAC complexes [AE{N(Mes)(SiMe_3_)}(N″)(CAAC)] (**3-AE**; AE = Ca–Ba) or [AE{N(Dipp)(SiMe_3_)}(N″)(CAAC)] (**4-AE**; AE = Ca–Ba).

**Scheme 4:**
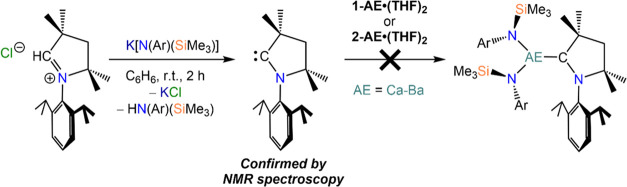
Attempted Synthesis of [AE{N(Ar)(SiMe_3_)}_2_(CAAC)] Complexes (AE = Ca–Ba; Ar = Mes, Dipp)

We postulate that the presence of the HN″ amine (byproduct from the generation *in situ* of the free carbene) prompts a protonolysis reaction, with the less sterically demanding mixed-amide complex being thermodynamically favored. To probe this further, the corresponding potassium (aryl)amide—K[N(Mes)(SiMe_3_)] for **1-AE·(THF)**_**2**_, K[N(Dipp)(SiMe_3_)] for **2-AE·(THF)**_**2**_—was used as the deprotonating base in place of K(N″) to generate the free carbene *in situ* (Scheme 4). The free carbene was then added to either **1-AE·(THF)**_**2**_ or **2-AE·(THF)**_**2**_, however no complexation of CAAC to the Group 2 metal was observed in either case. Control reactions were performed to ensure that both K[N(Mes)(SiMe_3_)] and K[N(Dipp)(SiMe_3_)] were successful in generating the free carbene from the reaction with the iminium salt precursor, as was confirmed by the characteristic downfield resonance of the free carbene at 313 ppm in the ^13^C{^1^H} NMR spectra and the presence of the corresponding aryl(silyl)amine, HN(Mes)(SiMe_3_) or HN(Dipp)(SiMe_3_), in the ^1^H NMR spectra. Furthermore, addition of isolated free CAAC to **1-AE·(THF)**_**2**_ or **2-AE·(THF)**_**2**_ does not yield the corresponding AE-CAAC complexes, even when heating the reaction mixture for extended periods of time, suggesting that complexation of the bulky carbene ligand can only occur when the steric demands around the AE center are reduced.

**Figure 3:**
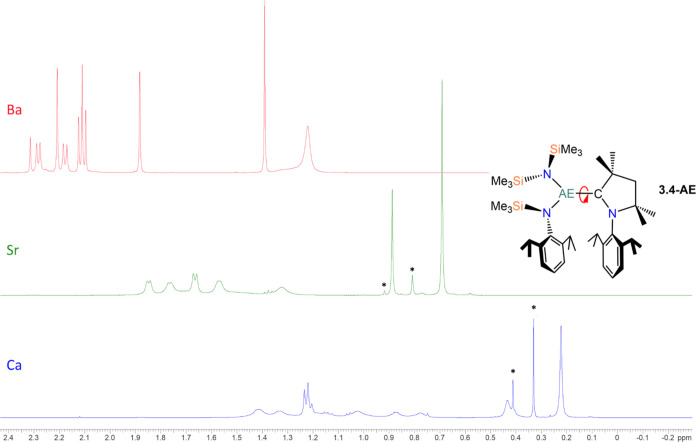
Overlayed ^1^H NMR spectra of **4-AE** (region −0.2 to 2.4 ppm), offset for clarity; Ca (blue), Sr (green; +0.5 ppm), Ba (red; +1.0 ppm). *Signals assigned to mixed-amide complex **5** and its Sr analogue (not isolated).

**Scheme 5:**
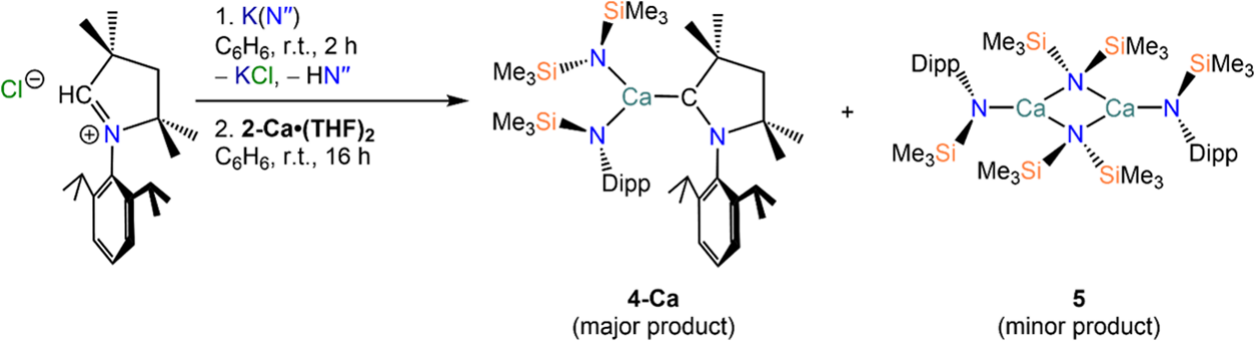
Synthesis of **4-Ca** and Concomitant Formation of **5**

The yields of **3-AE** and **4-AE** were low to moderate, and did not improve when a two-fold excess of CAAC was employed. We ascribe the relatively low yields to the presence of competing reactions and formation of various byproducts, which are discussed herein. Recrystallization of complex **4-Ca** yielded crystals of the carbene-free complex [{Ca(NDipp})(μ-N″)}_2_] (**5**, Scheme 5), the structure of which was determined by XRD studies (see SI). These observations suggest that the complexation of CAAC to calcium is in competition with the formation of **5**, which is likely formed as a result of protonolysis of [Ca{N(Mes)(SiMe_3_)}_2_(THF)_2_] (**2-Ca·(THF)**_**2**_) with HN″. Resonances corresponding to the two trimethylsilyl environments of complex **5** can be seen in the ^1^H NMR spectrum of **4-Ca** (Figure 3); analogous resonances are also observed in the ^1^H NMR spectrum of **4-Sr**, albeit present in smaller quantities, and are assumed to be the strontium congener of **5**, though this compound was never isolated.

**Scheme 6:**
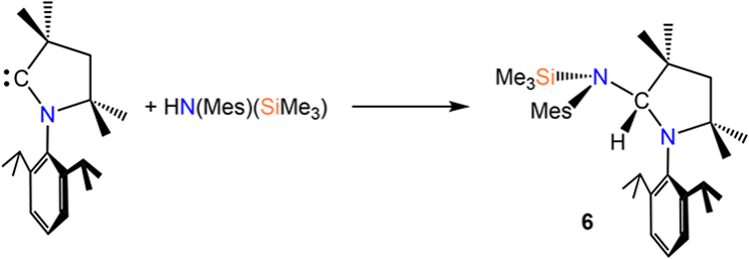
Reactivity of CAAC with HN(Mes)(SiMe_3_) and Formation of **6**

Furthermore, during the isolation of complex **3-Ca**, crystalline crops of a tertiary amine byproduct [N(Mes)(SiMe_3_)(^H^CAAC)] (**6**, Scheme 6), **1-Ca·(THF)**_**2**_ and the unsolvated congener **1-Ca** were all isolated prior to **3-Ca**. In several cases, during the synthesis of **3**, the amine byproducts **6** cocrystallized with the respective AE-CAAC complex and could not be separated; we hypothesize that a similar side-reaction could be taking place in the case of **4-AE**, leading to the formation of [N(Dipp)(SiMe_3_)(^H^CAAC)], though this has not been isolated. Activation of unactivated N–H bonds is known for CAACs.^42^ The formation of **6** is further indicative of HN(Mes)(SiMe_3_) being eliminated as a byproduct of the protonolysis reaction between **1-AE·(THF)**_**2**_ and HN″, the latter of which is formed as a byproduct of the initial deprotonation of the iminium salt precursor (*vide supra*Scheme 3).^42^

Complexes **3-AE** and **4-AE** (AE = Ca–Ba) were characterized by multinuclear NMR spectroscopy, Fourier transform infrared (FT-IR) spectroscopy, XRD studies and, in most cases, CHN elemental analysis. In the ^13^C NMR spectra of these compounds, the chemical shift of the carbenoid resonance is highly diagnostic and can be indicative of complexation to the metal center; the free carbene signal is characteristically located downfield at 313 ppm, and is upfield-shifted upon complexation to the AE metal center (δ_C_/ppm = **3-Ca**: 276.5; **4-Ca**: 276.4; **3-Sr**: 283.2; **4-Sr**: 282.6; **3-Ba**: 294.8; **4-Ba**: 298.6). The trend between the ^13^C chemical shift of the carbene atom and the size of the AE^2+^ cation is a consequence of the increasing ionic character of the AE–C_carbene_ bond with increasing size of the ionic radii from Ca^2+^ < Sr^2+^ < Ba^2+^. Though these signals offer a useful spectroscopic handle which can be used to monitor the complexation reaction, by their nature they exhibit only very weak signals in the ^13^C{^1^H} NMR spectra and, in some cases, were only observable by two-dimensional ^1^H–^13^C HMBC experiments. The signals in the ^1^H NMR spectra of **4-Ca** are notably broad and poorly resolved. We attribute this to the lack of free-rotation of the carbene ligand about the Ca–C_carbene_ bond, demonstrated by the slight broadening of the signals in the case of the strontium congener **4-Sr** and well-resolved signals in the case of **4-Ba**, wherein the CAAC ligand can rotate freely about the longer Ba–C_carbene_ bond (Figure 3). A similar trend is observed for the less sterically demanding, mesityl-functionalized congeners **3-AE**, albeit to a lesser extent.

**Figure 4:**
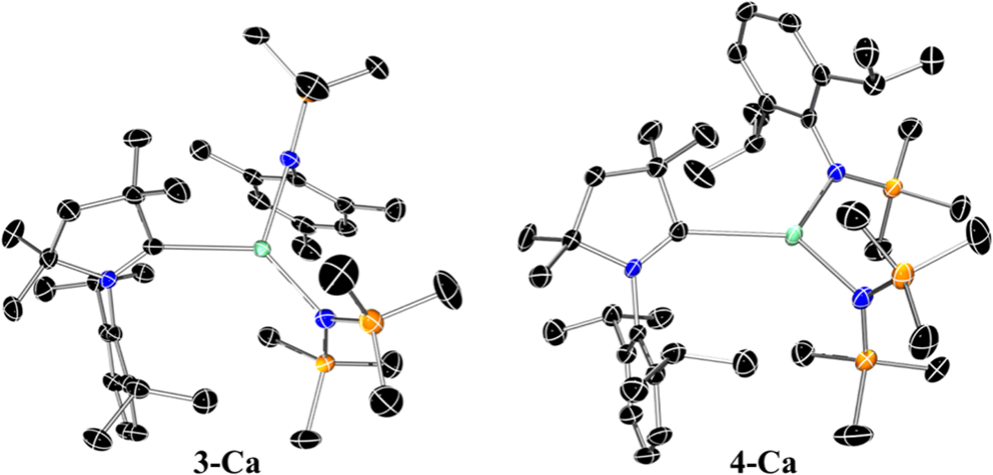
Crystal structure of mixed-amide CAAC complexes **3-Ca** (left) and **4-Ca** (right). Ellipsoids are set at the 50% probability level. Hydrogen atoms have been omitted for clarity; in the case of **3-Ca** two molecules are present in the asymmetric unit and only one is shown for clarity. Legend: carbon (black), nitrogen (blue), silicon (orange), calcium (aquamarine).

Complex **3-Ca** crystallizes in the triclinic *P*1̅ space group with two molecules contained within the asymmetric unit (Figure 4). The closely related complex **4-Ca** crystallized in the monoclinic *P*2_1_/*n* space group with the asymmetric unit containing one molecule (Figure 4). In both cases, the geometry about the Ca centers is a distorted trigonal planar, with bond angles in the range of 110.57(4)–122.78(4)° (**3-Ca**) and 113.1(2)–126.0(2)° (**4-Ca**) and a slight deviation of the Ca atom from the plane formed by the ligands [Ca···N^∧^N^∧^C_plane_: 0.3277(7) and 0.3256(7) Å (**3-Ca**); 0.117(4) Å (**4-Ca**)]. The conformation of the carbene ligand is notably different in **3-Ca** and **4-Ca**, as exemplified by the respective N_Ar_–Ca–C–N_CAAC_ torsion angles of −135.5(1)° and 137.5(1) (**3-Ca**), and 138.9(7)° (**4-Ca**). The conformational differences are likely caused by the steric interactions of the aryl functionalities of the aryl(silyl) amide ligand and the Dipp group of the carbene ligand. In **3-Ca**, the mesityl ring is on the same side as the Dipp group, whereas in **4-Ca** both aryl functionalities are on opposing sides. Due to the paucity of structurally authenticated three-coordinate AE-CAAC complexes of the heavier Group 2 metals, meaningful comparisons can only be made with the calcium analogue previously reported by Gilliard and co-workers [Ca(N″)_2_(^Et^CAAC)] (**VI**), which features the slightly bulkier diethyl carbene ligand, CAAC^Et^.^4^ The Ca–C_CAAC_ distances of **4-Ca** [2.677(7) Å] and **VI** [2.700(5) Å] are comparable, while the same bond interactions are shorter in **3-Ca** [2.6368(13) and 2.6593(13) Å].

**Figure 5:**
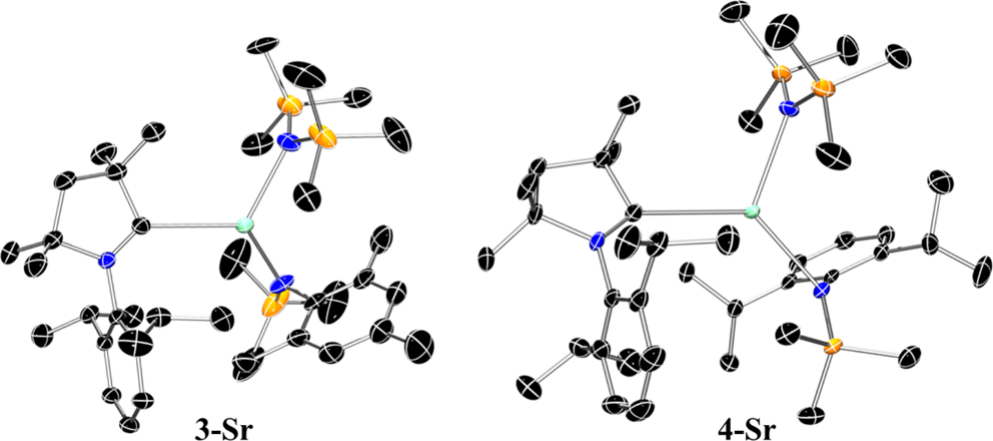
Crystal structure of **3-Sr** (left) and **4-Sr** (right). Ellipsoids are set at 50% probability level. Hydrogen atoms have been omitted for clarity. Legend: carbon (black), nitrogen (blue), silicon (orange), strontium (aquamarine).

Complexes **3-Sr** and **4-Sr** crystallize in the monoclinic space groups *C*2/*c* and *P*2_1_/*n* respectively (Figure 5). As with the lighter congeners **3-Ca** and **4-Ca**, the geometry around the Group 2 center is distorted trigonal planar, with bond angles ranging between 113.43(7)–127.4(1)° (**3-Sr**) and 110.78(4)–128.30(4)° (**4-Sr**). In both cases, the coordination environment of the ligands is highly equatorial with negligible distortion of the planar geometry [Sr···N^∧^N^∧^C_plane_: 0.253(2)–0.276(2) Å (**3-Sr**); 0.0231(6) Å (**4-Sr**)]. The Sr–C_CAAC_ bond lengths [**3-Sr:** 2.861(6) Å; **4-Sr:** 2.8312(12) Å] fall satisfyingly within the expected trend as the ionic radii increases descending the group from Ca to Ba. This trend is corroborated by observing the characteristic resonances of the carbene carbon in the ^13^C NMR spectra, which are increasingly shifted downfield as the size of the AE^2+^ cation increases and the AE–C bond becomes more ionic in character (Table 1). The only notable difference between **3-Sr** and **4-Sr** is the relative orientation of the CAAC ligand with respect to the aryl(silyl) amide, with the conformation of the carbene ligand analogous to that of the respective calcium congeners. In **3-Sr**, the N_Ar_–Sr–C–N_CAAC_ torsion angle ranges between 35.7(7)–43.5(7)°, with the aryl functionalities of both the {N(Mes)(SiMe_3_)}^−^ ligand and the carbene positioned on the same face of the complex. This is in contrast with the conformation exhibited by **4-Sr**, where the N_Ar_–Sr–C–N_CAAC_ torsion angle is −136.41(10)°, and the aryl rings of the {N(Dipp)(SiMe_3_)}^−^ ligand and CAAC are positioned on opposing sides. Notably, complexes **3-Sr** and **4-Sr** are the first crystallographically authenticated Sr-CAAC complexes.

**Table 1:** Selected Structural and Spectroscopic Parameters of Complexes **3-AE**, **4-AE**, **7**, and **8**

	AE–C_carbene_ (Å)	AE–N_1_ (Å)	AE–N_2_ (Å)	AE–N∧N∧C	^13^C{^1^H} δC_carbene_ (ppm)
**3-Ca**	2.6368(13) 2.6593(13)	2.2804(11) 2.2750(11)	2.3061(11)	0.3277(7) 0.3256(7)	276.49^a^
			2.2882(11)		
**3-Sr**	2.861(6)	2.433(2)	2.438(2)	0.253(2)	283.21^b^^,^^c^
**3-Ba**	3.069(3)	2.580(2)	2.597(2)	0.3752(14)	294.80^b^^,^^c^
**4-Ca**	2.677(7)	2.279(6)	2.306(6)	0.117(4)	276.39^b^
**4-Sr**	2.8312(12)	2.4203(11)	2.4443(10)	0.0231(6)	282.59^b^
**4-Ba**	3.039(2)	2.569(2)	2.568(2)	0.2119(12)	298.63^c^
**7**	2.641(2)	2.257(2)	2.306(2)	0.0734(11)	
**8**	2.665(2)	2.2973(13)	2.3119(13)	0.0134(9)	277.21^a^^,^^c^

a Referenced to residual C_6_D_6_ at 100 MHz instrument frequency.

b Referenced to residual C_6_D_6_ at 125 MHz instrument frequency.

c Resonance only observed in HMBC experiments.

**Figure 6:**
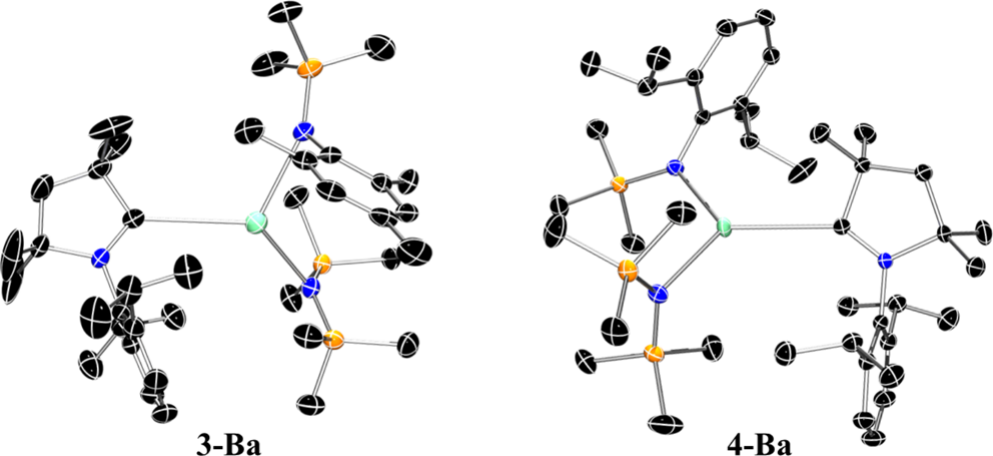
Crystal structure of **3-Ba** (left) and **4-Ba** (right). Ellipsoids are set at 50% probability level. Hydrogen atoms have been omitted for clarity. Legend: carbon (black), nitrogen (blue), silicon (orange), barium (aquamarine).

The heaviest congeners **3-Ba** and **4-Ba** crystallize in the monoclinic space groups *P*2_1_/*c* and *P*2_1_/*n*, respectively (Figure 6). The Ba–C_CAAC_ bond lengths in **3-Ba** [3.069(3) Å] and **4-Ba** [3.039(2) Å] are slightly shorter than those of the four-coordinate [Ba(N″)_2_(CAAC^Me^)(THF)] [**IV**, 3.108(3) Å] and [Ba(N″)_2_(CAAC^Cy^)(THF)] [**V**, 3.121(2) Å]; it is noteworthy that coordination of THF to barium in **IV** and **V** reduces the electropositivity about the Ba^2+^ center, resulting in the elongation of the metal–carbene bond. The 3-coordinate barium centers in **3-Ba** and **4-Ba** exhibit a slightly distorted trigonal planar geometry with bond angles of 114.5(7)–124.78(7)° (**3-Ba**) and 110.78(4)–128.30(4)° (**4-Ba**), together with a slight deviation of the Ba^2+^ cation from the N^∧^N^∧^C plane [**3-Ba:** 0.3752(14) Å; **4-Ba:** 0.2119(12) Å]. The relative conformation of the carbene and the aryl(silyl)amide ligands about the Ba center in **3-Ba** and **4-Ba** is similar and does not exhibit the same variation observed for the lighter calcium and strontium congeners. In both **3-Ba** and **4-Ba**, the aryl groups of the amide ligand and the carbene adopt positions opposite to one another with N_Ar_–Ba–C–N_CAAC_ torsion angles of −154.3(2)° and −136.4(1)°, respectively. This is likely to maximize the steric effects and saturate the large coordination sphere of the Ba^2+^ center.

### 2.3 Synthesis and Characterization of Bis(amide) CAAC Complexes **7** and **8**

**Scheme 7:**
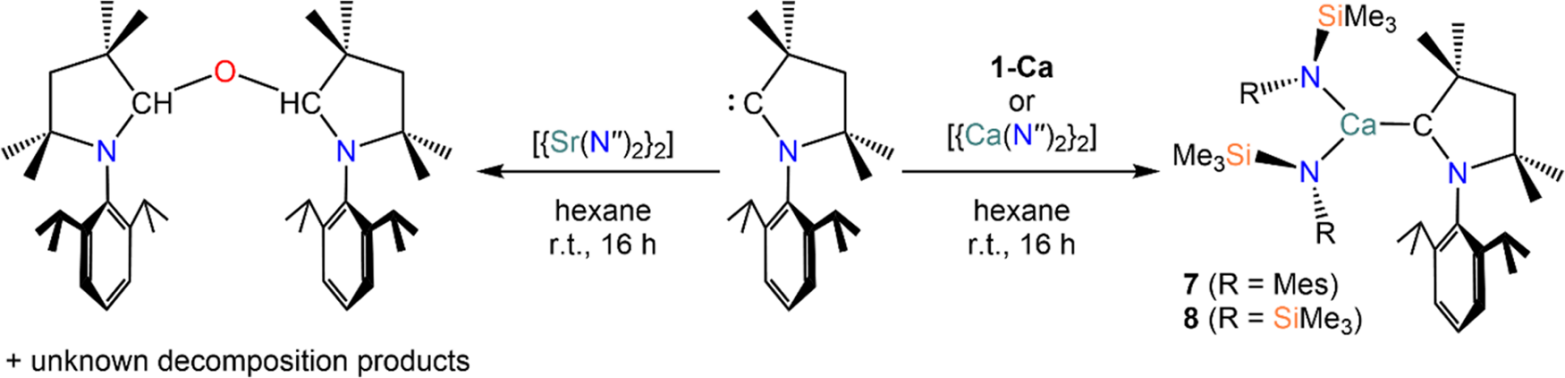
Synthesis of Complexes **7** and **8**

Following the drawbacks of forming CAAC *in situ* that led to the serendipitous isolation of mixed-amide complexes **3-AE** and **4-AE**, we decided to investigate an alternative route to target carbene adducts of bis(amide) complexes, [AE{N(Ar)(SiMe_3_)}_2_(CAAC)]. For this purpose, we decided to employ the solvent-free bis(amide) complexes **1-AE** (AE = Ca, Sr) and prepare the corresponding CAAC complexes from the equimolar reaction between the free carbene and the bis(amide) starting material (Scheme 7). The reaction between **1-Ca** and CAAC in hexane yielded colorless crystals of [Ca{N(Mes)(SiMe_3_)}_2_(CAAC)] (**7**) as determined by XRD studies (*vide infra*). NMR characterization of **7** from a crystalline sample gave spectra reminiscent to that of **4-Ca**, with largely broad and undecipherable resonances in the ^1^H NMR spectra and no observed characteristic resonance for the carbene carbon (either free or coordinated) in the ^13^C spectra. To further probe the utility of solvent-free starting materials in this methodology, the bis-N″ analogue [Ca(N′′)_2_(CAAC)] (**8**) was prepared following the same protocol, yielding colorless crystals suitable for XRD studies from a saturated hexane solution at −30 °C (Scheme 7). It is noteworthy that previously reported attempts to obtain **8** afforded only intractable mixtures.^4,9^ Characterization via NMR spectroscopy confirms the coordination to calcium, exhibiting an upfield-shifted carbene resonance of 277.2 ppm in the ^13^C NMR spectra, comparable to those of **3-Ca** (276.5 ppm) and **4-Ca** (276.4 ppm). Unlike the other Ca analogues **3-Ca**, **4-Ca**, and **7**, the ^1^H NMR spectrum of **8** is well-resolved indicating complete free-rotation of the carbene ligand in solution at room temperature, and is in complete agreement with the observed trend between the resolution of the ^1^H NMR spectra with increasing steric bulk.

Attempts to prepare the Sr analogue via addition of CAAC to **1-Sr** fell short of isolating the desired species. Upon addition of CAAC to a C_6_D_6_ suspension of **1-Sr**, complete dissolution of the starting materials occurs, suggesting complexation and the formation of a more soluble CAAC-bearing species. Furthermore, when performing the reaction under equimolar conditions the characteristic downfield ^13^C NMR signal of the free-carbene is not observed, thus further supporting this conclusion. Despite these observations, repeated attempts to isolate the target Sr-CAAC complex yielded only the starting materials, or, in some cases, the known hydrolysis product of the carbene ligand [(CAAC^H^)_2_O] (Scheme 7).^43^

**Figure 7:**
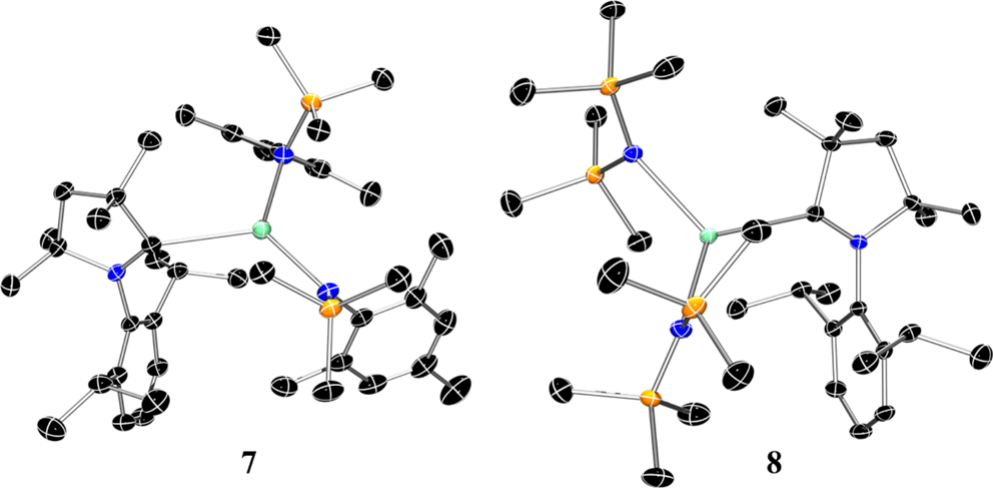
Crystal structures of **7** (left) and **8** (right). Ellipsoids are set at 50% probability level. Hydrogen atoms have been omitted for clarity. Legend: carbon (black), nitrogen (blue), silicon (orange), calcium (aquamarine).

Complex **7** crystallizes in the monoclinic *C*2/*c* space group (Figure 7). The Ca–C_CAAC_ bond distance in **7** [2.641(2) Å] is comparable to that of the mixed-amide analogue **3-Ca** and slightly shorter than the Ca–C_CAAC_ distance in **4-Ca** [2.677(7) Å]. Similarly to **3-Ca** and **4-Ca**, the geometry of the calcium center in **7** is slightly distorted trigonal planar with bond angles ranging between 113.77(6)–123.24(7)° and only slight deviation of the Ca^2+^ cation from the N^∧^N^∧^C plane [0.0733(12) Å]. The Ca–N bond distances of complex **7** [2.257(2) and 2.306(2) Å] are comparable to those of **3-Ca** [2.2750(11)–2.3061(11) Å] and **4-Ca** [2.279(6) and 2.306(6) Å], which suggests that the N″ with a {N(Mes)(SiMe_3_)}^−^ ligands impart a similar level of steric crowding around the metal center. Moreover, complex **8** crystallizes in the monoclinic *P*2_1_/*c* space group (Figure 7). The Ca–C_CAAC_ bond distance of **8** [2.665(2) Å] is consistent with all aforementioned Ca–CAAC complexes (**3-Ca**, **4-Ca**, and **7**) and the closely related complex **VI**.^4^ The Ca–N bond distances of **8** [2.297(1) and 2.312(1) Å] are also comparable (Table 1). The calcium center adopts a distorted trigonal planar geometry with angles in the range of 115.61(5)–127.63(5)°, deviating from the ideal N^∧^N^∧^C coordination plane by 0.0133(9) Å.

### 2.4 Mechanochemical Reductions and DFT Calculations

**Scheme 8:**
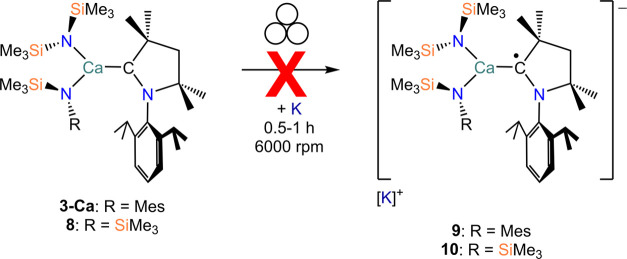
Attempted Reduction of **3-Ca** and **8** Using Mechanochemical Methods

Taking inspiration from recent mechanochemistry applications for the reduction of group 2 complexes,^10,44^ we investigated the use of a solvent-free, mechanochemical protocol to reduce CAAC adducts **3-Ca** and **8** (Scheme 8), targeting the isolation of radical complexes [K][Ca{N(Mes)(SiMe_3_)}(N″)(CAAC)] (**9**) or [K][Ca(N″)_2_(CAAC)] (**10**). Inside an argon-filled glovebox, a 25 mL poly(tetrafluoroethylene) (PTFE) milling jar was charged with, **3-Ca**, potassium metal and three stainless steel ball bearings (3 mm). The reaction mixture was milled at 6000 rpm at room temperature. After 30 min, all of the metal had been evenly dispersed and the formation of a homogeneous, blue powder was observed. Milling for a further 30 min resulted in the formation of a metallic ball (presumed to be elemental potassium) and a brown sticky solid. Pentane portions were used to extract the soluble components, and slow evaporation of the pentane extracts yielded colorless crystals of the free carbene CAAC. Similar observations were made when performing the analogous reduction of compound **8**. An aliquot was removed after milling the mixture for 30 min, and ^1^H NMR analysis showed an intractable mixture of products that included the free carbene and [{Ca(N″)_2_}_2_]. No other products were identified from the product mixture.

**Figure 8:**
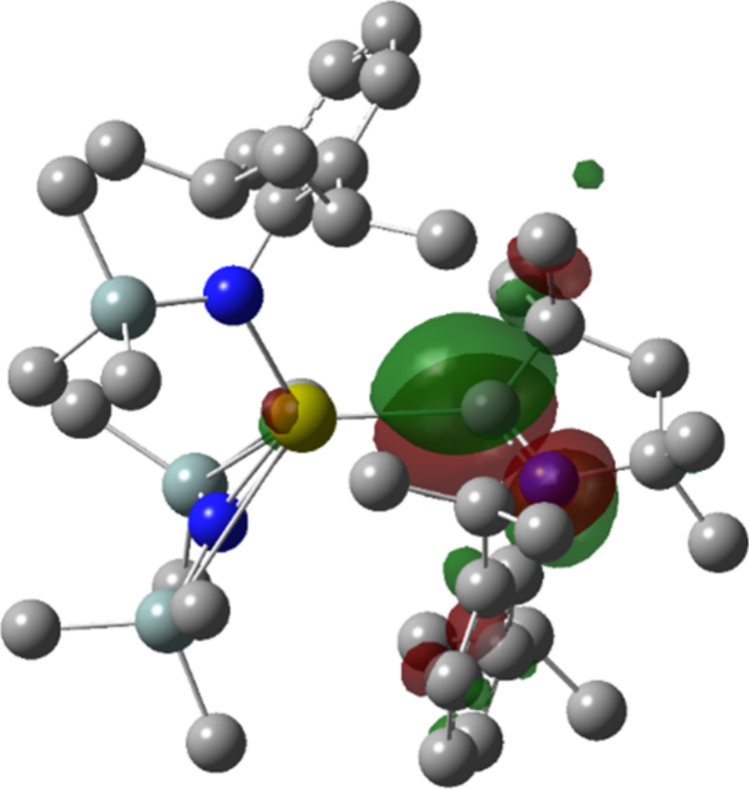
B3PW91/BS2 LUMO of **4-Ca**. Surfaces displayed at isovalue of 0.04, hydrogen atoms omitted for clarity.

Since no successful reductions of these new AE-CAAC complexes could be achieved, putative reduced species [AE(L)_2_(CAAC)]^−^ (Scheme 1) were modeled *in silico* (B3PW91/BS1). The bonding metrics of the optimized structures of **3-AE** and **4-AE** are slightly elongated compared to those in the crystal structures, which could be attributed to crystal packing effects^45^ or due to the use of a hybrid functional. Following the one-electron reduction of **3-AE**, **4-AE**, **7**, and **8**, the AE–N bond lengths tend to increase by approximately 0.05–0.08 Å, while the AE–C bond lengths tend to decrease by 0.16–0.25 Å (Table S8). The slight increase in the AE–N bond lengths could be attributed to the CAAC ligand moving closer to the metal and increasing the steric demand, thereby pushing the amide ligands further away. The shortening of the AE–C bond was observed experimentally by Harder and co-workers with the reduction of [Mg(Am)(CAAC)(I)].^46^ An approximate trend can also be seen down the group wherein the change in the AE–N bond length increases with heavier metals with a concurrent decrease in the AE–C bond length. NBO analyses (B3PW91/BS2) of **3-AE**, **4-AE**, **7**, and **8** (Tables S5–S7 and Figure 8) show that the LUMO consists of a π* antibonding orbital on the C–N bond of the CAAC ligand. The orbital is mainly carbon-centered and polarized toward the metal.

**Figure 9:**
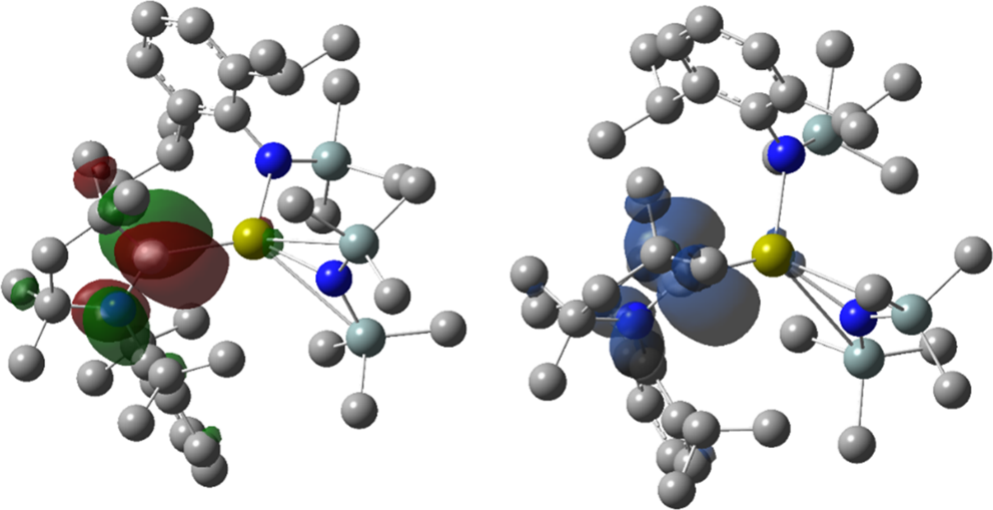
B3PW91/BS2 α-SOMO (left) and the spin density plot (right) of [Ca{N(Mes)(SiMe_3_)}_2_(CAAC)]^−^. Surfaces displayed at isovalue of 0.04, hydrogen atoms omitted for clarity.

In the case of the reduced analogues [AE(L)_2_(CAAC)]^−^, the α-SOMO (Figure 9 and Tables S5–S7) replicates the LUMO of the neutral precursor. The β-SOMO instead displays an AE–C_carbene_ π* antibonding orbital significantly polarized toward C. The α- and β-LUMO orbitals are ubiquitously π* antibonding orbitals on the CAAC phenyl ring. The spin density plots of [AE(L)_2_(CAAC)]^−^ are very similar to the LUMO of [AE(L)_2_(CAAC)] and the α-SOMO of [AE(L)_2_(CAAC)]^−^. This strongly suggests that in the event of a one-electron reduction of [AE(L)_2_(CAAC)], the additional electron would occupy the π* orbital localized on the carbene across the C–N bond.

A survey of the NPA charges on the metals reveals no change in charge at Ca, Sr or Ba, upon reduction (Tables S9–S11). The NPA charge on C_carbene_ increases from neutral to ca. −0.5, while the adjacent N_carbene_ gains around 0.14 charge units (from −0.48 to −0.62). These data imply the additional electron is predominantly located on the CAAC carbene and between these two atoms, matching the spin density plots (*vide supra*).

The second-order perturbation analysis in the homologous series of [AE{N(Mes)(SiMe_3_)} (N″)(CAAC)] (**3-AE**) and [AE{N(Dipp)(SiMe_3_)}(N″)(CAAC)] (**4-AE**), sees the carbene bind more strongly to the metal cation as the group is descended (Table 2). However, there is no clear trend in changes to the hybridization of the NBO acceptor orbital in these compounds.

**Table 2:** Second-Order Perturbation Energies from NBO Analysis (in kcal mol^–1^) between the Carbene NBO Lone Pair and the Vacant NBO of the AE Atom (B3PW91/BS2) in [AE(L)_2_(CAAC)] (**A**) and [AE(L)_2_(CAAC)]^−^ (**B**)

	**A**	**α-B**	**β-B**
**3-Ca**	8.9	6.4	7.3
**3-Sr**	9.7	7.5	7.7
**3-Ba**	11.6	13.2	14.6
**4-Ca**	6.6	3.1	3.2
**4-Sr**	6.5	2.7	2.8
**4-Ba**	10.6	11.6	11.4
**7 (AE = Ca)**	12.3	8.8	9.0
**8 (AE = Ca)**	8.7	7.0	7.6

Analysis of the hybridization states of the acceptor orbitals of the AE center (excluding Rydberg orbitals) reveals that these are >97.5% *s*-character, as expected (Table 3). Upon one-electron reduction of the complexes, the *d*-orbitals of these metals become tenuously involved in bonding. Interestingly, participation for Ba maximizes in the case of [Ba{N(Mes)(SiMe_3_)}(N″)(CAAC)] at 10.67% for the α-orbitals, and [Ca{N(Mes)(SiMe_3_)}_2_(CAAC)] displays an unusually high involvement of the *d*-manifold. The increased charge of the CAAC ligand therefore has an effect on the electronic structure of the AE dications, yielding a significant increase in the *d*-character of the metal centers.

**Table 3:** NBO Contributions % (*s*, *p*, *d*) in Metal Acceptor Orbitals for [AE(L)_2_(CAAC)] (**A**) and [AE(L)_2_(CAAC)]^−^ (**B**) (B3PW91/BS2)

	**A**			**α-B**			**β-B**		
	*s*	*p*	*d*	*s*	*p*	*d*	*s*	*p*	*d*
**3-Ca**	97.6	0.1	2.3	95.6	0.1	5.3	92.0	0.2	7.8
**3-Sr**	98.8	0.1	1.1	96.7	0.2	3.1	95.8	0.2	4.0
**3-Ba**	98.3	0.1	1.6	88.4	0.7	10.7	90.3	0.4	9.0
**4-Ca**	98.9	0.0	1.1	97.2	0.1	2.7	96.0	0.0	4.0
**4-Sr**	99.1	0.0	0.9	97.9	0.1	2.0	97.1	0.1	2.8
**4-Ba**	98.8	0.0	1.2	91.4	0.3	8.1	94.1	0.2	5.5
**7 (AE = Ca)**	97.7	0.0	2.3	92.0	0.5	7.5	93.1	0.1	6.8
**8 (AE = Ca)**	98.0	0.2	1.2	95.1	0.3	4.6	94.0	0.2	5.8

## 3 Conclusions

We report the synthesis of 8 novel, heavy AE-CAAC complexes featuring sterically bulk amide ligands. The mixed-amide complexes **3-AE** and **4-AE** (AE = Ca–Ba) were formed via an unexpected protonolysis reaction following *in situ* generation of the free carbene; we envisage that this method could be a useful synthetic tool for the preparation of heteroleptic monodentate amide complexes, which are notoriously difficult to synthesize. Moreover, complexes **7** and **8** were prepared by complexation of the free carbene to the respective Lewis base-free bis(amide) complexes. The frontier molecular orbitals of **3-AE**, **4-AE**, **7**, and **8** were interrogated by a DFT computational study which revealed that, although all of the precursor complexes satisfy our molecular design (i.e., trigonal planar coordination geometry), the desired *d*-manifold remains energetically less accessible. Instead, the LUMO of all of the complexes is situated on the CAAC ligand framework owing to the electrophilicity of the carbene ligand, therefore suggesting that an alternative ligand may be more appropriate for this application.

## 4 Experimental Section

### 4.1 General Methods

THF, Et_2_O, toluene, hexane, and pentane were passed through columns containing molecular sieves, then stored over 4 Å molecular sieves (THF) or over a potassium mirror (Et_2_O, hexane, toluene, pentane), and thoroughly degassed prior to use. For NMR spectroscopy, C_6_D_6_ and C_4_D_8_O were dried by refluxing over potassium, before being degassed by three freeze–pump–thaw cycles and transferred by vacuum transfer. NMR spectra were recorded on either a Bruker AVIII HD 400 or Bruker AVIII 500 spectrometer operating at 400.07/500.13 (^1^H), 100.60/125.78 (^13^C{^1^H}), or 79.48 (^29^Si{^1^H}). To achieve a greater signal-to-noise ratio, the ^29^Si{^1^H} NMR spectra were acquired with a DEPT24 pulse sequence. NMR spectra were recorded at 298 K unless otherwise stated and were referenced to residual solvent signals in the case of ^1^H and ^13^C{^1^H} experiments. ^1^H–^13^C HMBC experiments were used to detect ^13^C resonances arising from the coordinated carbene center. FTIR spectra were recorded on a Bruker α II spectrometer with Platinum-ATR module. Elemental microanalyses were carried out by London Metropolitan University. All AE iodides were baked at 200 °C for 4 h prior to use. HN(Mes)(SiMe_3_), HN(Dipp)(SiMe_3_), K[N(Mes)(SiMe_3_)], K[N(Dipp)(SiMe_3_)], K(N″), Ca(N″)_2_, and HCl·CAAC were prepared according to literature procedures.^4,47−51^ Solid state reactions were performed using an IKA ULTRA-TURRAX Tube Drive Disperser; 20 mL polypropylene milling jar, 4 × 3 mm stainless steel ball bearings (316 stainless steel, 0.5 g).

### 4.2 Synthesis of AE Bis(amide) Species **1-AE·(THF)_2_**, **1-AE**, and **5**

The THF-solvated AE bis(amide) starting materials [AE{N(Ar)_2_}_2_(THF)_2_] were prepared according to a modified literature procedure.^32^

#### 4.2.1 [AE{N(Ar)_2_}_2_(THF)_2_] (**1-AE·(THF)**_**2**_: AE = Ca–Ba, Ar = Mes; **2-AE·(THF)**_**2**_: AE = Ca–Ba, Ar = Dipp)

AEI_2_ (1–2 mmol, 1 equiv) and K[N(Ar)(SiMe_3_)] (2–4 mmol, 2 equiv) were combined in THF (30 mL) and the resulting colorless suspension was stirred at room temperature for 16 h. The reaction mixture was filtered and a further 10 mL of THF was used to wash the insoluble material. All volatiles were removed from the combined filtrates *in vacuo* and the resulting crude material was fully dried under reduced pressure with gentle heating (<10^–2^ mbar, 30 °C) yielding **1-AE·(THF)**_**2**_ and **2-AE·(THF)**_**2**_ as colorless solids (AE = Ca, Sr) except for **1-Ba·(THF)**_**2**_, which gave a brown oil that solidified upon storing at room temperature overnight.

#### 4.2.2 [Ca{N(Mes)(SiMe_3_)}_2_(THF)_2_] (**1-Ca·(THF)**_**2**_)

Yield = 0.970 g, 1.6 mmol, 81.4%. Characterization data in agreement with previous reports.^33^

#### 4.2.3 [Sr{N(Mes)(SiMe_3_)}_2_(THF)_2_] (**1-Sr·(THF)**_**2**_)

Yield = 0.811 g, 1.3 mmol, 63%. Characterization data in agreement with previous reports.^52^

#### 4.2.4 [Ba{N(Mes)(SiMe_3_)}_2_(THF)_2_] (**1-Ba·(THF)**_**2**_)

Yield = 0.643 g, 0.9 mmol, 66.2%.

^1^H NMR (400 MHz, 298 K, C_6_D_6_): δ/ppm = 0.40 (s, 18H, Si(C*H*_3_)_3_), 1.25 (m, 8H, THF–C*H*_2_), 2.24 (s, 6H, *p*–C*H*_3_), 2.30 (s, 12H, *o*–C*H*_3_), 3.15 (m, 8H, THF-OC*H*_2_), 6.94 (s, 4H, *m*-C_6_*H*_2_(CH_3_)_3_). ^13^C{^1^H} NMR (100 MHz, 298 K, C_6_D_6_): δ/ppm = 4.7 (Si(*C*H_3_)_3_), 20.9 (*p*-*C*H_3_), 21.0 (*o*-*C*H_3_), 25.3 (THF-*C*H_2_), 68.1 (THF-O*C*H_2_), 125.0 (*p*-*C*_6_H_2_(CH_3_)_3_), 129.9 (Ar-*C*), 133.0 (Ar-*C*), 152.4 (*i*-*C*_6_H_2_(CH_3_)_3_). ^29^Si{^1^H} NMR (80 MHz, 298 K, C_6_D_6_): δ/ppm = −19.48.

#### 4.2.5 [Ca{N(Dipp)(SiMe_3_)}_2_(THF)_2_] (**2-Ca·(THF)**_**2**_)

Yield = 1.13 g, 1.6 mmol, 79.2%.

^1^H NMR (400 MHz, 298 K, C_6_D_6_): δ/ppm = 0.42 (s, 18H, Si(C*H*_3_)_3_), 1.10 (m, 8H, THF–C*H*_2_), 1.28 (m, 24H, CH(C*H*_2_)_2_), 3.11 (m, 8H, THF-OC*H*_2_), 3.97 (sept., 4H, ^3^*J*_HH_ = 6.88 Hz, C*H*(CH_3_)_2_), 6.89 (t, 2H, ^3^*J*_HH_ = 7.68 Hz, *p*-C_6_*H*_3_(^i^Pr)_2_), 7.10 (d, 4H, ^3^*J*_HH_ = 7.68 Hz, *m*-C_6_*H*_3_(^i^Pr)_2_). ^13^C{^1^H} NMR (100 MHz, 298 K, C_6_D_6_): δ/ppm = 4.7 (Si(*C*H_3_)_3_), 25.0 (THF-*C*H_2_), 25.8 (CH(*C*H_3_)_2_), 27.3 (*C*H(CH_3_)_2_), 69.4 (THF-O*C*H_2_), 118.5 (*p*-*C*_6_H_3_(^i^Pr)_2_), 123.5 (*m*-*C*_6_H_3_(^i^Pr)_2_), 143.9 (*o*-*C*_6_H_3_(^i^Pr)_2_), 153.5 (*i*-*C*_6_H_3_(^i^Pr)_2_). ^29^Si{^1^H} NMR (80 MHz, 298 K, C_6_D_6_): δ/ppm = −14.53.

#### 4.2.6 [Sr{N(Dipp)(SiMe_3_)}_2_(THF)_2_] (**2-Sr·(THF)**_**2**_)

Yield = 0.560 g, 0.8 mmol, 73.2%. Characterization data in agreement with previous reports.^32^

#### 4.2.7 [Ba{N(Mes)(SiMe_3_)}_2_(THF)_2_] (**2-Ba·(THF)**_**2**_)

Yield = 0.927 g, 1.2 mmol, 68.0%. Characterization data in agreement with previous reports.^32^

#### 4.2.8 [{AE[N(Mes)(SiMe_3_)][μ-N(Mes)(SiMe_3_)]}_2_] (**1-AE = Ca, Sr**)

AEI_2_ (1–2 mmol, 1 equiv) and K[N(Mes)(SiMe_3_)] (2–4 mmol, 2 equiv) were combined in diethyl ether (30 mL) and the resulting colorless suspension was stirred at room temperature for 48 h. The reaction mixture was filtered and a further 15 mL of diethyl ether was used to wash the insoluble material. All volatiles were removed from the combined filtrates *in vacuo* and the resulting oily solid was fully dried under reduced pressure with gentle heating (<10^–2^ mbar, 30 °C) yielding **3.1-AE** as a colorless solid.

#### 4.2.9 [{Ca[N(Mes)(SiMe_3_)][μ-N(Mes)(SiMe_3_)]}_2_] (**1-Ca**)

Yield = 0.190, 0.41 mmol, 42%. Single crystals suitable for XRD were obtained from a saturated hexane solution (0.45 g in 10 mL) at 5 °C.

^1^H NMR (400 MHz, 298 K, C_6_D_6_): δ/ppm = 0.17 (s, 9H, Si(C*H*_3_)_3_), 0.19 (s, 9H, Si(C*H*_3_)_3_), 1.96 (s, 3H, *p*–C*H*_3_), 2.26 (s, 6H, *o*–C*H*_3_), 2.29 (s, 6H, *o*–C*H*_3_), 2.34 (s, 3H, *p*–C*H*_3_), 6.34 (s, 2H, *m*-C_6_*H*_2_(CH_3_)_3_), 6.98 (s, 2H, *m*-C_6_*H*_2_(CH_3_)_3_). ^13^C{^1^H} NMR (100 MHz, 298 K, C_6_D_6_): δ/ppm = 4.0 (Si(*C*H_3_)_3_), 5.5 (Si(*C*H_3_)_3_), 19.8, 21.0, 21.8, 24.3, 126.3 (*p-C*), 126.9 (*p-C*), 129.8 (*m*-*C*), 130.1 (*m-C*), 133.1 (*p-C*), 133.8 (*p-C*), 152.5 (*i-C*), 152.7 (*i-C*). ^29^Si{^1^H} NMR (80 MHz, 298 K, C_6_D_6_): δ/ppm = −14.52, −13.35. Addition of C_4_D_8_O to the sample gives rise to the highly symmetric spectra of **1-Ca·(THF)**_**2**_. Elemental Anal. Calcd for: C_24_H_40_CaN_2_Si_2_: C, 63.66%; H, 8.90%; N, 6.19%. Found: C, 61.19%; H, 8.48%; N, 5.92%. FTIR: υ/cm^–1^ = 2942 (w), 1466 (m), 1418 (m), 1310 (s), 1297 (s), 1249(s), 1158 (w), 965 (m), 914 (s), 881 (m), 816 (s), 764 (m), 738 (m), 664 (m), 613 (m), 566 (w), 473 (s).

#### 4.2.10 [{Sr[N(Mes)(SiMe_3_)][μ-N(Mes)(SiMe_3_)]}_2_] (**1-Sr**)

Yield = 0.500, 1.00 mmol, 48.9%. Single crystals suitable for XRD were obtained from a saturated hexane solution (0.15 g in 15 mL) at room temperature.

^1^H NMR (500 MHz, 298 K, C_6_D_6_): δ/ppm = 0.26 (s, 18H, Si(C*H*_3_)_3_), 2.22 (br s, 18H, C_6_H_2_(C*H*_3_)_3_), 6.25 (br s, 2H, *m*-C_6_*H*_2_(CH_3_)_3_), 6.98 (br s, 2H, *m*-C_6_*H*_2_(CH_3_)_3_). No satisfactory ^13^C{^1^H} or ^29^Si{^1^H} NMR spectra could be obtained for **1-Sr** due to its poor solubility in benzene. Addition of C_4_D_8_O to the sample gives rise to the highly symmetric spectra of **1-Sr·(THF)**_**2**_. Elemental Anal. Calcd for: C_24_H_40_SrN_2_Si_2_: C, 57.61%; H, 8.06%; N, 5.60%. Found: C, 57.72%; H, 7.96%; N, 5.28%. FTIR: υ/cm^–1^ = 2942 (w), 1467 (m), 1418 (m), 1309 (s), 1297 (s), 1249 (s), 1158 (w), 965 (s), 925 (s), 914 (w), 881 (w), 816 (s), 764 (m), 738 (m), 664 (m), 613 (w), 566 (w), 473 (s).

#### 4.2.11 [{Ca{N(Dipp)(SiMe_3_)}_2_(μ-N″)}_2_] (**5**)

Isolated as single crystals from the reaction to form **4-Ca** as a small crystalline crop of **5** (<10 mg) sufficient only for XRD and NMR characterization. ^1^H NMR (400 MHz, 298 K, C_6_D_6_): δ/ppm = 0.19 (s, 18H, Si(C*H*_3_)_3_), 0.35 (s, 9H, Si(C*H*_3_)_3_), 1.21 (br, m, 12H, CH(C*H*_3_)_2_), 3.69 (sept, 2H, ^3^*J*_HH_ = 6.84 Hz, C*H*(CH_3_)_2_), 6.94 (t, 1H, ^3^*J*_HH_ = 7.52 Hz, *p-*C_6_*H*_3_(^i^Pr)_2_), 7.10 (d, 2H, ^3^*J*_HH_ = 7.52 Hz, *m-*C_6_*H*_3_(^i^Pr)_2_). ^13^C{^1^H} NMR (100 MHz, 298 K, C_6_D_6_): δ/ppm = 4.0 (Si(*C*H_3_)_3_), 5.2 (Si(*C*H_3_)_3_), 24.9 (CH(*C*H_3_)_2_), 26.0 (CH(*C*H_3_)_2_), 27.3 (*C*H(CH_3_)_2_), 119.5 (*p-C*_6_H_3_(^i^Pr)), 124.0 (*m-C*_6_H_3_(^i^Pr)_2_), 144.7 (*o-C*_6_H_3_(^i^Pr)_2_), 151.7 (*i-C*_6_H_3_(^i^Pr)_2_; signal observed from 2D ^1^H–^13^C HMBC experiments). ^29^Si{^1^H} NMR (80 MHz, 298 K, C_6_D_6_): δ/ppm = −16.52, −14.44.

### 4.3 Synthesis of AE CAAC Complexes **3-AE** and **4-AE**

#### 4.3.1 [AE{N(Ar)(SiMe_3_})(N″)(CAAC)] (**3-AE**: AE = Ca–Ba, Ar = Mes; **4-AE**: AE = Ca–Ba, Ar = Dipp)

HCl·CAAC (0.644 g, 2 mmol, 1 equiv) and K(N″) (0.399 g, 2 mmol, 1 equiv) were combined in a Schlenk flask before benzene (30 mL) was added. The resulting suspension was sonicated for 15 min and stirred for a further 2 h at room temperature before being filtered into a flask containing **1-AE·(THF)**_**2**_ or **2-AE·(THF)**_**2**_ (1 mmol, 0.5 equiv). The reaction mixture was stirred overnight, the volatile components removed *in vacuo*, and the resulting oily residue dried thoroughly under reduced pressure with heating (<10^–2^ mbar, 65 °C). Pentane was used to extract the products, from which colorless crystals of **3-AE** or **4-AE** were obtained from either slow evaporation or cooling down of the pentane extracts.

#### 4.3.2 [Ca{N(Mes)(SiMe_3_)}(N″)(CAAC)] (**3-Ca**)

Yield = 0.225 g, 0.3 mmol, 32.4%. ^1^H NMR (400 MHz, 298 K, C_6_D_6_): δ/ppm = 0.08 (s, 18H, N″-Si(C*H*_3_)_3_), 0.41 (s, 9H, NMes-Si(C*H*_3_)_3_), 0.82 (s, 6H, C(C*H*_3_)_2_), 0.88 (t, 4.75 H, ^3^*J*_*HH*_ = 6.97 Hz, C_5_H_12_–C*H*_3_), 1.03 (d, 6H, ^3^*J*_HH_ = 6.33 Hz, (C*H*_3_)_2_CH), 1.16 (d, 6H, ^3^*J*_HH_ = 6.60 Hz, (C*H*_3_)_2_CH), 1.21–1.29 (m, 4.75H, C_5_H_12_–C*H*_2_), 1.24 (s, 2H, C*H*_2_), 1.37 (s, 6H, C(C*H*_3_)_2_), 2.27 (s, 3H, *p*–C*H*_3_), 2.42 (s, 6H, *o*–C*H*_3_), 2.55 (br sept., 2H, (CH_3_)_2_C*H*), 6.88–6.95 (m, 2H, *m-*C_6_*H*_3_(^i^Pr)_2_), 6.97 (s, 2H, *m*-C_6_*H*_2_(CH_3_)_3_), 7.03 (m, 1H, *p*-C_6_*H*_3_(^i^Pr)_2_). ^13^C{^1^H} NMR (100 MHz, 298 K, C_6_D_6_): δ/ppm = 4.9 (NMes-Si(*C*H_3_)_3_), 5.4 (N″-Si(*C*H_3_)_3_), 14.3 (C_5_H_12_-*C*H_3_), 20.9 (*p*-*C*H_3_), 22.4 (*o*-*C*H_3_), 22.7 (C_5_H_12_-*C*H_2_), 23.9 ((*C*H_3_)_2_CH), 28.5 ((*C*H_3_)_2_CH), 28.9 (C(*C*H_3_)_2_), 29.0 (C(*C*H_3_)_2_), 34.5 (C_5_H_12_-*C*H_2_), 50.3 (*C*H_2_), 56.9 (*C*(CH_3_)_2_), 83.1 (*C*(CH_3_)_2_), 125.6 (*p*-*C*_6_H_3_(^i^Pr)_2_), 127.0 (*p*-*C*_6_H_2_(CH_3_)_3_), 129.6 (*C*_6_H_3_(^i^Pr)_2_), 130.3 (*m*-*C*_6_H_2_(CH_3_)_3_), 130.8 (*C*_6_H_3_(^i^Pr)_2_) 134.6 (*o*-*C*_6_H_2_(CH_3_)_3_), 144.9 (*i*-*C*_6_H_3_(^i^Pr)_2_), 152.2 (*i*-*C*_6_H_2_(CH_3_)_3_), 276.5 (C_Carbene_). ^29^Si{^1^H} NMR (80 MHz, 298 K, C_6_D_6_): δ/ppm = −16.11, −15.00. Elemental Anal. Calcd for: C_38_H_69_CaN_3_Si_3_: C, 65.93%; H, 10.05%; N, 6.07%. Found: C, 65.75%; H, 9.89%; N, 5.73%. FTIR: υ/cm^–1^ = 2969 (w), 2946 (m), 2873 (w), 1462 (m), 1298 (w), 1248 (s), 1234 (s), 1160 (w), 1043 (s), 969 (m), 934 (m), 878 (s), 817 (s), 769 (s), 739 (m), 662 (s), 607 (w), 591 (w), 564 (w), 481 (m).

#### 4.3.3 [Sr{N(Mes)(SiMe_3_)}(N″)(CAAC)] (**3-Sr**)

Yield = 0.384 g, 0.5 mmol, 45.1%. ^1^H NMR (400 MHz, 298 K, C_6_D_6_): δ/ppm = 0.15 (s, 18H, N″-Si(C*H*_3_)_3_), 0.42 (s, 9H, NMes-Si(C*H*_3_)_3_), 0.82 (br s, 6H, C(C*H*_3_)_2_), 1.08 (m, 12H, (C*H*_3_)_2_CH), 1.27 (br s, 2H, C*H*_2_), 1.30 (br s, 6H, C(C*H*_3_)_2_), 2.25 (s, 3H, *p*–C*H*_3_), 2.31 (s, 6H, *o*–C*H*_3_), 2.66 (br m, 2H, (CH_3_)_2_C*H*), 6.90–7.11 (m, 5H, Ar-*H*). ^13^C{^1^H} NMR (100 MHz, 298 K, C_6_D_6_): δ/ppm = 5.1 (NMes-Si(*C*H_3_)_3_), 5.2 (N″-Si(*C*H_3_)_3_), 20.9 (*p*-*C*H_3_), 21.8 (*o*-*C*H_3_), 22.8 ((*C*H_3_)_2_CH), 28.2 ((*C*H_3_)_2_CH), 28.9 (C(*C*H_3_)_2_), 29.0 (C(*C*H_3_)_2_), 50.1 (*C*H_2_), 56.9 (*C*(CH_3_)_2_), 82.9 (*C*(CH_3_)_2_), 125.1 (*C*_6_H_3_(^i^Pr)_2_), 126.2 (*p*-*C*(CH_3_)), 129.5 (*C*_6_H_3_(^i^Pr)_2_), 130.8 (*m*-*C*_6_H_2_(CH_3_)_3_), 131.3 (*C*_6_H_3_(^i^Pr)_2_) 134.2 (*o*-*C*_6_H_2_(CH_3_)_3_), 145.1 (*i*-*C*_6_H_3_(^i^Pr)_2_), 153.4 (*i*-*C*_6_H_2_(CH_3_)_3_), 283.2 (C_Carbene_; signal observed from 2D ^1^H–^13^C HMBC experiments). ^29^Si{^1^H} NMR (80 MHz, 298 K, C_6_D_6_): δ/ppm = −18.42, −17.49. Elemental Anal. Calcd for: C_38_H_69_SrN_3_Si_3_: C, 61.69%; H, 9.40%; N, 5.68%. Found: C, 61.67%; H, 9.28%; N, 5.48%. FTIR: υ/cm^–1^ = 2970 (w), 2944 (m), 2874 (w), 1461 (m), 1412 (w), 1299 (m), 1246 (s), 1232 (s), 1161 (w), 1065 (s), 972 (s), 935 (m), 877 (s), 808 (s), 769 (s), 746 (m), 661 (s), 605 (w), 580 (w), 470 (m).

#### 4.3.4 [Ba{N(Mes)(SiMe_3_)}(N″)(CAAC)] (**3-Ba**)

Yield = 0.167 g, 0.2 mmol, 42%. ^1^H NMR (400 MHz, 298 K, C_6_D_6_): δ/ppm = 0.23 (s, 18H, N″-Si(C*H*_3_)_3_), 0.42 (s, 9H, NMes-Si(C*H*_3_)_3_), 0.81 (s, 6H, C(C*H*_3_)_2_), 1.03 (d, 6H, ^3^*J*_HH_ = 6.87 Hz (C*H*_3_)_2_CH), 1.05 (d, 6H, ^3^*J*_HH_ = 6.87 Hz (C*H*_3_)_2_CH), 1.23 (s, 6H, C(C*H*_3_)_2_), 1.25 (s, 2H, C*H*_2_), 2.20 (s, 6H, *o*–C*H*_3_), 2.26 (s, 3H, *p*–C*H*_3_), 2.66 (sept, 2H, ^3^*J*_HH_ = 6.79 Hz, (CH_3_)_2_C*H*), 6.97 (d, 2H, ^3^*J*_HH_ = 7.67 Hz, *m*-C_6_*H*_3_(^i^Pr)_2_), 6.99 (br s, 2H, *m*-C_6_*H*_2_(CH_3_)_3_), 7.09 (m, 1H, *p*-C_6_*H*_3_(^i^Pr)_2_). ^13^C{^1^H} NMR (100 MHz, 298 K, C_6_D_6_): δ/ppm = 5.1 (N″-Si(*C*H_3_)_3_), 5.2 (NMes-Si(*C*H_3_)_3_), 20.9 (*p*-*C*H_3_), 21.3 (*o*-*C*H_3_), 22.5 ((*C*H_3_)_2_CH), 27.9 ((*C*H_3_)_2_C), 28.1 (CH(*C*H_3_)_2_), 28.8 (*C*H(CH_3_)_2_), 28.9 (C(*C*H_3_)_2_), 50.1 (*C*H_2_), 56.8 (*C*(CH_3_)_2_), 82.8 (*C*(CH_3_)_2_), 125.1 (*p*- *C*_6_H_3_(^i^Pr)_2_), 125.8 (*p*-*C*_6_H_2_(CH_3_)_3_), 129.6 (*m*-*C*_6_H_3_(^i^Pr)_2_), 130.9 (*m*-*C*_6_H_2_(CH_3_)_3_), 134.3 (*o*-*C*_6_H_2_(CH_3_)_3_), 135.7 (*o*-*C*_6_H_3_(^i^Pr)_2_), 145.1 (*i*-*C*_6_H_3_(^i^Pr)_2_), 153.1 (*i*-*C*_6_H_2_(CH_3_)_3_), 294.8 (C_Carbene_; signal observed from 2D ^1^H–^13^C HMBC experiments). No observable signals could be obtained from ^29^Si{^1^H} NMR experiments. Elemental Anal. Calcd for: C_38_H_69_BaN_3_Si_3_: C, 57.81%; H, 8.81%; N, 5.32%. Found: C, 57.82%; H, 8.66%; N, 4.93%. FTIR: υ/cm^–1^ = 2949 (m), 2891 (w), 1458 (w), 1436(w), 1297 (m), 1263 (s), 1234 (s), 1085 (s), 968 (m), 932 (m), 874 (m), 815 (s), 768 (m), 744 (m), 660 (m), 605 (w), 563 (m), 462 (w).

#### 4.3.5 [Ca{N(Dipp)(SiMe_3_)}(N″)(CAAC)] (**4-Ca**)

An accurate yield was unobtainable due to presence of **5** which cocrystallized with **4-Ca**. The NMR spectra of the crystalline material were largely uninterpretable due to the presence of impurities and restricted rotation; see ESI.

#### 4.3.6 [Sr{N(Dipp)(SiMe_3_)}(N″)(CAAC)] (**4-Sr**)

Yield = 0.364 g, 0.47 mmol, 68%. ^1^H NMR (500 MHz, 298 K, C_6_D_6_): δ/ppm = 0.19 (s, 18H, Si(C*H*_3_)_3_), 0.39 (s, 9H, Si(C*H*_3_)_3_), 0.82 (br s, 6H, C(C*H*_3_)_2_), 0.94–1.11 (br m, 12H, CH(C*H*_3_)_2_), 1.16 (d, 6H, CH(C*H*_3_)_2_), 1.21 (m, 2H, C*H*_2_), 1.26 (br m, 6H, C(C*H*_3_)_2_), 1.34 (d, 6H, CH(C*H*_3_)_2_), 2.65 (br m, 2H, C*H*(CH_3_)_2_), 3.88 (br m, 2H, C*H*(CH_3_)_2_), 6.94 (br t, 1H, ^3^*J*_HH_ = 7.5 Hz, *p*-C_6_*H*_3_(^i^Pr)_2_), 6.99 (br d, 2H, ^3^*J*_HH_ = 6.98 Hz, *m*-C_6_*H*_3_(^i^Pr)_2_), 7.10 (br t, 1H, ^3^*J*_HH_ = 7.44 Hz, *p*-C_6_*H*_3_(^i^Pr)_2_), 7.17 (br m, 2H, *m*-C_6_*H*_3_(^i^Pr)_2_). ^13^C{^1^H} (125 MHz, 298 K, C_6_D_6_): δ/ppm = 4.8 (Si(*C*H_3_)_3_), 5.8 (Si(*C*H_3_)_3_), 6.5 (Si(*C*H_3_)_3_), 14.4, 23.1, 25.9, 27.6, 27.9, 28.9, 32.3, 50.0 (*C*H_2_), 56.7 ((CH_3_)_2_*C*), 83.1 ((CH_3_)_2_*C*), 118.4 (*p*-*C*_6_H_3_(^i^Pr)_2_), 124.0 (*m*-*C*_6_H_3_(^i^Pr)_2_), 125.4 (*m*-*C*_6_H_3_(^i^Pr)_2_), 129.5 (*p*-*C*_6_H_3_(^i^Pr)_2_), 135.8 (*i-C*_6_H_3_(^i^Pr)_2_), 143.4 (*o*-*C*_6_H_3_(^i^Pr)_2_), 145.1 (*o*-*C*_6_H_3_(^i^Pr)_2_), 152.7 (*i*-*C*_6_H_3_(^i^Pr)_2_), 282.6 (*C*_Carbene_). ^29^Si{^1^H} (99 MHz, C_6_D_6_, 298 K): δ/ppm = −18.11, −16.44. Elemental Anal. Calcd for C_41_H_75_N_3_Si_3_Sr: C, 62.98%; H, 9.67%; N, 5.37%. Found: C, 61.56%; H, 9.62%; N, 5.10%. Low carbon values were obtained consistently across different measurements and we ascribe this to carbide formation, which has been previously reported in analogous silicon-rich Group 2 complexes.^53^ FTIR: υ/cm^–1^ = 3000 (m), 2951 (w), 2867 (w), 1582 (w), 1499 (w), 1462 (m), 1411 (m), 1389 (w), 1367 (w), 1320 (m), 1242 (s), 1199 (w), 1179 (w), 1130 (w), 1079 (s), 1041 (m), 939 (s), 875 (m), 818 (s), 764 (s), 743 (s), 658 (s), 605 (m), 575 (m), 530 (w), 501 (m), 481 (w), 421 (m).

#### 4.3.7 [Ba{N(Dipp)(SiMe_3_)}(N″) (CAAC)] (**4-Ba**)

Yield = 0.240 g, 0.29 mmol, 45%.

^1^H NMR (500 MHz, 298 K, C_6_D_6_): δ/ppm = 0.21 (br s, 18H, Si(C*H*_3_)_3_), 0.38 (s, 9H, Si(C*H*_3_)_3_), 0.87 (s, 6H, C(C*H*_3_)_2_), 1.10 (*m*, 12H, CH(C*H*_3_)_2_), 1.16 (d, 6H, ^3^*J*_HH_ = 6.73 Hz, CH(C*H*_3_)_2_), 1.19 (s, 6H, C(C*H*_3_)_2_), 1.27 (d, 6H, ^3^*J*_HH_ = 6.73 Hz, CH(C*H*_3_)_2_), 1.30 (s, 2H, C*H*_2_), 2.76 (sept, 2H, ^3^*J*_HH_ = 6.73 Hz, C*H*(CH_3_)_2_), 3.74 (sept, 2H, ^3^*J*_HH_ = 6.79 Hz, C*H*(CH_3_)_2_), 6.89 (t, 1H, ^3^*J*_HH_ = 7.53 Hz, *p*-C_6_*H*_3_(^i^Pr)_2_), 7.00 (d, 2H, ^3^*J*_HH_ = 7.71 Hz, *m*-C_6_*H*_3_(^i^Pr)_2_), 7.12 (t, 1H, ^3^*J*_HH_ = 7.71 Hz, *p*-C_6_*H*_3_(^i^Pr)_2_), 7.15 (d, 2H, ^3^*J*_HH_ = 7.71 Hz, *m*-C_6_*H*_3_(^i^Pr)_2_). ^13^C{^1^H} (125 MHz, 298 K, C_6_D_6_): δ/ppm = 4.5 (Si(*C*H_3_)_3_), 5.4 (Si(*C*H_3_)_3_), 22.5 (CH(*C*H_3_)_2_), 25.6 (CH(*C*H_3_)_2_), 25.8 (CH(*C*H_3_)_2_), 27.2 (*C*H(CH_3_)_2_), 27.6(CH(*C*H_3_)_2_), 28.1 ((*C*H_3_)_2_C), 28.97 ((*C*H_3_)_2_C), 29.00 (*C*H(CH_3_)_2_), 50.4 (*C*H_2_), 57.2 (*C*(CH_3_)_2_), 82.7 ((CH_3_)_2_*C*), 118.2 (*p*-*C*_6_H_3_), 124.5 (*m*-*C*_6_H_3_(^i^Pr)_2_), 124.8 (*m*-*C*_6_H_3_(^i^Pr)_2_), 129.2 (*p*-*C*_6_H_3_(^i^Pr)_2_), 136.5 (*i*-*C*_6_H_3_(^i^Pr)_2_), 143.6 (*o*-*C*_6_H_3_(^i^Pr)_2_), 145.3 (*o*-*C*_6_H_3_(^i^Pr)_2_), 151.0 (*i*-*C*_6_H_3_(^i^Pr)_2_), 298.6 (*C*_carbene_; signal observed from 2D ^1^H–^13^C HMBC experiments). No signals could be obtained from ^29^Si{^1^H} NMR experiments. Elemental Anal. Calcd for C_41_H_75_N_3_Si_3_Ba: C, 59.21%; H, 9.09%; N, 5.05%. Found: C, 58.73%; H, 9.15%; N, 5.08%. FTIR: υ/cm^–1^ = 2942 (m), 2865 (w), 1584 (w), 1494 (w), 1485 (w), 1416 (m), 1386 (s), 1362 (w), 1335 (m), 1318 (m), 1273 (s), 1235 (s), 1200 (w), 1127 (w), 1103 (w), 1066 (s), 961 (s), 874 (s), 816 (s), 758 (s), 737 (s), 656 (s), 602 (m), 575 (m), 527 (w), 480 (m), 421 (m).

#### 4.3.8 [Ca{N(Mes)(SiMe_3_)}_2_(CAAC)] (**7**)

**1-Ca** (0.100 g, 0.22 mmol, 1 equiv) was suspended in hexane (25 mL) before CAAC (0.125 g, 0.44 mmol, 2 equiv) was added. The resulting mixture was stirred overnight at room temperature before being filtered. Slow evaporation of the filtrate at room temperature yielded **7** (0.038 g, 0.05 mmol, 23%) as colorless crystals. The NMR spectra of the crystalline material were largely uninterpretable (See Figures ESI).

#### 4.3.9 [Ca(N″)_2_(CAAC)] (**8**)

[{Ca(N″)_2_}_2_] (0.150 g, 0.42 mmol, 1 equiv) was dissolved in hexane (10 mL) before CAAC (0.237 g, 0.833 mmol, 2 equiv) was added. The resulting mixture was stirred overnight at room temperature before being filtered. Storing the filtrate at −35 °C yielded **8** (0.153 g, 0.24 mmol, 57%) as colorless crystals.

^1^H NMR (400 MHz, 298 K, C_6_D_6_): δ/ppm = 0.33 (s, 36H, Si(C*H*_3_)_3_), 0.82 (s, 6H, C(C*H*_3_)_2_), 1.06 (d, 6H, ^3^*J*_HH_ = 6.24 Hz, CH(C*H*_3_)_2_), 1.23 (s, 2H, C*H*_2_), 1.28 (d, 6H, ^3^*J*_HH_ = 6.69 Hz, CH(C*H*_3_)_2_), 1.39 (s, 6H, C(C*H*_3_)_2_), 2.61 (br m, 2H, C*H*(CH_3_)_2_), 6.97 (d, 2H, ^3^*J*_HH_ = 7.70 Hz, *m*-C_6_*H*_3_(^i^Pr)_2_), 7.09 (t, 1H, ^3^*J*_HH_ = 7.66 Hz, *p*-C_6_*H*_3_(^i^Pr)_2_). ^13^C{^1^H} (100 MHz, C_6_D_6_, 298 K): δ/ppm = 6.6 (Si(*C*H_3_)_3_), 24.0 (CH(*C*H_3_)_2_), 28.4 (C(*C*H_3_)_2_), 28.8 (CH(*C*H_3_)_2_), 29.1 (C(*C*H_3_)_2_), 50.4 (*C*H_2_), 57.0 (*C*(CH_3_)_2_), 83.2 (*C*(CH_3_)_2_), 125.3 (*m*-*C*_6_H_3_(^i^Pr)_2_), 129.5 (*p*-*C*_6_H_3_(^i^Pr)_2_), 135.8 (*i*-*C*_6_H_3_(^i^Pr)_2_), 145.1 (*o*-*C*_6_H_3_(^i^Pr)_2_), 277.2 (*C*_Carbene_; signal observed only from 2D ^1^H–^13^C HMBC experiments). ^29^Si{^1^H} NMR (80 MHz, 298 K, C_6_D_6_): δ/ppm = −14.23. Elemental Anal. Calcd for C_32_H_67_CaN_3_Si_4_: C, 59.47%; H, 10.45%; N, 6.50%. Found: C, 59.64%; H, 10.50%; N, 6.20%. FTIR: υ/cm^–1^ = 2955 (s), 2926 (s), 2864 (m), 1446 (m), 1382 (m), 1367 (w), 1335 (m), 1323 (m), 1260 (w), 1238 (w), 1206 (m), 1176 (w), 1144 (m), 1050 (w), 975 (w), 957(w), 940 (s), 898 (m), 876 (w), 815 (s), 776 (s), 763 (w), 703 (w), 573 (w), 535 (w), 473 (m), 445 (m).

### 4.4 Computational Method

Input coordinates for all **3-AE**, **4-AE**, **7**, and **8** were taken from crystal structures. The coordinates for putative [AE(L)_2_(CAAC)]^−^ (L = N″, {N(Mes)(SiMe_3_)}^−^, {N(Mes)(SiMe_3_)}^−^) were obtained by optimizing to a minimum the structures of **3-AE**, **4-AE**, **7**, and **8**, followed altering the charge and multiplicity, and optimizing to a minimum the structure once again. Countercations were completely excluded from the calculations.

DFT calculations were run with Gaussian 09 (Revision E.01) using the B3PW91 functional.^54^ For geometry optimizations, the Ca–Ba centers were described with the Stuttgart ECPs and associated basis sets,^55^ and 6-31G basis sets were used for H, C, and N, and 6-31G(d) for Si (defined as BS1).^56,57^ Single point energy and NBO^58^ calculations used the same basis sets for Ca–Ba, 6-311+G** for H, C, and N, and 6-311+G(3d) for Si (BS2).^56,57^ All stationary points being fully characterized via analytical frequency calculations as minima. Molecular orbital images were made using GaussView 6.1.^59^

## Data Availability

Additional research data supporting this publication are available from Figshare at 10.25392/leicester.data.26660914.v1.

## Supporting Information Available

Additional experimental details and spectroscopic data (multinuclear NMR, IR), crystallographic data for all compounds, with additional computational details (DFT) (PDF)
