# Supplementary material for: Heavy Alkaline Earth Cyclic (Alkyl)(Amino)Carbene Complexes Supported by Aryl–Silyl Amides
Source: Inorg Chem. 2024 Nov 6;63(46):22061–73. doi: 10.1021/acs.inorgchem.4c03494 (PMC11577317; doi:10.1021/acs.inorgchem.4c03494)
Supplement: Supplementary file 1 — ic4c03494_si_001.pdf [file ic4c03494_si_001.pdf]

Heavy Alkaline Earth Cyclic (Alkyl)(Amino)Carbene Complexes Supported by Aryl-silyl Amides

Alex W. J. Bowles,[a,§] Yu Liu,[a,§] Matthew P. Stevens,[a,b] Fabrizio Ortu*[a]

[a]School of Chemistry, University of Leicester, University Road, Leicester, LE1 7RH, UK

[b]Department of Chemistry, University of Bath, Claverton Down, Bath, BA2 7AY, UK

*email: fabrizio.ortu@leicester.ac.uk

[§]These authors all contributed equally

Table of Contents

1. NMR data S2
2. IR data S17
3. Crystallography S28
4. Computational details S34
5. References S59

1. NMR data

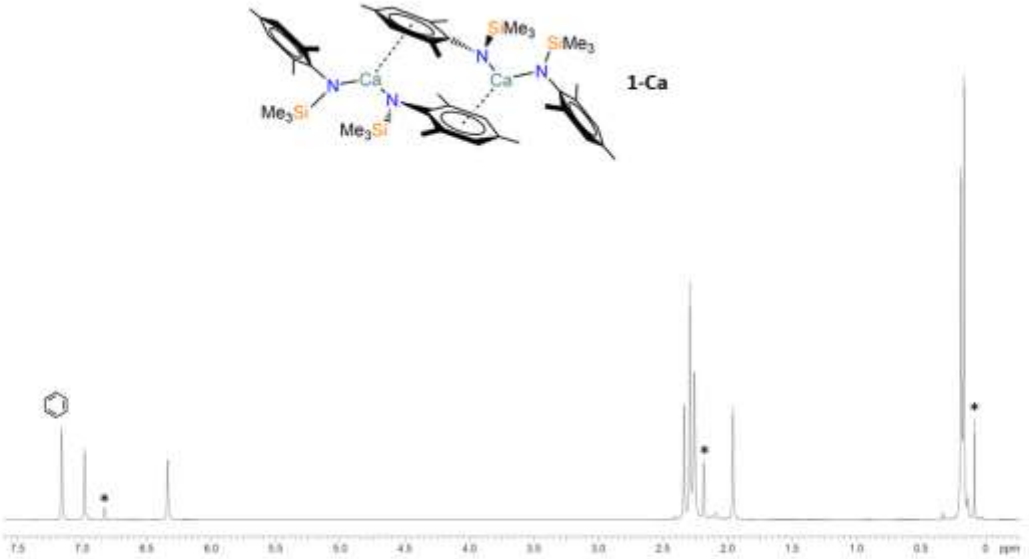

Figure S1: ^{1}H NMR spectrum of **1-Ca**; signals assigned to known [Ca(NMes)$_3$K] impurity denoted by *.

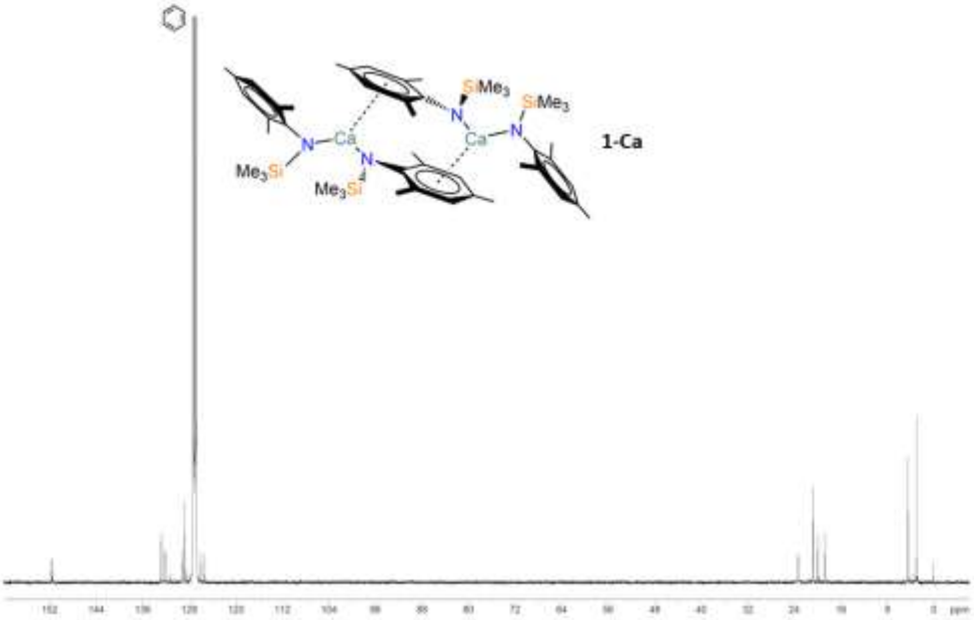

Figure S2: ^{13}C{^{1}H} NMR spectrum of **1-Ca**.

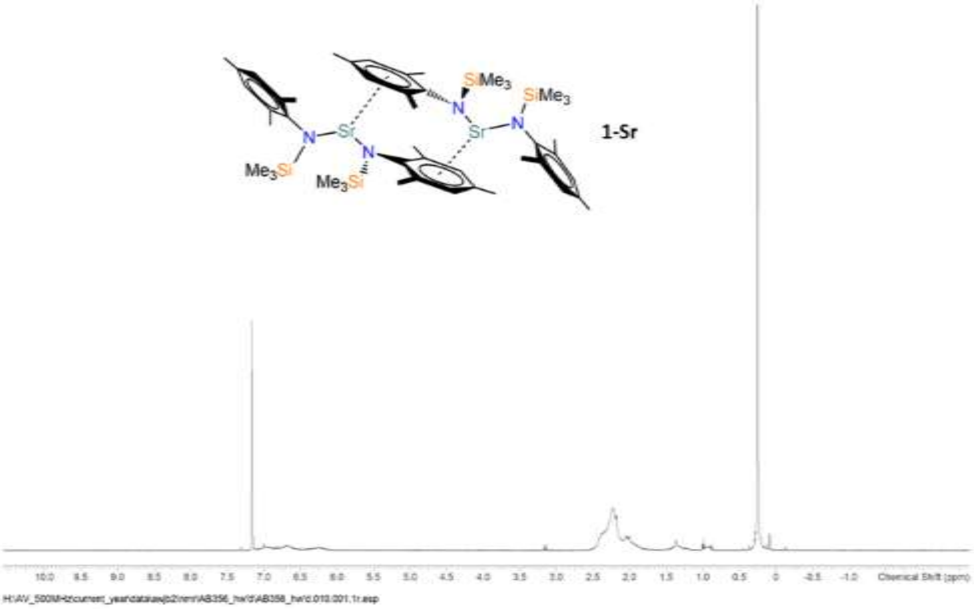

Figure S3: ^{1}H NMR spectrum of **1-Sr** with assignments.

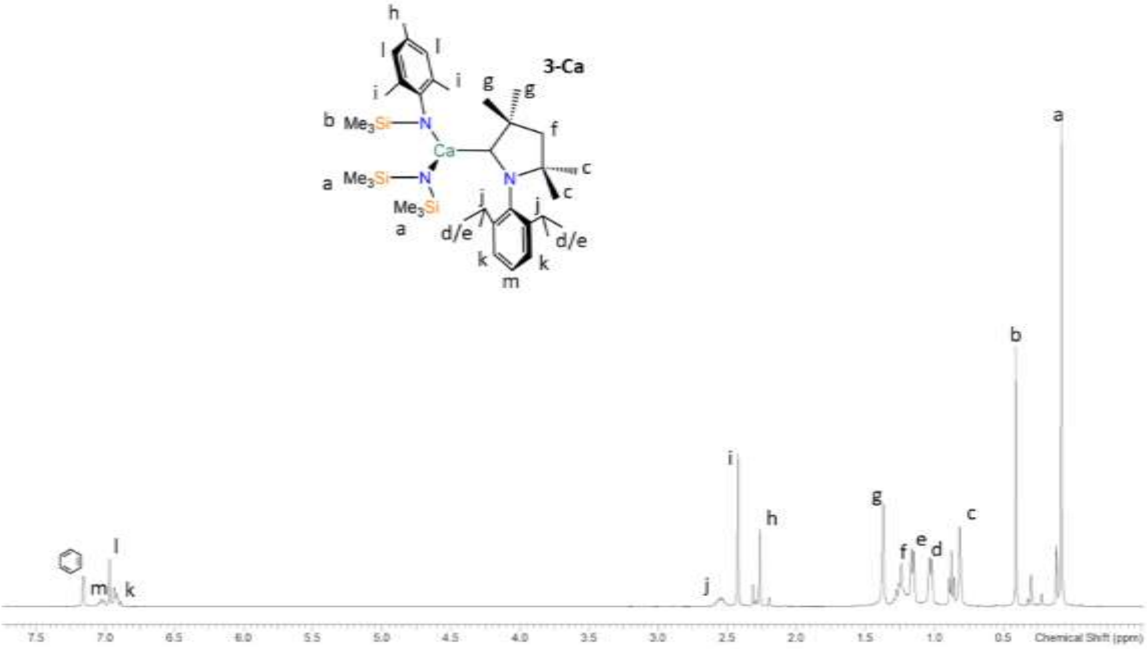

Figure S4: ^{1}H NMR spectrum of **3-Ca** with assignments.

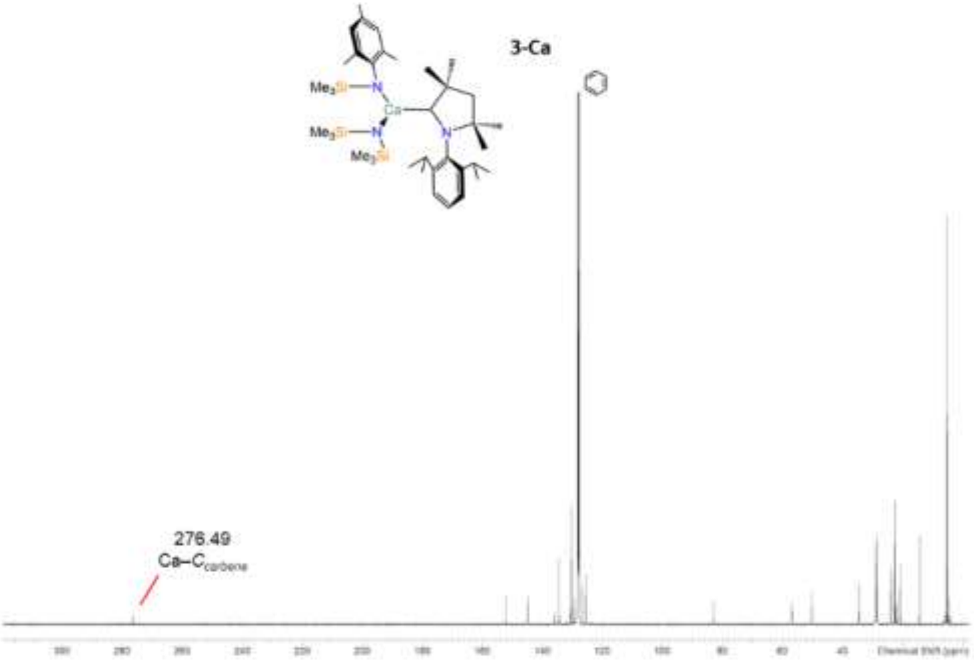

Figure S5: $^{13}C\{^{1}H\}$ NMR spectrum of **3-Ca** with carbene signal labelled.

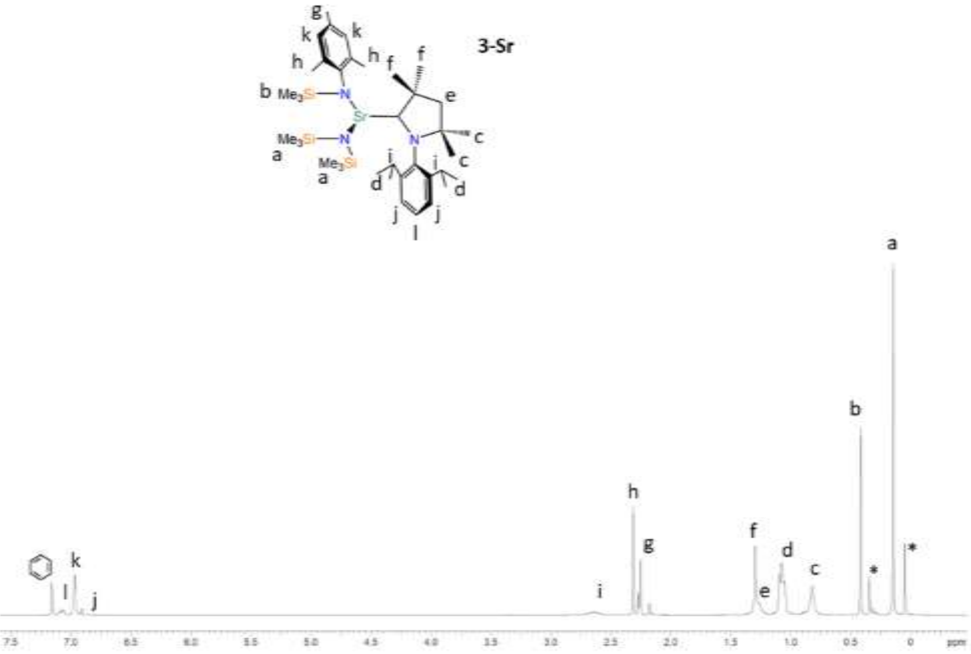

Figure S6: ^{1}H NMR spectrum of **3-Sr** with assignments. Signals corresponding to [{Sr(NMes)(μ-N″)}$_2$] denoted with *.

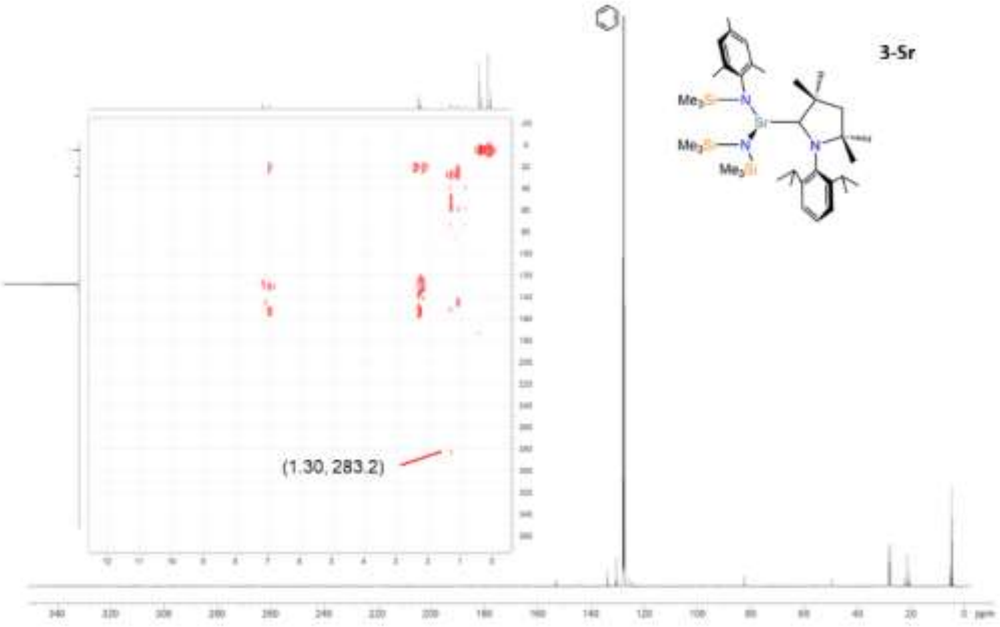

Figure S7: $^{13}C\{^1H\}$and ^1H-^{13}C HMBC NMR spectra of **3-Sr** with carbene signal labelled.

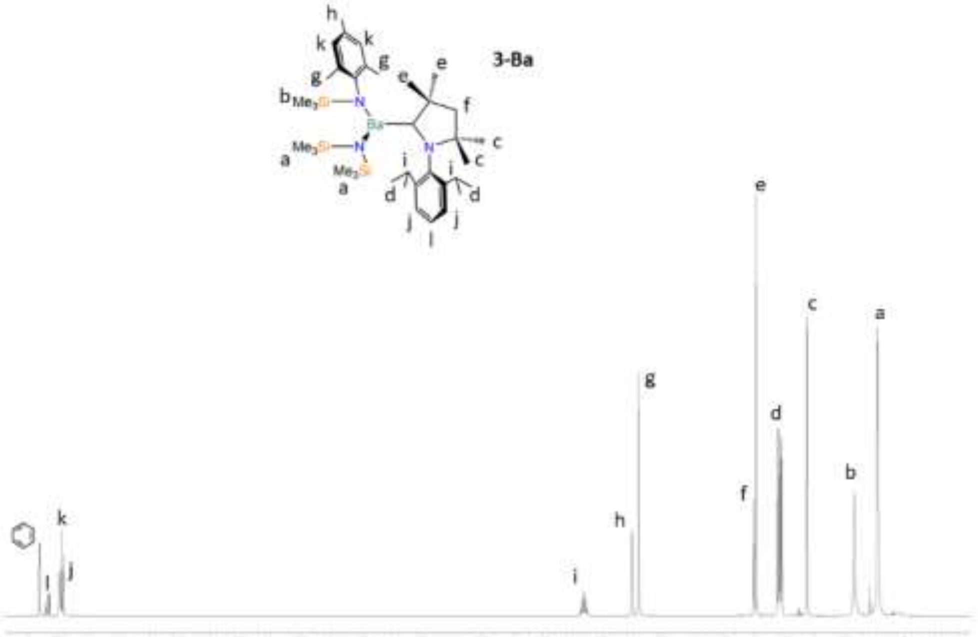

Figure S8: ^1H NMR spectrum of **3-Ba** with assignments.

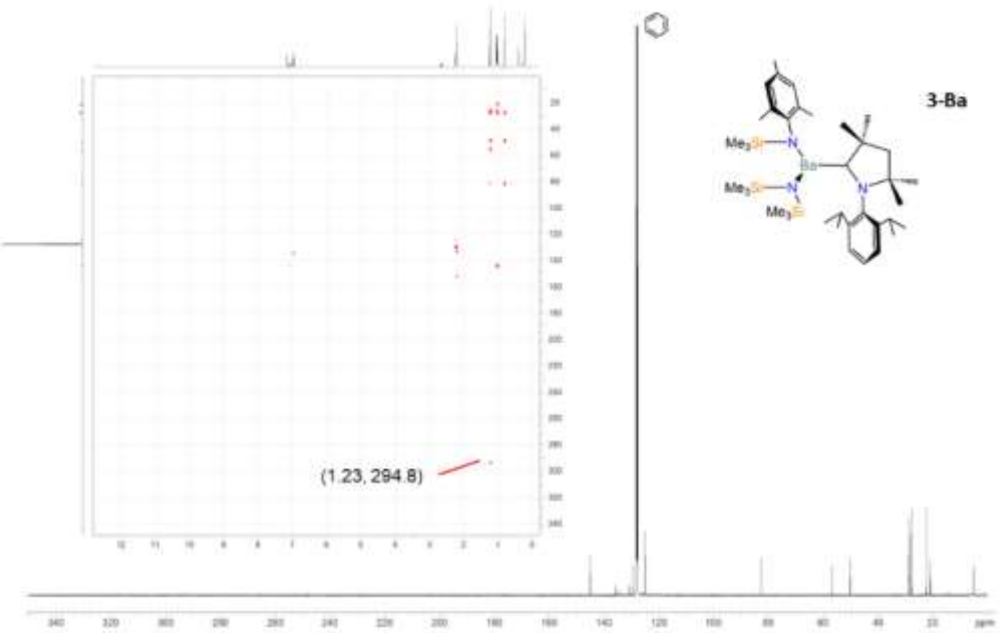

Figure S9: $^{13}C\{^{1}H\}$ and ^{1}H-^{13}C HMBC NMR spectra of **3-Ba** with carbene signal labelled.

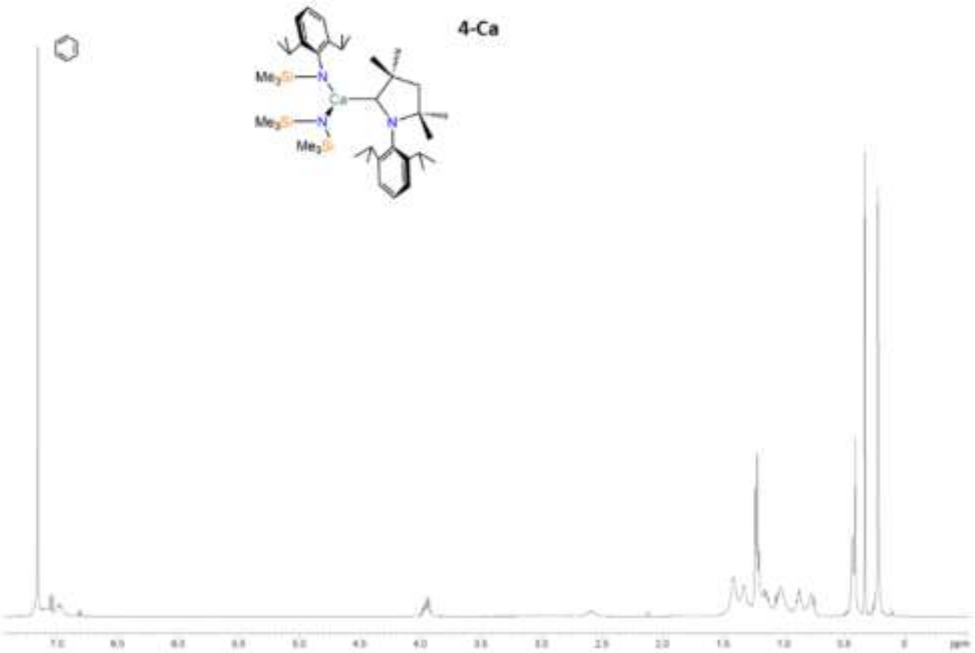

Figure S10: ^{1}H NMR spectrum of **4-Ca**.

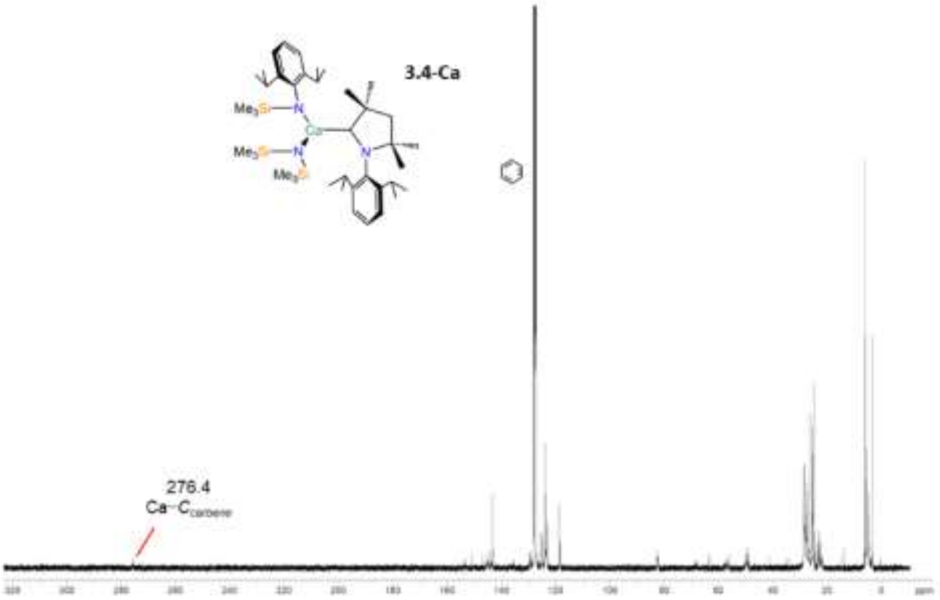

Figure S11: $^{13}C\{^{1}H\}$ NMR spectrum of **4-Ca** with carbene signal labelled.

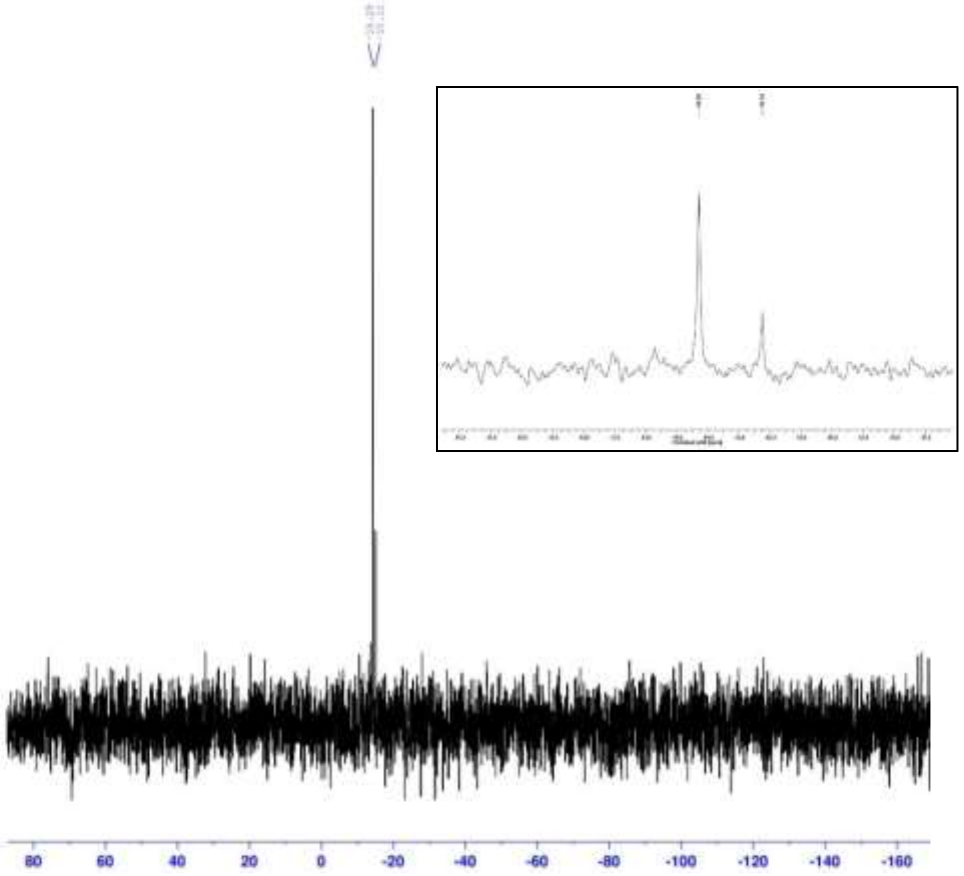

Figure S12: $^{29}Si\{^{1}H\}$ NMR spectrum of **4-Ca** and inset showing the region between –11 and –17 ppm.

.

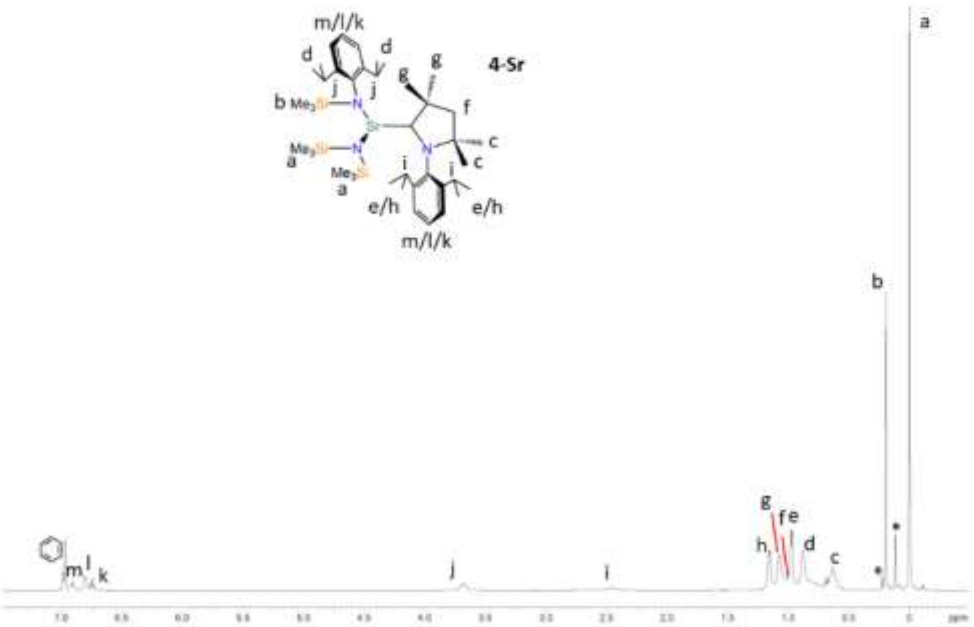

Figure S13: ^{1}H NMR spectrum of **4-Sr** with assignments. Signals corresponding to the suspected impurity [{Sr(NDipp)(μ-N″)}$_2$] denoted with *.

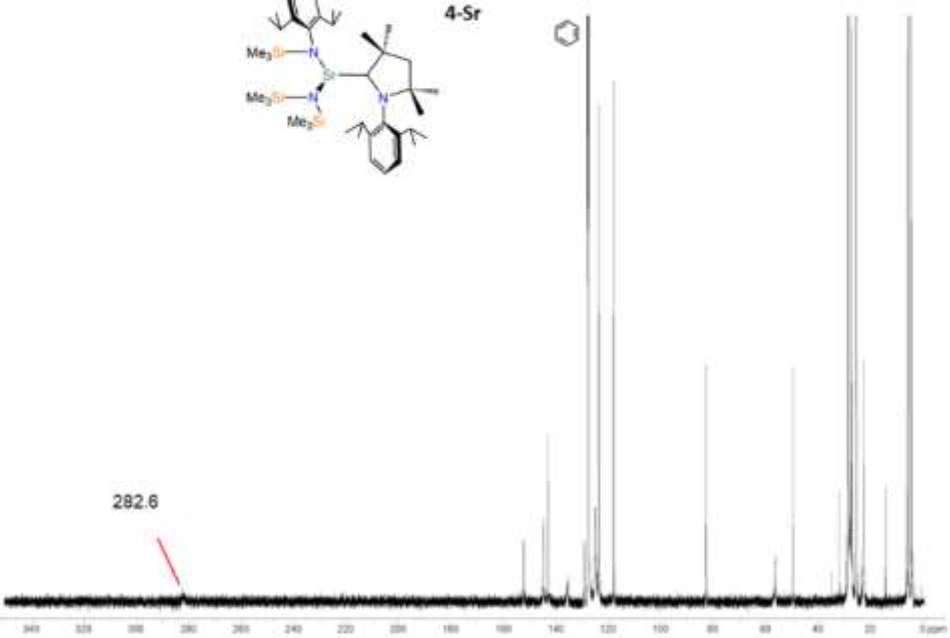

Figure S14: $^{13}C\{^{1}H\}$ NMR spectrum of **4-Sr** with carbene signal labelled.

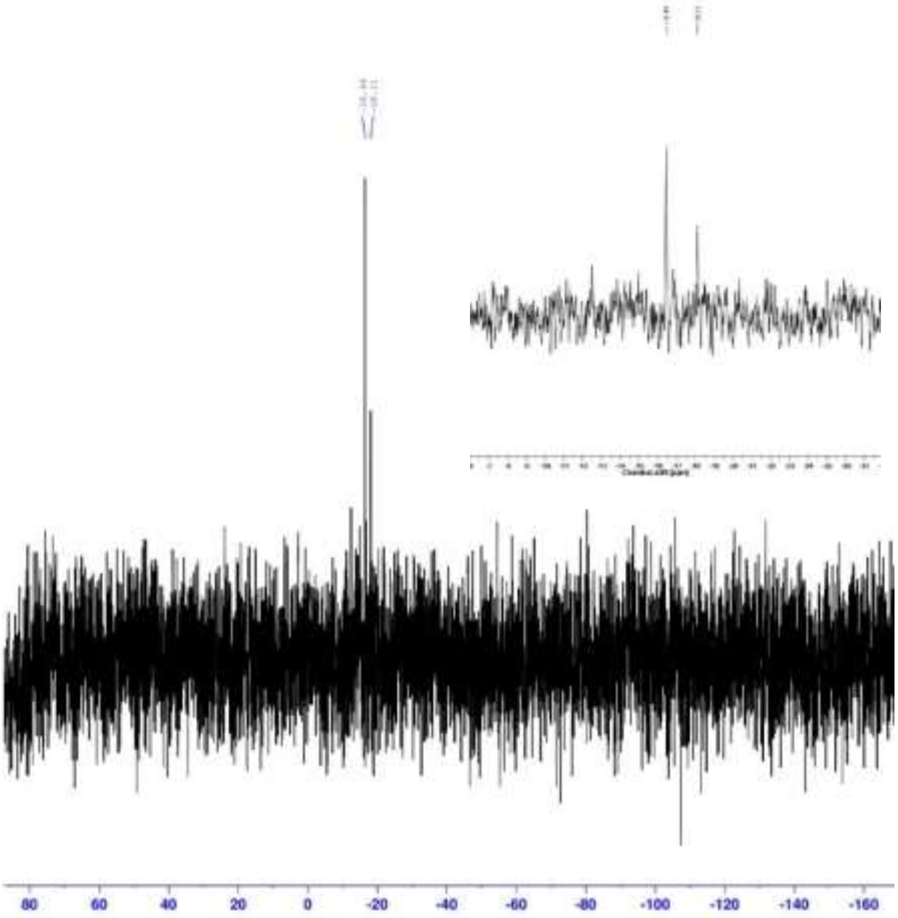

Figure S15: $^{29}Si\{^{1}H\}$ NMR spectrum of **4-Sr** and inset showing the region between –7 and –26 ppm.

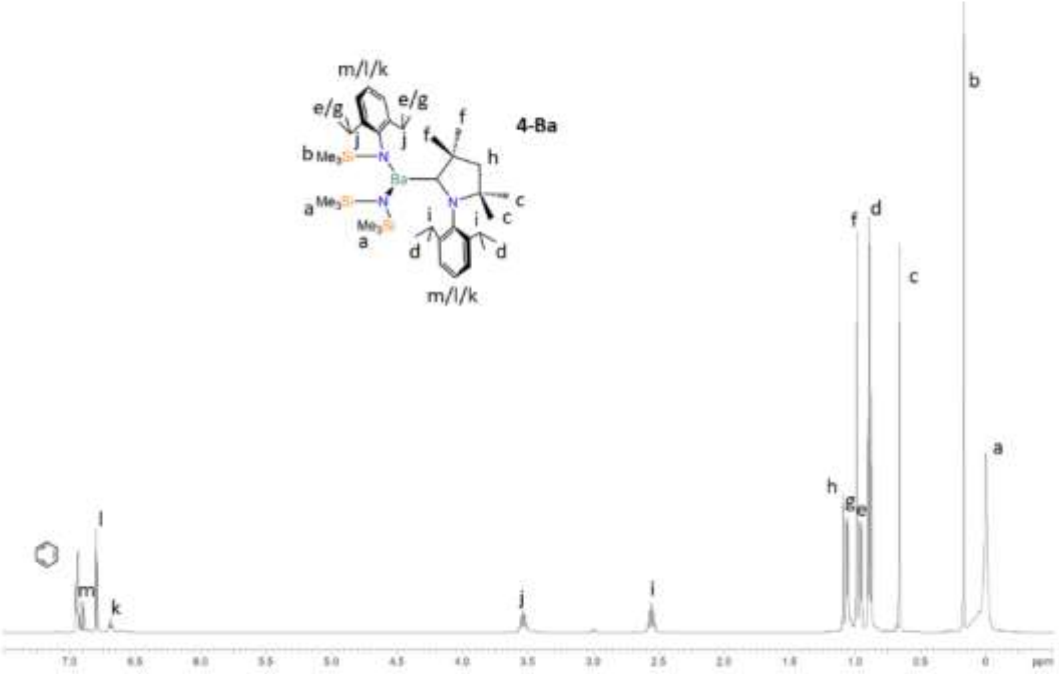

Figure S16: ^{1}H NMR spectrum of **4-Ba** with assignments.

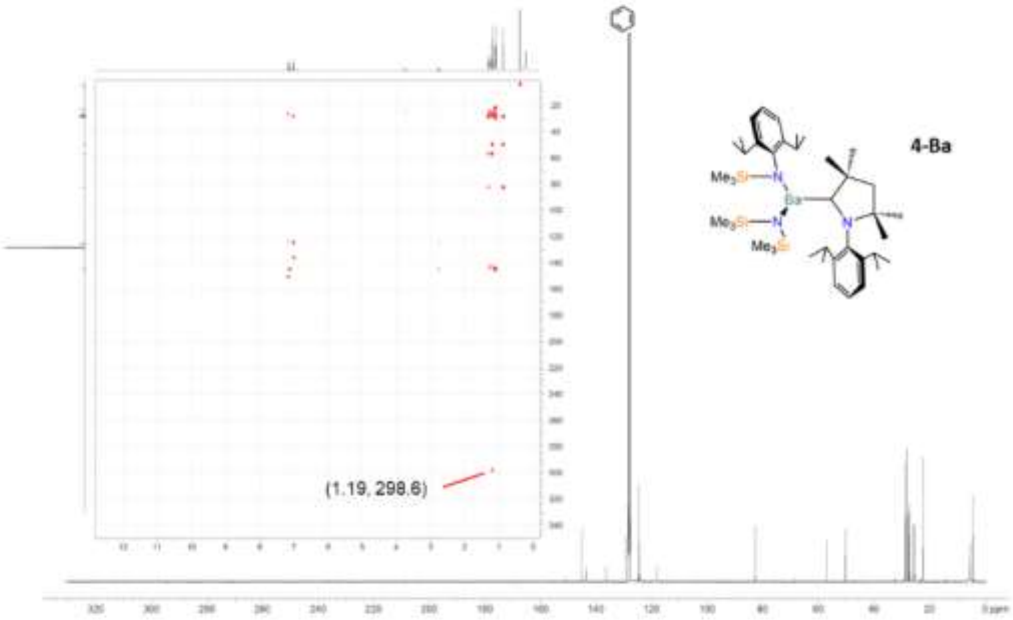

Figure S17: $^{13}C\{^{1}H\}$ and ^{1}H-^{13}C HMBC NMR spectra of **4-Ba** with carbene signal labelled.

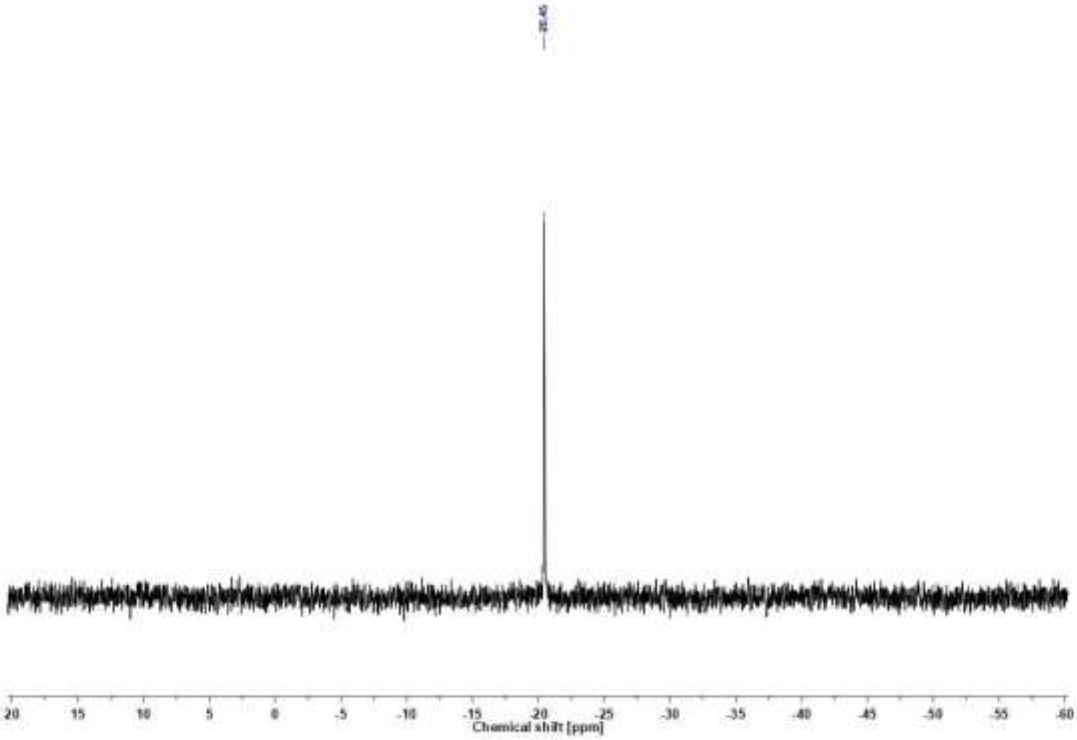

Figure S18: $^{29}Si\{^{1}H\}$ NMR spectrum of **4-Ba.**

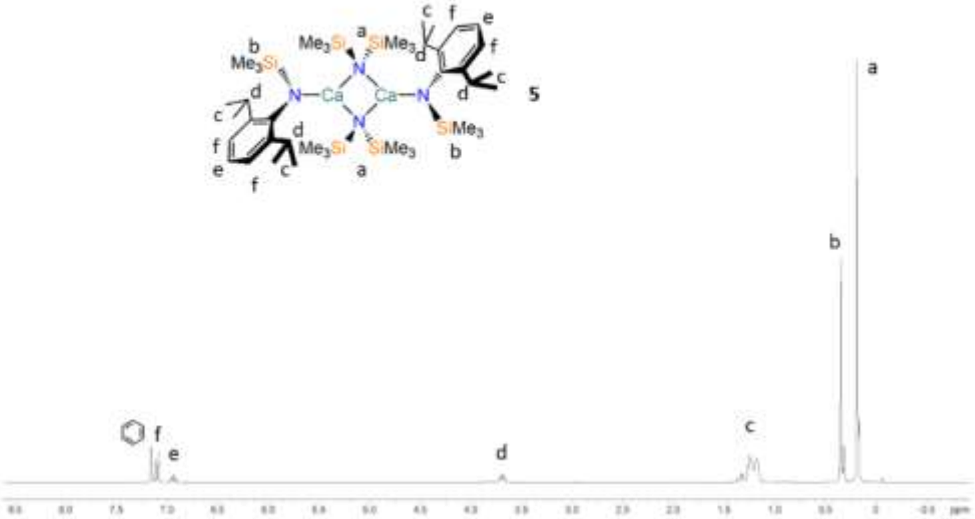

Figure S19: ^{1}H NMR spectrum of **5** with assignments.

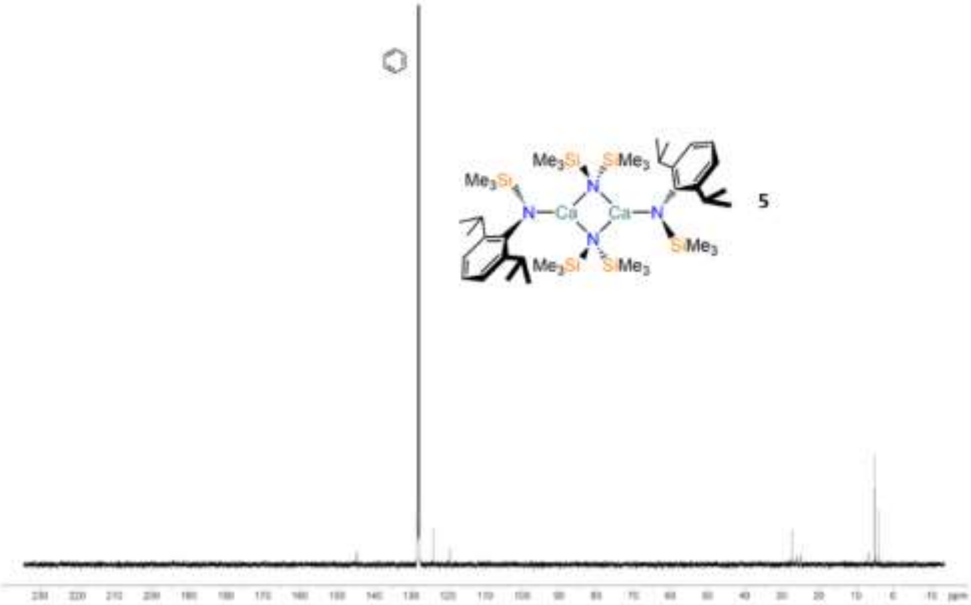

Figure S20: $^{13}C\{^{1}H\}$ spectrum of **5**.

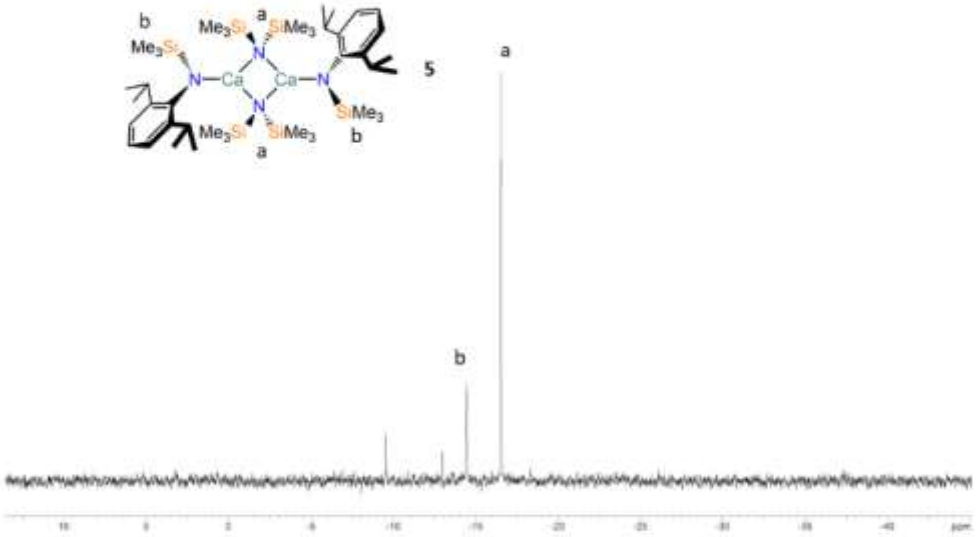

Figure S21: $^{29}Si\{^{1}H\}$ NMR spectrum of **5** with assignments.

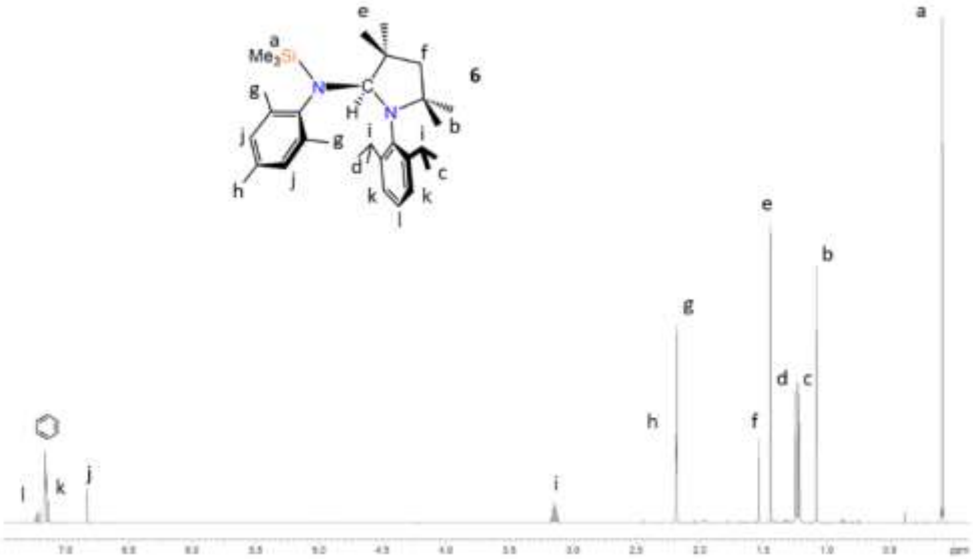

Figure S22: ^{1}H NMR spectrum of **6** with assignments.

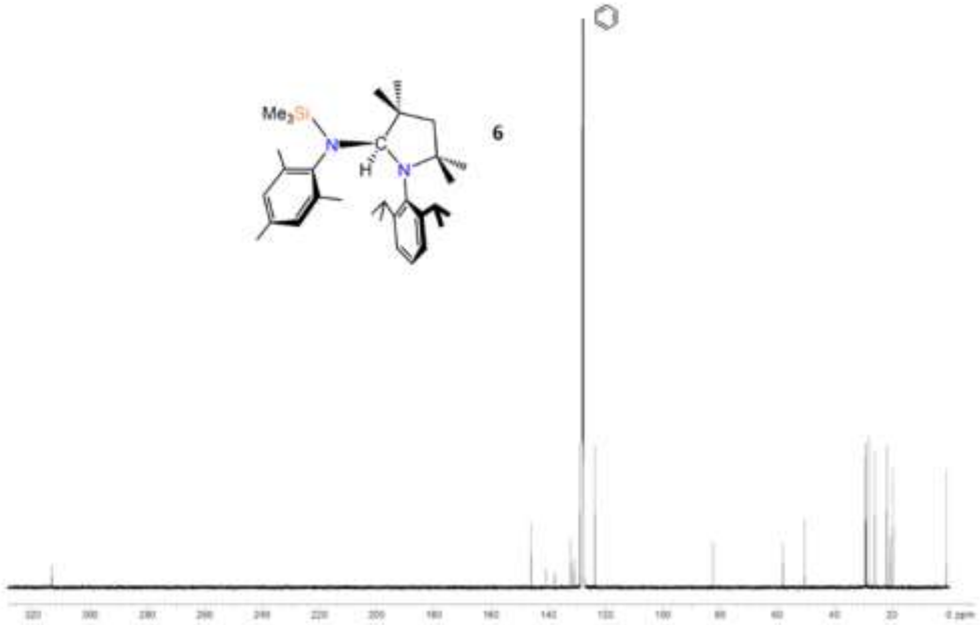

Figure S23: $^{13}C\{^{1}H\}$ NMR spectrum of **6**.

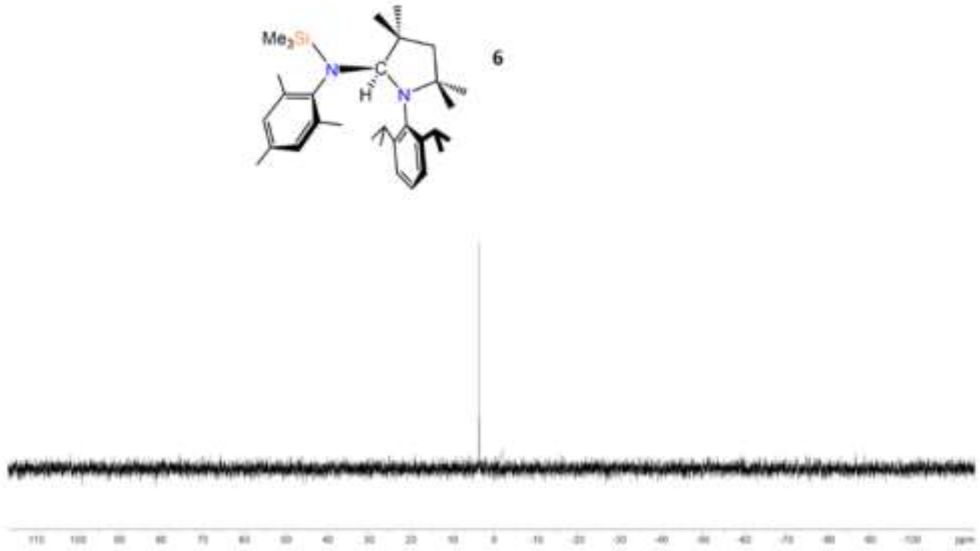

Figure S24: $^{29}Si\{^{1}H\}$ NMR spectrum of **6**.

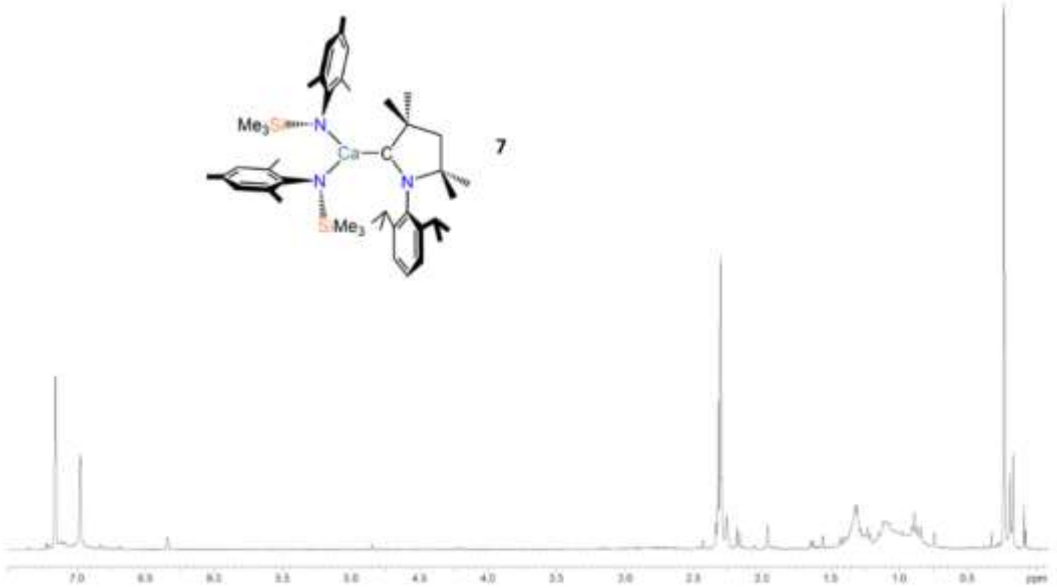

Figure S25: ^{1}H NMR spectrum of **7**.

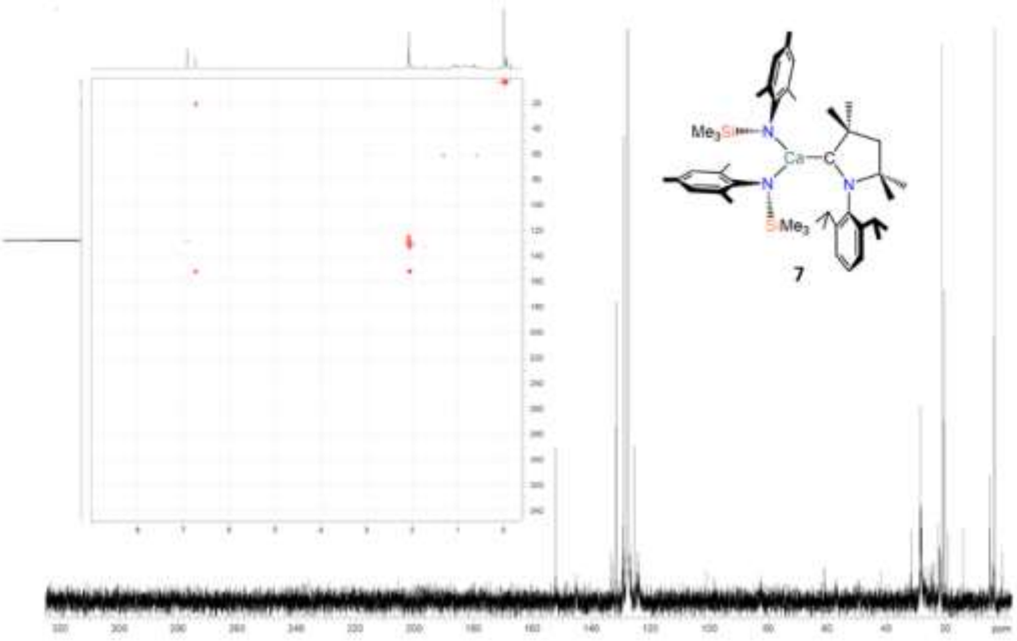

Figure S26: ^{13}C{^{1}H} NMR spectrum of **7**.

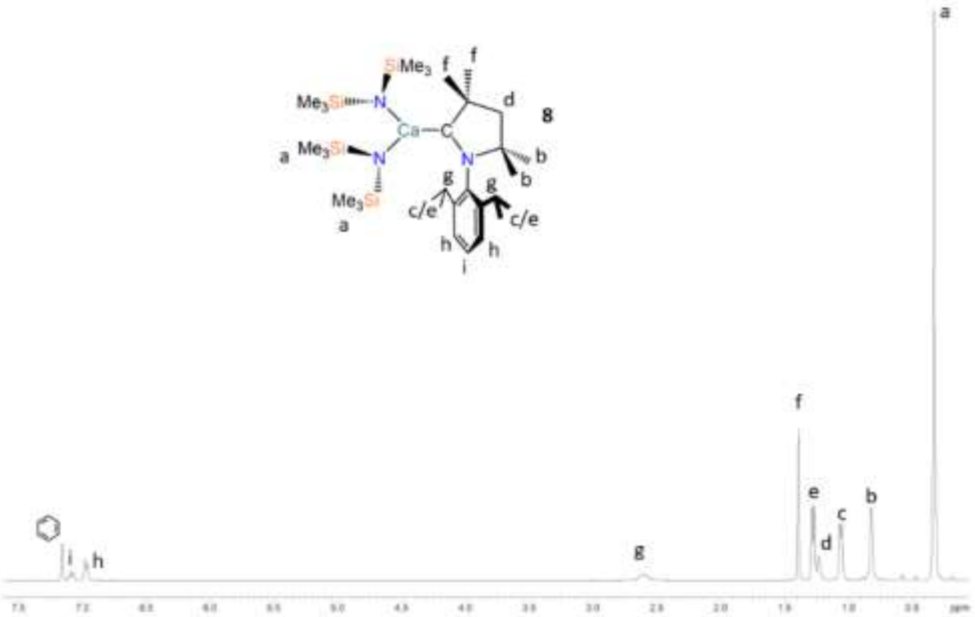

Figure S27: ^{1}H NMR spectrum of **8** with assignments.

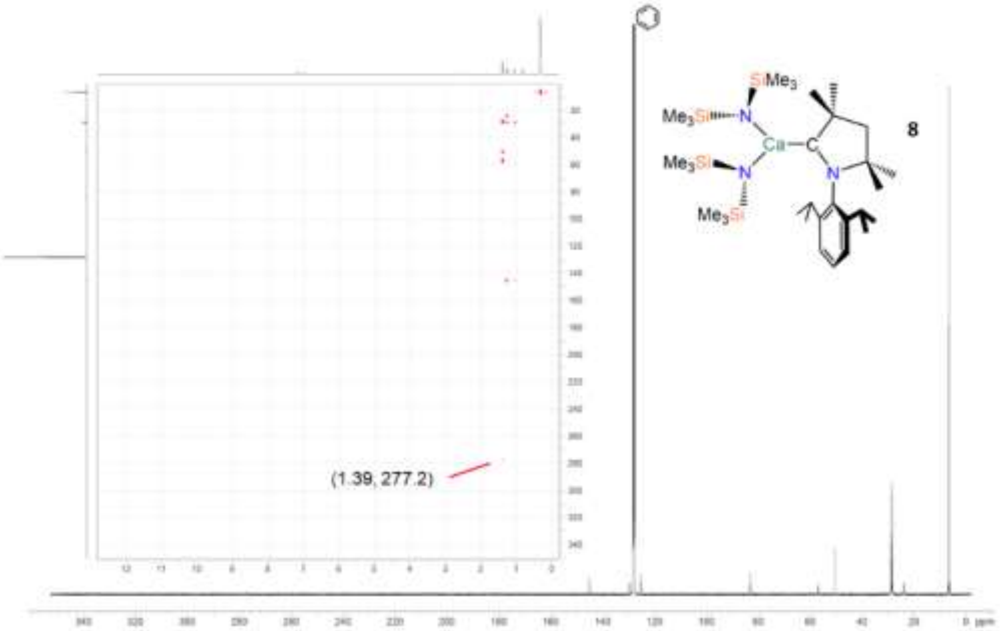

Figure S28: ^{13}C{^{1}H} and ^{1}H-^{13}C HMBC NMR spectra of **8** with the carbene signal labelled.

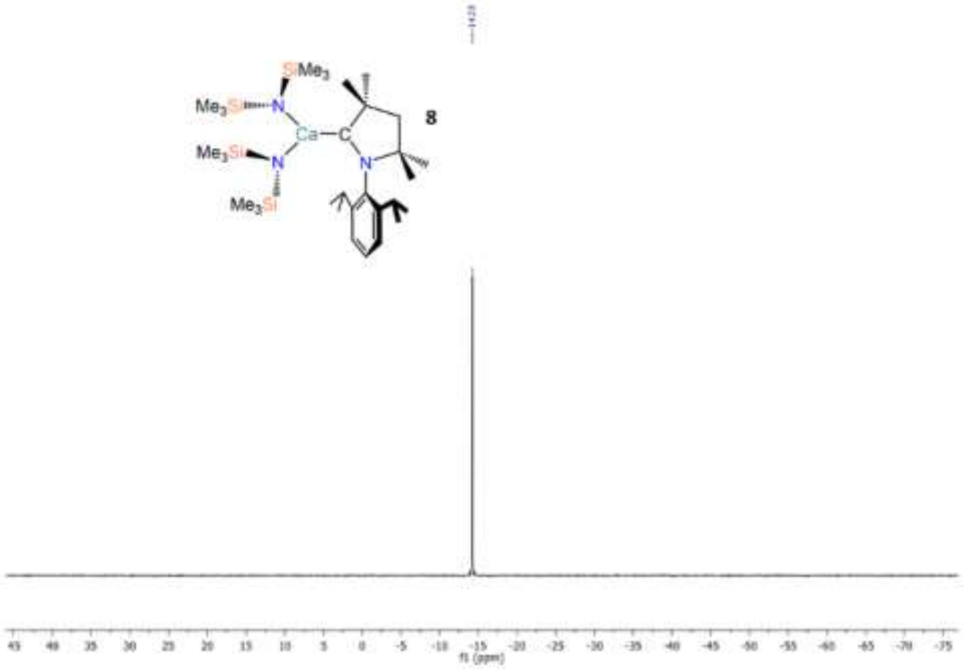

Figure S29: ^{29}Si{^{1}H} NMR spectrum of **8**.

2. IR data

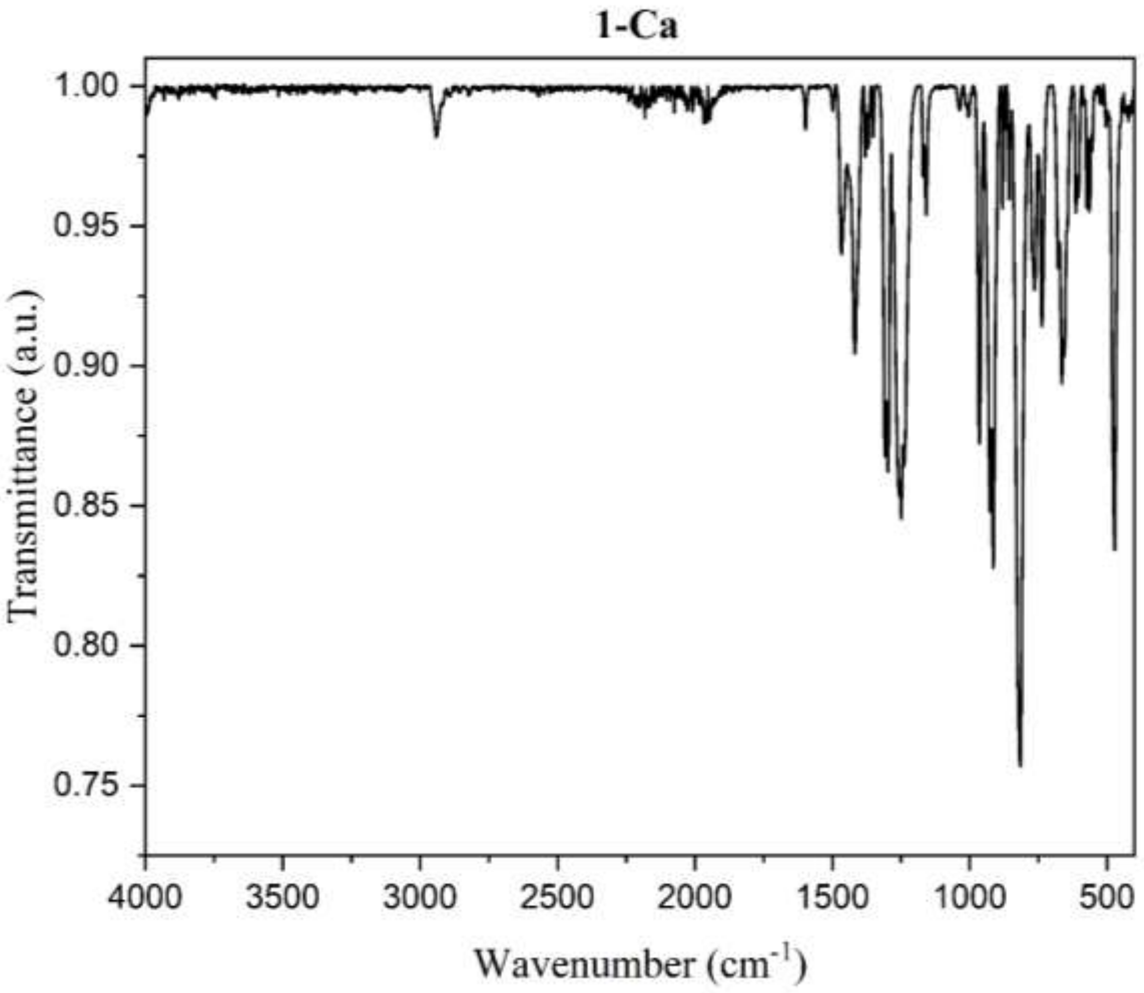

Figure S30: FTIR spectroscopy of **1-Ca**.

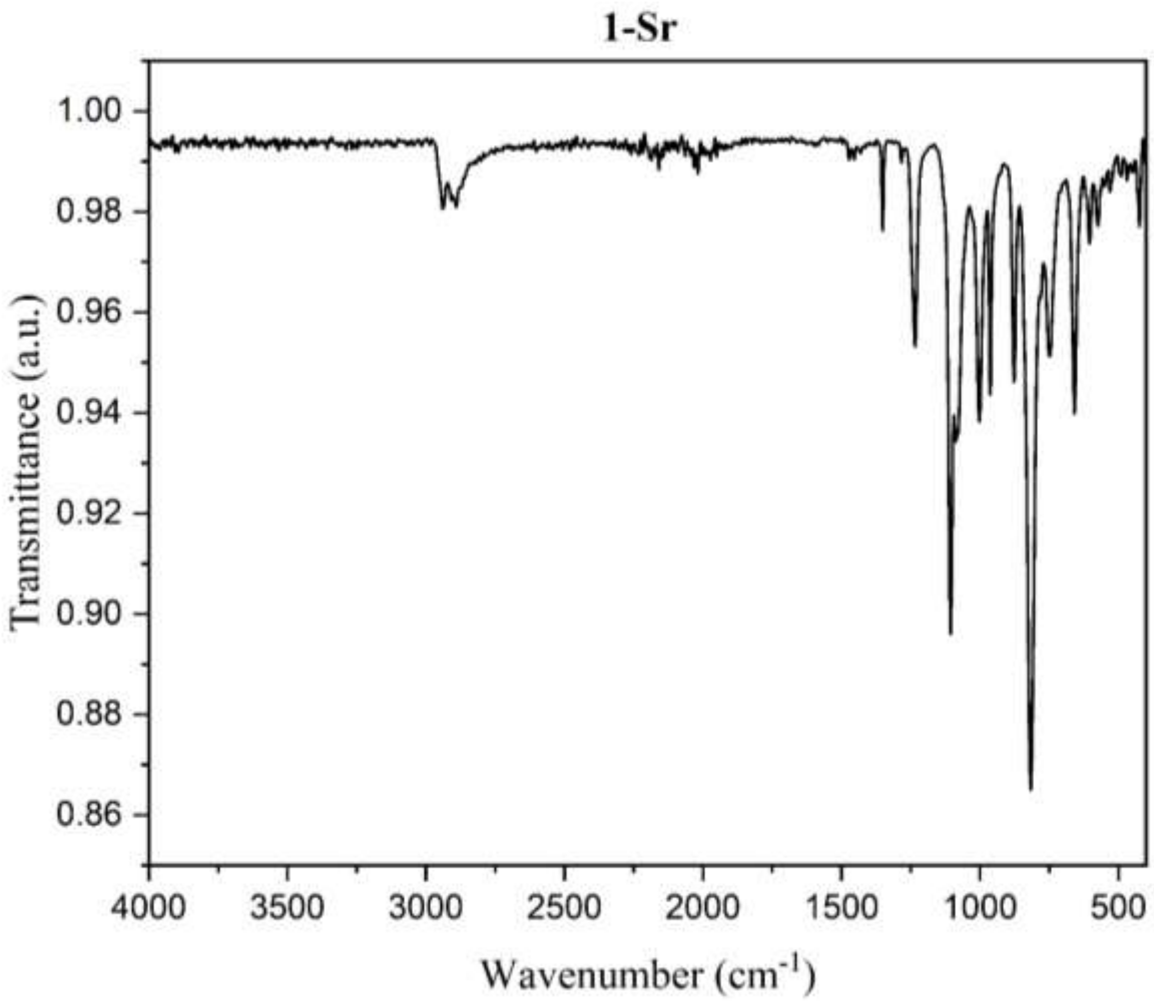

Figure S31: FTIR spectroscopy of **1-Sr**.

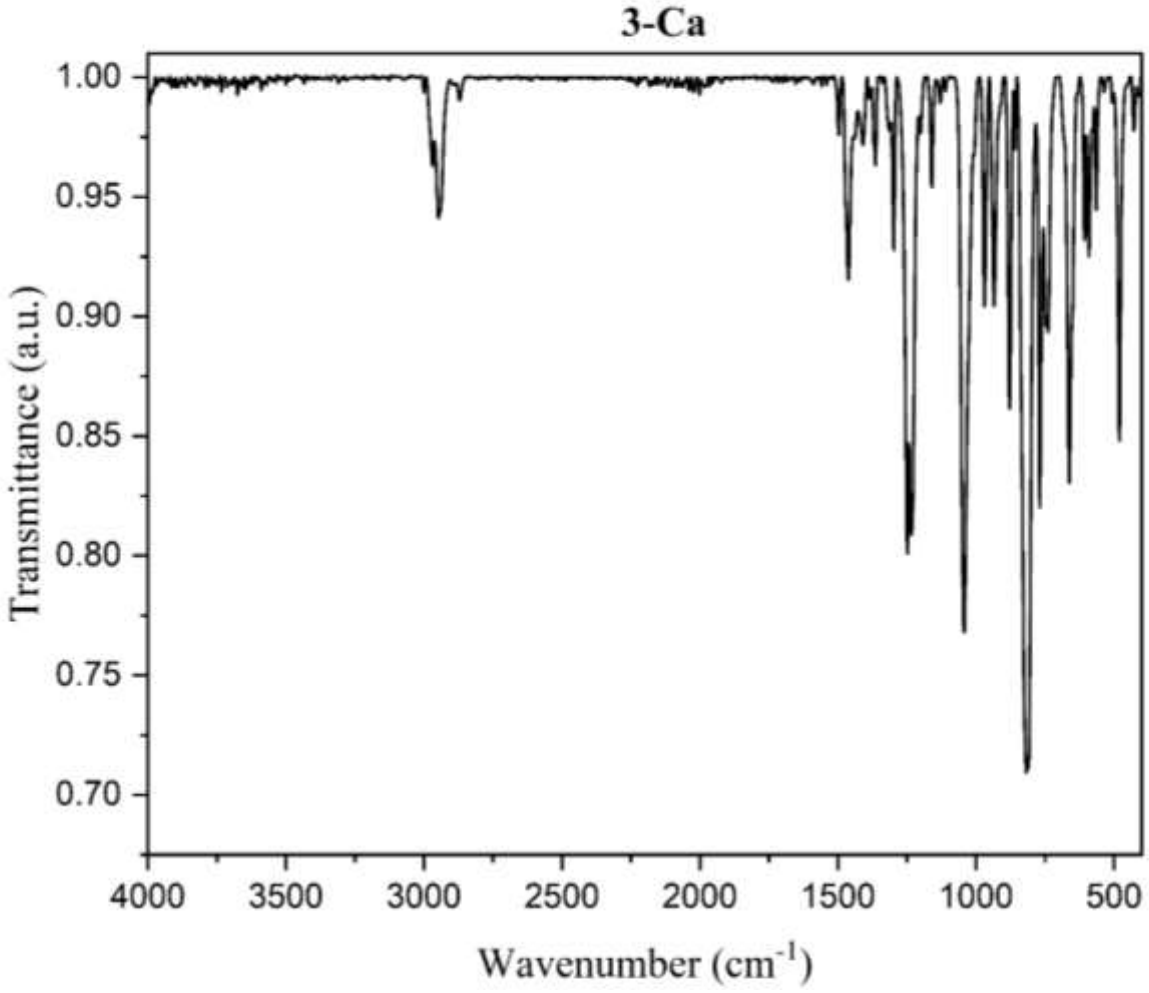

Figure S32: FTIR spectrum of **3-Ca**.

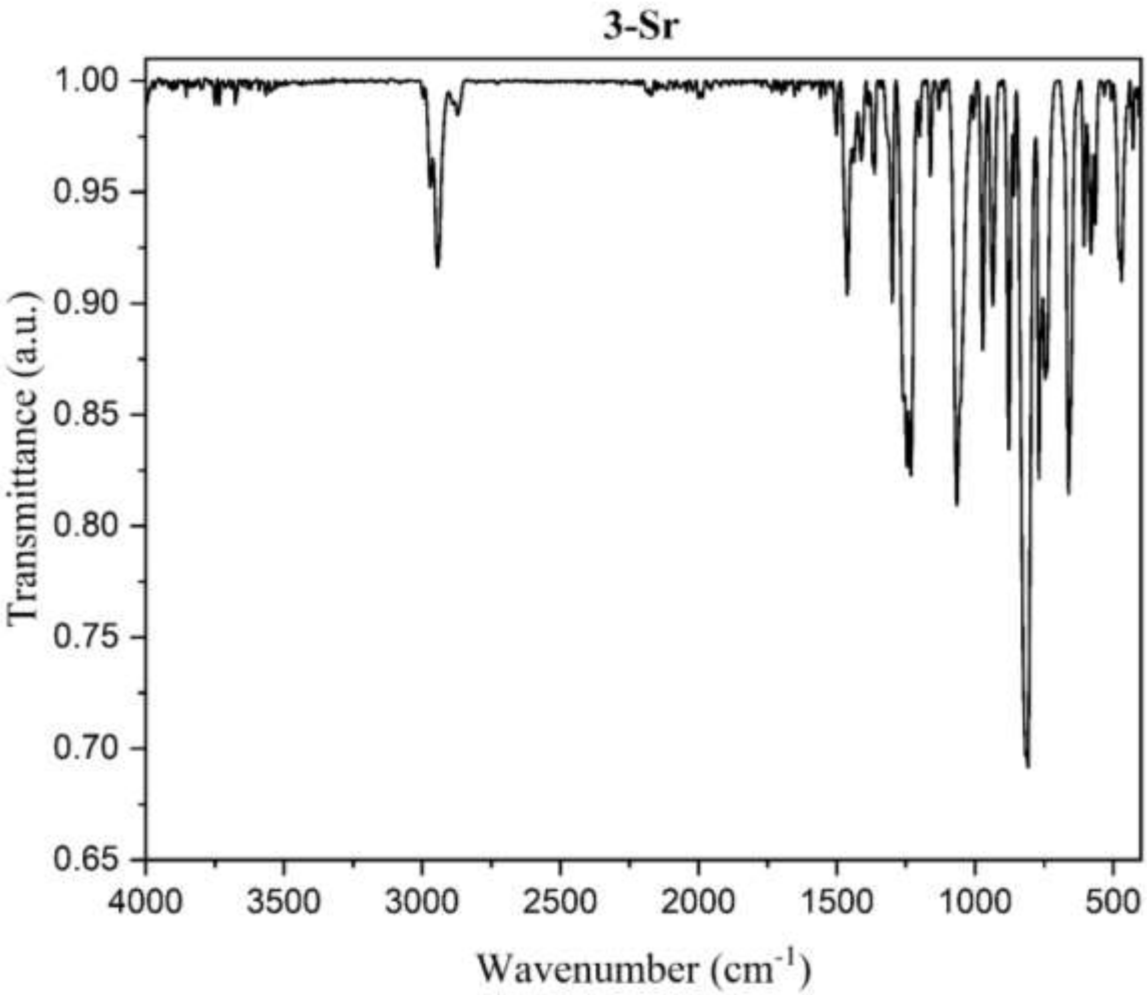

Figure S33: FTIR spectrum of **3-Sr**.

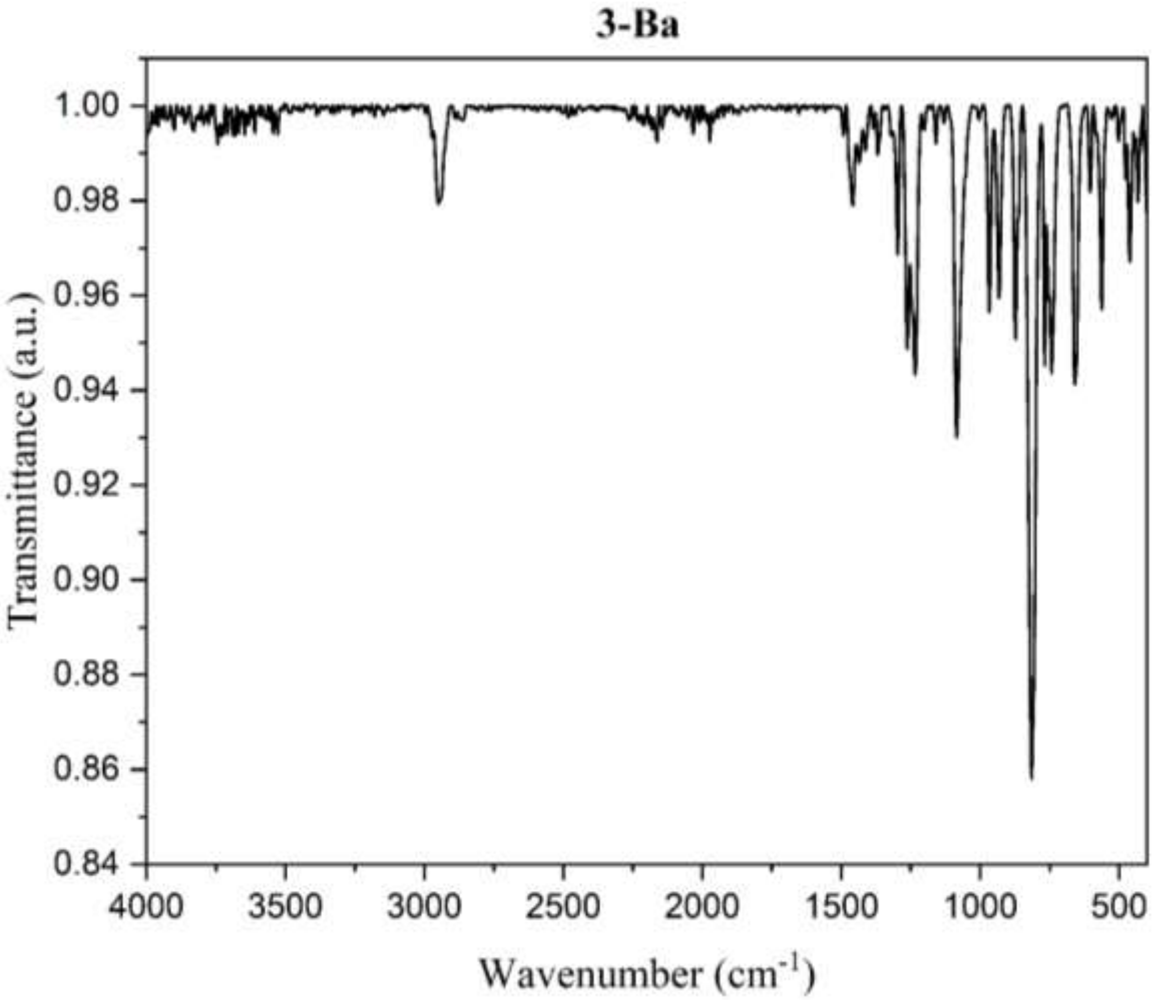

Figure S34: FTIR spectrum of **3-Ba**.

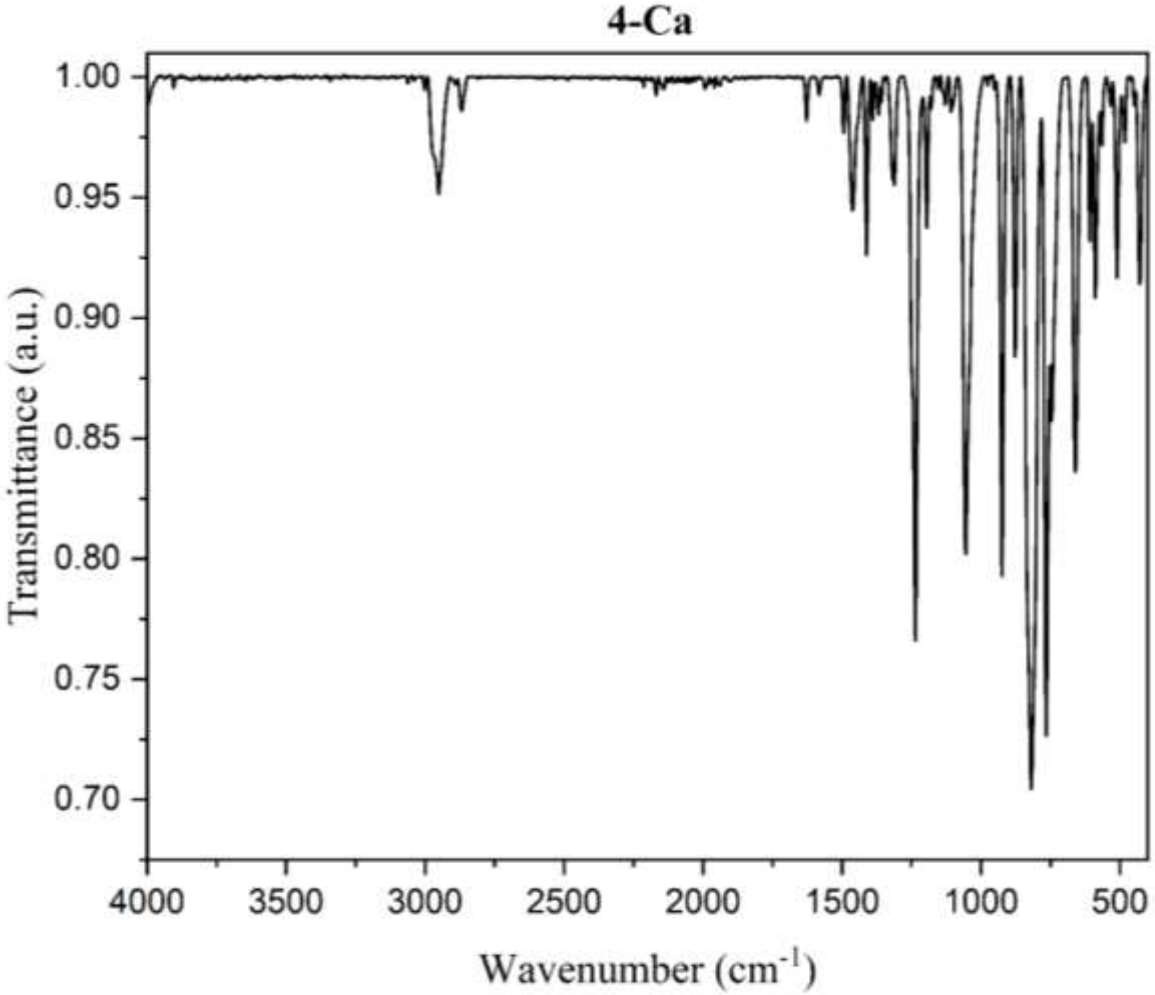

Figure S35: FTIR spectrum of **4-Ca**.

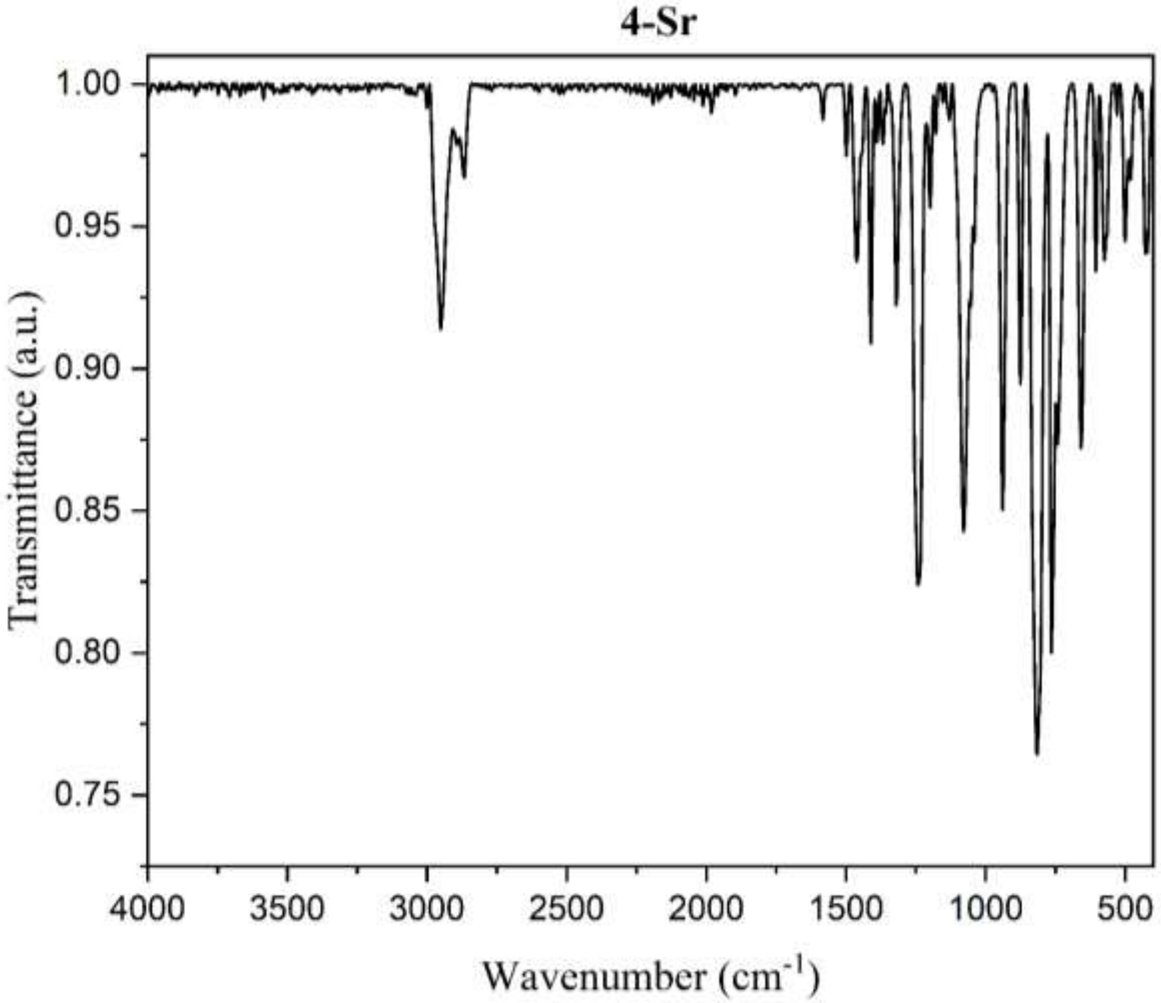

Figure S36: FTIR spectrum of **4-Sr**.

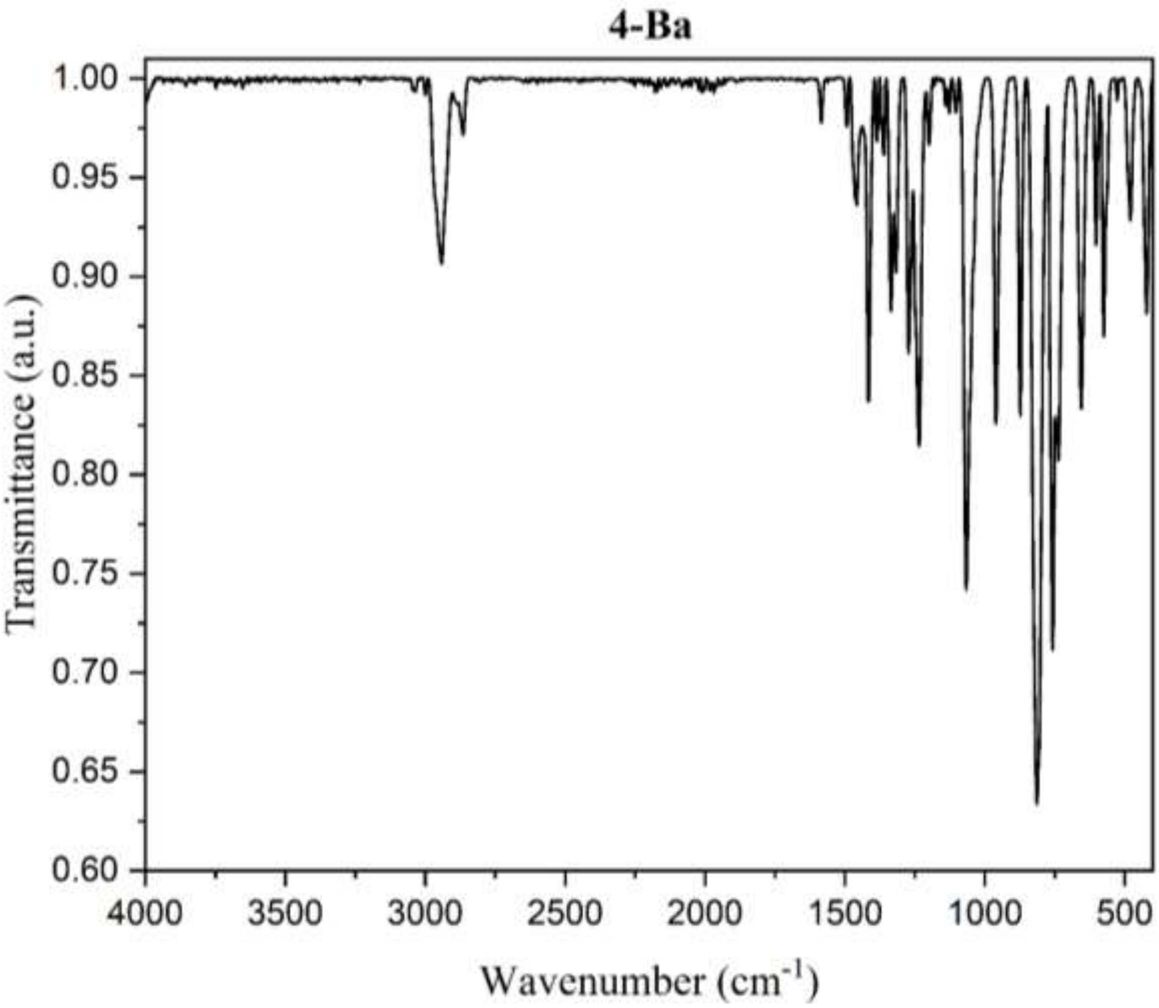

Figure S37: FTIR spectrum of **4-Ba**.

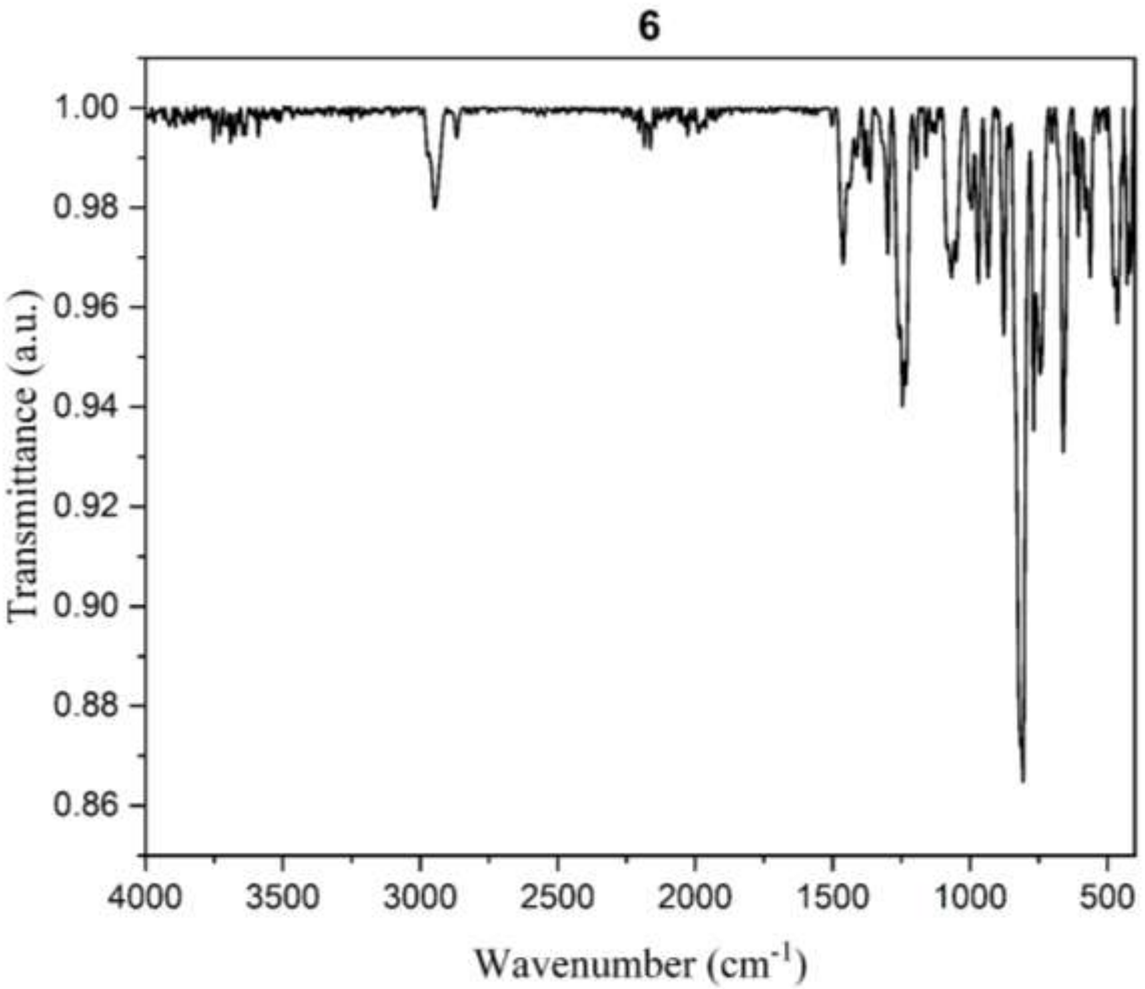

Figure S38: FTIR spectrum of **6**.

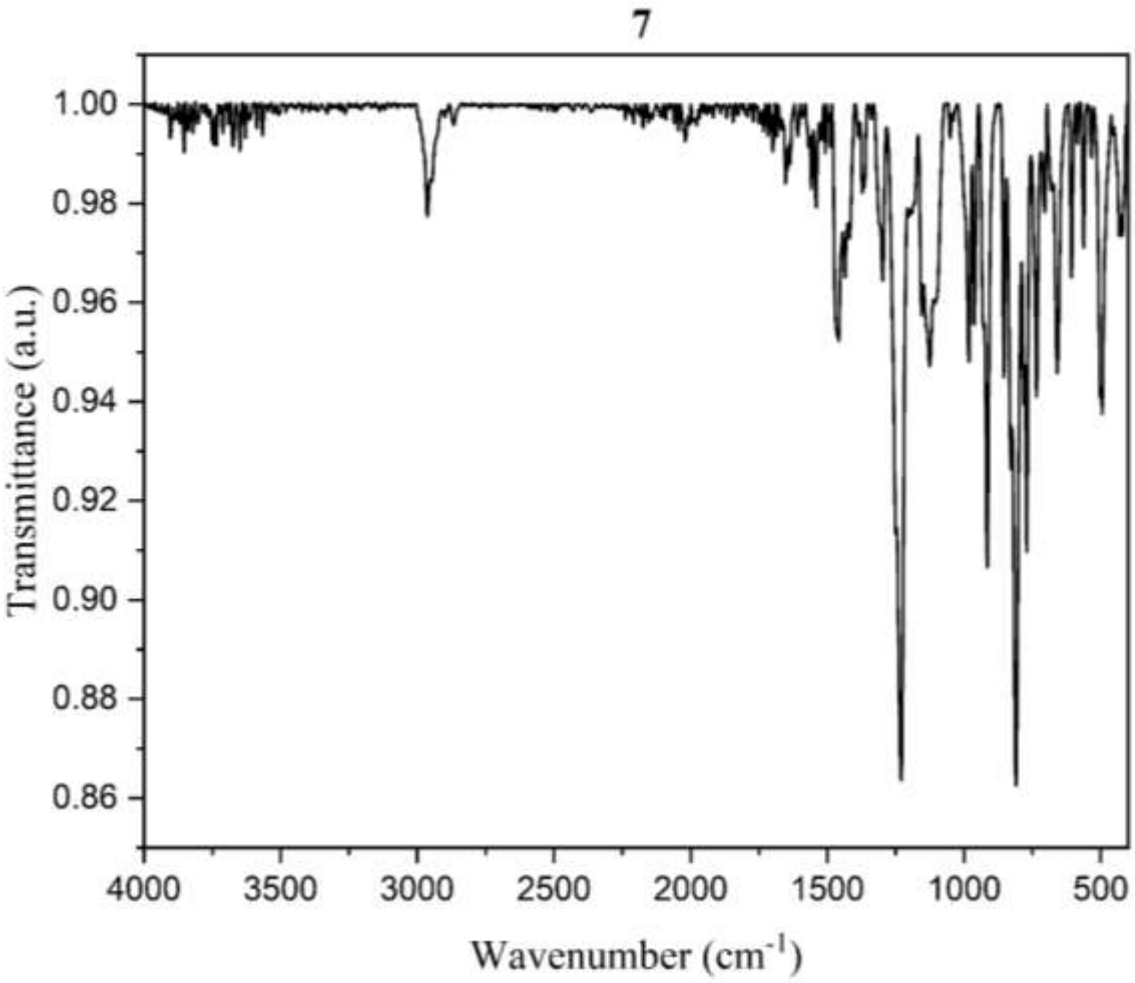

Figure S39: FTIR spectrum of **7**.

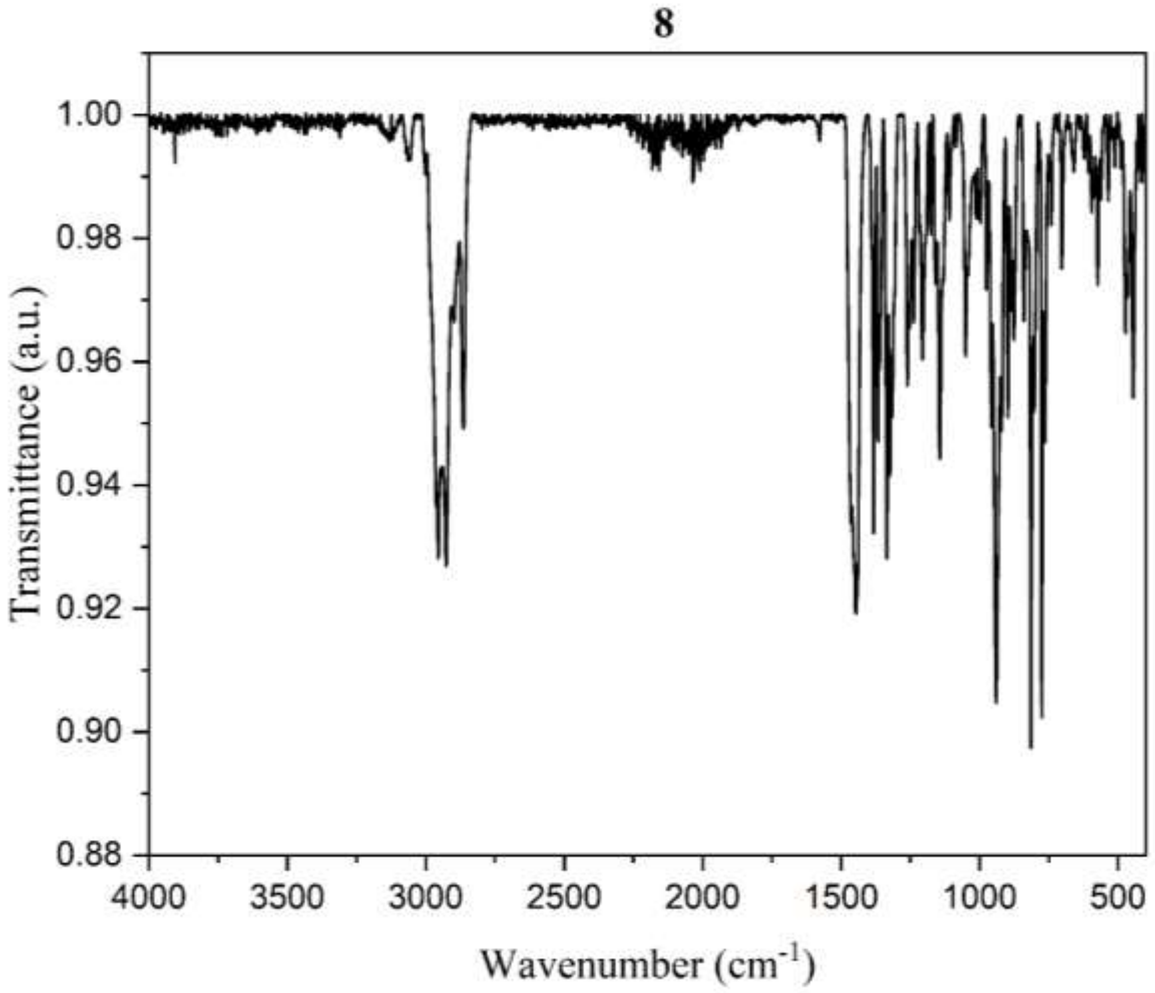

Figure S40: FTIR spectrum of **8**.

3. Crystallography

Crystallography method

The crystal data for all compounds are compiled in Table S1-4. All crystals were examined using a Bruker D8 Quest diffractometer with a Photon III detector and a microfocus source with Cu-Kα radiation (λ = 1.54178 Å). Intensities were integrated from data recorded on 1° frames by ω or φ rotation. A multi-scan absorption correction method with a beam profile was applied.[1] The structures were solved using SHELXS[2] or SHELXT;[3] the datasets were refined by full-matrix least-squares on reflections with F2≥2σ(F2) values, with anisotropic displacement parameters for all non-hydrogen atoms, and with constrained riding hydrogen geometries;[4] Uiso(H) was set at 1.2 (1.5 for methyl groups) times Ueq of the parent atom. The largest features in final difference syntheses were close to heavy atoms and were of no chemical significance. SHELX[2,3] was employed through OLEX2[4] for structure solution and refinement. The structures have been deposited with the Cambridge Crystallographic Data Centre (CCDC 2365596-2365605, 2377561, 2377566 and 2380721). This information can be obtained free of charge from www.ccdc.cam.ac.uk/data_request/cif.

Structure of **5**

The heteroleptic calcium bis(amide) complex **5** crystallizes in the monoclinic $P2_1/n$ space group with a single molecule in the asymmetric unit (Figure S5). Each three-coordinate calcium center is coordinated by a terminal NDipp ligand and two bridging N″ ligands, creating a [Ca-N-Ca-N] four-membered ring at the core of the dimer. The coordination geometry about each calcium center is distorted trigonal planar with bond angles between the terminal NDipp ligands and the bridging N″ ligands in the range of 128.9(4)-129.5(4)°, whilst the angles between the two bridging amides (N″) are 87.5(4) and 86.92(4)° [N″–Ca–N″]. The Ca–$N_{terminal}$ distances [2.286(2)-2.305(1) Å] are comparable to those of **2-Ca·(THF)$_2$** [2.301(6)-2.326(8) Å] and to the Ca–$N_{terminal}$ distances [2.266(6)-2.281(6) Å] of the closely-related dimeric bis(amide) complex [Ca(N″)(μ-N″)]$_2$ which shares the same four-membered ring core.[5,6] The Dipp substituents are on opposing sides of the [Ca-N-Ca-N] core with a torsion angle of −83.93° between the aryl functionalities of the two terminal NDipp ligands. Complex **5** is also closely related to the heteroleptic, mixed-amide dimer [{Ca{N(C_6F_5)$_2$}(μ-N″)}$_2$] reported by Harder and co-workers which also features bridging N″ ligands.[7] The authors note that π-

stacking interactions between the perfluorinated diphenylamide ligands strongly influences the terminal position of the $\{N(C_6F_5)_2\}^-$ amides. No π-stacking interactions were determined for **5** and so we attribute the bonding modes simply to steric factors.

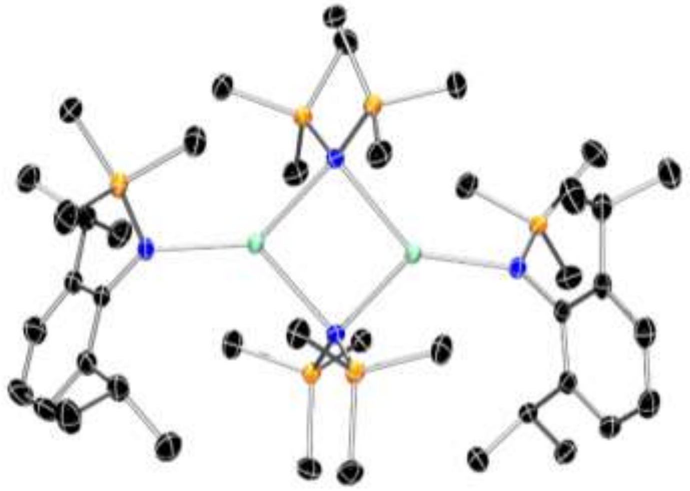

5

Figure S5: Crystal structure of mixed-amide complex **5**. Ellipsoids are set at the 50% probability level. Hydrogen atoms have been omitted for clarity. Legend: carbon (black), nitrogen (blue), silicon (orange), calcium (aquamarine).

Table S1: Crystallographic data for **1-Ca, 1-Sr** and **5**

	**1-Ca**	**1-Sr**	**2-Ca·(THF)$_2$**	**5**
Formula	$C_{24}H_{40}CaN_2Si_2$	$C_{24}H_{40}N_2Si_2Sr$	$C_{38}H_{68}CaN_2O_2Si_2$	$C_{42}H_{88}Ca_2N_4Si_6$
Formula Weight	452.84	500.38	681.20	897.86
Crystal Size, mm	0.12×0.21×0.12	0.15×0.20×0.23	0.17×0.33×0.38	0.29×0.36×0.36
Crystal System	triclinic	triclinic	orthorhombic	monoclinic
Space group	*P*-1	*P*-1	*Pccn*	$P2_1/n$
a, Å	10.0715(4)	10.2668(5)	25.0954(10)	16.3180(7)
b, Å	10.3529(5)	10.4570(5)	17.8166(7)	17.9094(7)
c, Å	13.8297(6)	13.8335(7)	18.1809(7)	18.7709(7)
α, °	81.449(2)	82.708(2)	90	90
β, °	73.155(2)	73.473(2)	90	97.144(1)
γ, °	69.368(2)	68.581(2)	90	90
V, Å^3	1289.87(10)	1325.03(11)	8129.0(6)	5443.1(4)
Z	2	2	8	4
Temperature, K	120(2)	120(2)	120(2)	120(2)
ρ_{calc}, g cm^{-3}	1.166	1.254	1.113	1.096
μ, mm^{-1}	3.065	3.769	2.131	3.303
F(*000*)	492	528	2992	1968
No. of reflections (unique)	24326 (5049)	21472 (5142)	68793 (8021)	119920 (10738)
S[a]	1.05	1.03	1.05	1.13
$R_1(wR_2)$ ($F^2 > 2\sigma(F^2)$)	0.0281 (0.0766)	0.0258 (0.0679)	0.034 (0.0862)	0.0298 (0.0783)
R_{int}	0.035	0.024	0.025	0.031
Min./max. diff map, Å^{-3}	–0.23, 0.34	–1.12, 0.87	–0.33, 0.42	–0.26, 0.37

[a]Conventional $R = \Sigma||Fo| - |Fc||/\Sigma|Fo|$; $Rw = [\Sigma w(Fo^2 - Fc^2)^2/\Sigma w(Fo^2)^2]^{1/2}$; $S = [\Sigma w(Fo^2 - Fc^2)^2$/no. data – no. params)]$^{1/2}$ for all data.

Table S2: Crystallographic data for **3-Ca**, **3-Sr** and **3-Ba**.

	**3-Ca**	**3-Sr**	**3-Ba**
Formula	$C_{83.5}H_{156}Ca_2N_6Si_6$	$C_{38}H_{69}N_3Si_3Sr$	$C_{41}H_{75}BaN_3Si_3$
Formula Weight	1492.83	739.85	789.57
Crystal Size, mm	0.33×0.40×0.42	0.09×0.014×0.014	0.15×0.19×0.26
Crystal System	triclinic	monoclinic	monoclinic
Space group	*P*-1	*C*2/*c*	$P2_1/c$
a, Å	12.3188(3)	36.8477(13)	9.9833(4)
b, Å	18.6035(5)	12.8589(5)	19.6974(7)
c, Å	21.8529(6)	22.8669(8)	22.7415(8)
α, °	98.403(1)	90	90
β, °	104.813(1)	126.000(2)	100.885(2)
γ, °	92.991(1)	90	90
V, $Å^3$	4768.6(2)	8765.6(6)	4391.5(3)
Z	4	8	4
Temperature, K	120(2)	120(2)	120(2)
ρ_{calc}, g cm^{-3}	1.015	1.121	1.194
μ, mm^{-1}	2.058	2.682	8.005
F(*000*)	1604	3184	1664
No. of reflections (unique)	107939 (20384)	105672 (8675)	113437 (9578)
S[a]	1.02	1.04	1.06
$R_1(wR_2)$ ($F^2 > 2\sigma(F^2)$)	0.0365 (0.1028)	0.0335 (0.0848)	0.0449 (0.1197)
R_{int}	0.031	0.066	0.066
Min./max. diff map, $Å^{-3}$	–0.49, 0.56	–0.75, 0.54	–0.97, 3.76

[a]Conventional $R = \Sigma||Fo| - |Fc||/\Sigma|Fo|$; $Rw = [\Sigma w(Fo^2 - Fc^2)^2/\Sigma w(Fo^2)^2]^{1/2}$; $S = [\Sigma w(Fo^2 - Fc^2)^2/\text{no. data} - \text{no. params})]^{1/2}$ for all data.

Table S3: Crystallographic data for **4-Ca**, **4-Sr** and **4-Ba**.

	**4-Ca**	**4-Sr**	**4-Ba**
Formula	$C_{41}H_{75}CaN_3Si_3$	$C_{41}H_{75}N_3Si_3Sr$	$C_{41}H_{75}BaN_3Si_3$
Formula Weight	734.39	781.93	831.65
Crystal Size, mm	0.14×0.24×0.35	0.15×0.16×0.96	0.22×0.24×0.33
Crystal System	monoclinic	monoclinic	monoclinic
Space group	$P2_1/n$	$P2_1/n$	$C2/c$
a, Å	12.3848(12)	12.4917(4)	20.3511(8)
b, Å	22.698(2)	22.9592(8)	14.1704(6)
c, Å	16.6139(2)	16.5843(6)	33.0380(13)
α, °	90	90	90
β, °	104.684(5)	105.299(1)	98.404(1)
γ, °	90	90	90
V, Å^3	4517.8(7)	4587.8(3)	9425.3(7)
Z	4	4	4
Temperature, K	120(2)	120(2)	120(2)
ρ_{calc}, g cm^{-3}	1.080	1.132	1.172
μ, mm^{-1}	2.166	2.586	7.483
F(*000*)	1616	1688	3520
No. of reflections (unique)	65728 (7607)	83878 (9022)	92035 (9322)
S[a]	1.05	1.02	1.14
$R_1(wR_2)$ ($F^2 > 2\sigma(F^2)$)	0.1046 (0.2980)	0.232 (0.0615)	0.0323 (0.0795)
R_{int}	0.146	0.040	0.045
Min./max. diff map, Å^{-3}	–0.44, 0.56	–0.32, 0.31	–0.65, 1.20

[a]Conventional $R = \Sigma||Fo| - |Fc||/\Sigma|Fo|$; $Rw = [\Sigma w(Fo^2 - Fc^2)^2/\Sigma w(Fo^2)^2]^{1/2}$; $S = [\Sigma w(Fo^2 - Fc^2)^2/\text{no. data} - \text{no. params})]^{1/2}$ for all data.

Table S4: Crystallographic data for **6**, **7** and **8**.

	**6**	**7**	**8**
Formula	$C_{32}H_{52}N_2Si$	$C_{47}H_{78}CaN_3Si_2$	$C_{32}H_{67}CaN_3Si_4$
Formula Weight	492.84	867.55	646.32
Crystal Size, mm	0.01×0.02×0.03	0.04×0.09×0.86	0.12×0.13×0.41
Crystal System	orthorhombic	monoclinic	monoclinic
Space group	$P2_12_12_1$	$C2/c$	$P2_1/c$
a, Å	10.4804(4)	40.1466(13)	9.9703(5)
b, Å	16.2690(6)	15.7878(5)	20.9256(11)
c, Å	17.1980(8)	18.6395(6)	19.8352(10)
α, °	90	90	90
β, °	90	112.165(2)	103.419(3)
γ, °	90	90	90
V, Å^3	2932.4(2)	10941.2(6)	4025.3(4)
Z	4	8	4
Temperature, K	120(2)	120(2)	120(2)
ρ_{calc}, g cm^{-3}	1.116	1.053	1.066
μ, mm^{-1}	0.849	1.651	2.645
F(*000*)	1088	3832	1424
No. of reflections (unique)	75351 (5757)	85629 (10807)	39446 (7966)
S[a]	1.03	1.03	1.02
$R_1(wR_2)$ ($F^2 > 2\sigma(F^2)$)	0.0320 (0.0784)	0.0438 (0.1217)	0.0333 (0.0840)
R_{int}	0.100	0.061	0.062
Min./max. diff map, Å^{-3}	–0.22, 0.18	–0.44, 0.56	–0.22, 0.34

[a]Conventional $R = \Sigma||Fo| - |Fc||/\Sigma|Fo|$; $Rw = [\Sigma w(Fo^2 - Fc^2)^2/\Sigma w(Fo^2)^2]^{1/2}$; $S = [\Sigma w(Fo^2 - Fc^2)^2/\text{no. data} - \text{no. params})]^{1/2}$ for all data.

4. Computational details

Table S5: LUMO of [AE(NMes)(N″)(CAAC)] (**3-AE**), SOMO and spin density plot of putative reduced species [AE(NMes)(N″)(CAAC)]$^{-}$. Surfaces displayed at isovalue of 0.04 for molecular orbitals and 0.004 for spin density plots. Hydrogen atoms have been omitted for clarity.

**Compound**	**LUMO AE(II)** [AE(L)$_2$(CAAC)]	**α-SOMO AE(I)** [AE(L)$_2$CAAC)]$^{-}$	**β-SOMO AE(I)** [AE(L)$_2$CAAC)]$^{-}$	**Spin density** [AE(L)$_2$CAAC)]$^{-}$
**3-Ca**	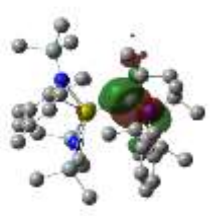	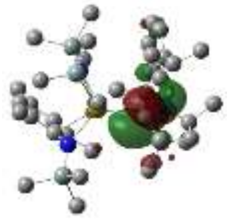	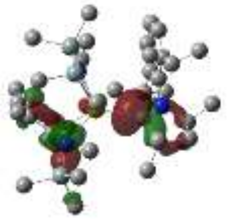	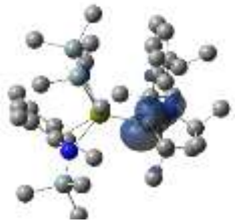
**3-Sr**	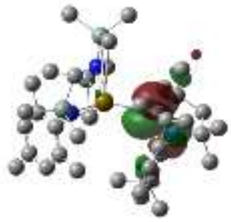	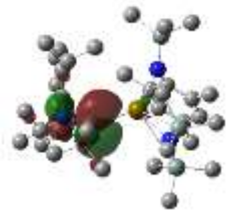	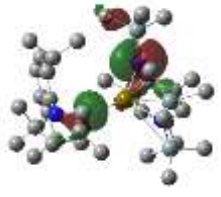	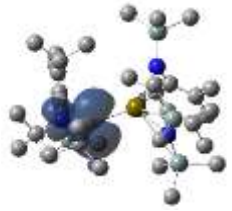
**3-Ba**	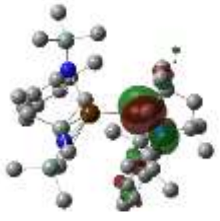	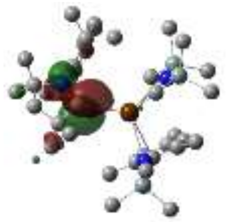	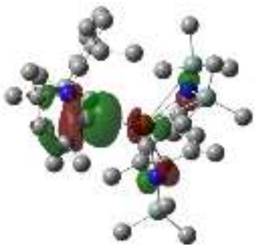	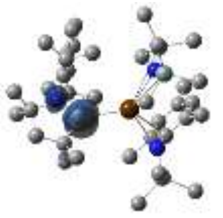

Table S6: LUMO of [AE(NDipp)(N″)(CAAC)] (**4-AE**), SOMO and spin density plot of putative reduced species [AE(NDipp)(N″)(CAAC)]$^{–}$. Surfaces displayed at isovalue of 0.04 for molecular orbitals and 0.004 for spin density plots. Hydrogen atoms have been omitted for clarity.

**Compound**	**LUMO AE(II)** **[AE(L)$_2$(CAAC)]**	**α-SOMO AE(I)** **[AE(L)$_2$CAAC)]$^{–}$**	**β-SOMO AE(I)** **[AE(L)$_2$CAAC)]$^{–}$**	**Spin density** **[AE(L)$_2$CAAC)]$^{–}$**
**4-Ca**	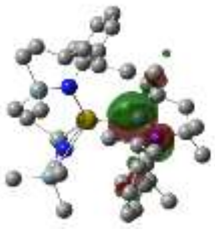	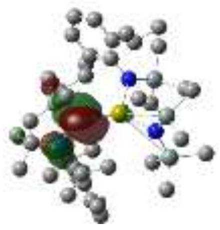	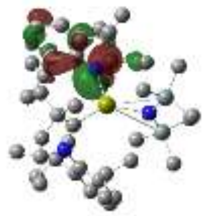	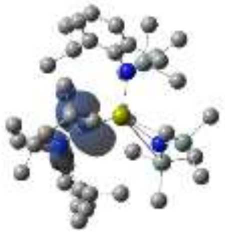
**4-Sr**	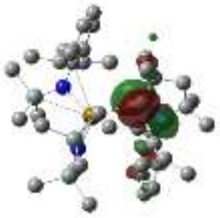	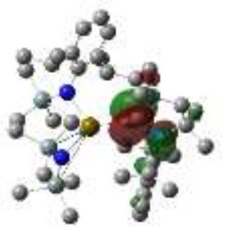	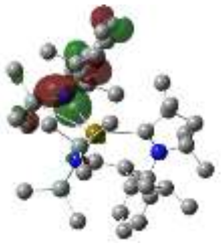	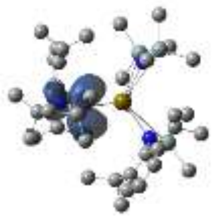
**4-Ba**	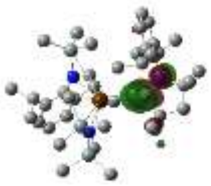	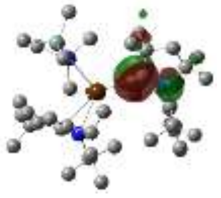	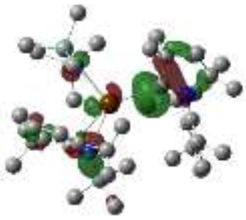	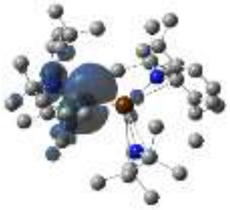

Table S7: LUMO of [AE(NMes)$_2$(CAAC)] (**7**) and [AE (N″)$_2$(CAAC)] (**8**), SOMO and spin density plot of putative reduced species [AE(L)$_2$(CAAC)]$^{-}$. Surfaces displayed at isovalue of 0.04 for molecular orbitals and 0.004 for spin density plots. Hydrogen atoms have been omitted for clarity.

Compound	LUMO AE(II) [AE(L)$_2$(CAAC)]	α-SOMO AE(I) [AE(L)$_2$CAAC)]$^{-}$	β-SOMO AE(I) [AE(L)$_2$CAAC)]$^{-}$	Spin density [AE(L)$_2$CAAC)]$^{-}$
**7**	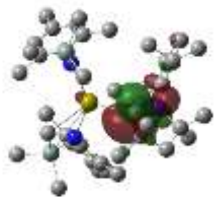	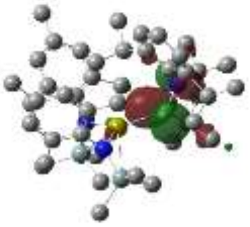	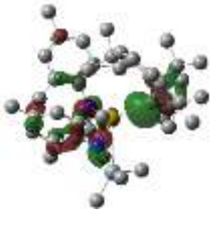	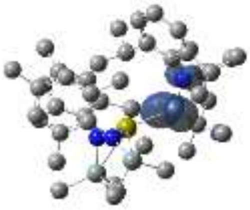
**8**	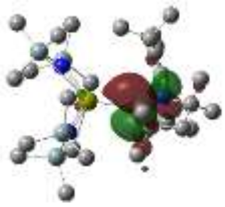	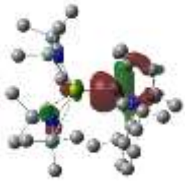	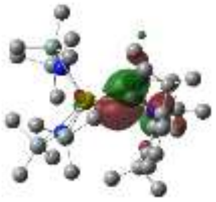	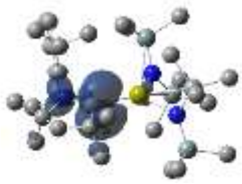

Table S8: Bond lengths (Å) of AE–N and AE–C$_{carbene}$ in divalent AE(II) **3-AE**, **4-AE**, **7**, and **8**, and respective putative reduced AE(I) species [AE(L)$_2$(CAAC)]$^{-}$. N(1) is nitrogen donor of N″ ligand, N(2) is nitrogen donor of NAr ligand (Ar = Mes, Dipp), with the exception of **7** and **8** where the two donors are the same.

**Compound**	**AE–N(1)**			**AE–N(2)**			**AE–C**		
	**AE(II)**	**AE(I)**	**Δ**	**AE(II)**	**AE(I)**	**Δ**	**AE(II)**	**AE(I)**	**Δ**
**3-Ca**	2.257	2.306	0.049	2.242	2.303	0.061	2.581	2.421	-0.160
**3-Sr**	2.399	2.455	0.056	2.432	2.513	0.081	2.768	2.577	-0.191
**3-Ba**	2.550	2.631	0.081	2.532	2.609	0.077	2.966	2.732	-0.234
**4-Ca**	2.245	2.312	0.067	2.271	2.323	0.051	2.600	2.422	-0.178
**4-Sr**	2.397	2.464	0.066	2.415	2.471	0.055	2.737	2.567	-0.170
**4-Ba**	2.538	2.617	0.079	2.539	2.634	0.095	2.985	2.737	-0.248
**7**	2.271	2.325	0.054	2.233	2.300	0.067	2.573	2.410	-0.163
**8**	2.255	2.315	0.059	2.249	2.320	0.071	2.635	2.419	-0.216

Table S9: NPA charges on the metal in divalent AE(II) **3-AE**, **4-AE**, **7**, and **8**, and respective putative reduced AE(I) species $[AE(L)_2(CAAC)]^-$.

Compound	AE(II)	AE(I)	Δ
**3-Ca**	1.789	1.786	-0.003
**3-Sr**	1.805	1.790	-0.015
**3-Ba**	1.819	1.804	-0.015
**4-Ca**	1.786	1.785	-0.001
**4-Sr**	1.801	1.803	0.002
**4-Ba**	1.815	1.793	-0.022
**7**	1.790	1.774	-0.016
**8**	1.774	1.763	-0.011

Table S10: NPA charges on C(1) in divalent AE(II) **3-AE**, **4-AE**, **7**, and **8**, and respective putative reduced AE(I) species $[AE(L)_2(CAAC)]^-$.

Compound	AE(II)	AE(I)	Δ
**3-Ca**	0.032	-0.455	-0.487
**3-Sr**	0.032	-0.472	-0.504
**3-Ba**	0.046	-0.466	-0.512
**4-Ca**	0.018	-0.477	-0.495
**4-Sr**	0.022	-0.464	-0.486
**4-Ba**	0.053	-0.395	-0.448
**7**	0.022	-0.484	-0.506
**8**	0.046	-0.460	-0.506

Table S11: NPA charges on N(3) in divalent AE(II) **3-AE**, **4-AE**, **7**, and **8**, and respective putative reduced AE(I) species $[AE(L)_2(CAAC)]^-$.

**Compound**	**AE(II)**	**AE(I)**	**Δ**
**3-Ca**	-0.478	-0.605	-0.127
**3-Sr**	-0.478	-0.617	-0.139
**3-Ba**	-0.484	-0.613	-0.129
**4-Ca**	-0.470	-0.616	-0.146
**4-Sr**	-0.475	-0.621	-0.146
**4-Ba**	-0.481	-0.620	-0.139
**7**	-0.472	-0.621	-0.149
**8**	-0.490	-0.626	-0.136

Coordinates

Divalent

3-Ca

Lowest frequency: 10.5421 cm^{-1}

Second frequency: 14.5204 cm^{-1}

Ca	0.72056	0.21420	-0.38739
Si	0.73314	2.64728	1.64879
N	0.98902	2.40969	-0.02071
C	-1.48655	-0.82315	-1.23185
Si	1.50618	3.48090	-1.24392
N	2.43114	-1.19505	-0.81281
C	-1.50207	-1.46505	-2.61482
Si	3.37788	-1.86597	-2.07383
N	-2.68676	-0.96342	-0.70312
C	-2.99330	-1.79403	-2.88569
H	-3.43381	-1.01502	-3.51701
H	-3.11598	-2.74873	-3.40265
C	-3.68961	-1.80064	-1.51527
C	-0.58423	-2.70110	-2.62981
H	0.41387	-2.43802	-2.26658
H	-0.49658	-3.08493	-3.65224
H	-0.97088	-3.50275	-1.99387
C	-0.97540	-0.42568	-3.62031
H	-1.55616	0.50178	-3.57411
H	-1.04429	-0.82608	-4.63835
H	0.07353	-0.18176	-3.43061
C	-3.78512	-3.21638	-0.93763
H	-2.80166	-3.68504	-0.85475
H	-4.39930	-3.82781	-1.60725
H	-4.25993	-3.21503	0.04432
C	-5.07208	-1.15809	-1.52781
H	-5.05641	-0.18270	-2.01586
H	-5.46200	-1.03691	-0.51248
H	-5.76075	-1.80350	-2.08291
C	-3.08123	-0.23726	0.48886
C	-3.03853	-0.83799	1.76189
C	-3.41096	-0.06060	2.86802
H	-3.37479	-0.50389	3.85704
C	-3.79460	1.26788	2.72371
H	-4.06329	1.85544	3.59501
C	-3.81409	1.84792	1.45801
H	-4.09485	2.88962	1.34964
C	-3.46239	1.11490	0.31913
C	-2.57182	-2.26167	2.00912
H	-2.28002	-2.69861	1.05015
C	-1.33527	-2.29829	2.92108
H	-0.49420	-1.75217	2.49196
H	-1.01836	-3.33455	3.07956
H	-1.55169	-1.85914	3.90100
C	-3.69700	-3.11530	2.62276
H	-3.91582	-2.78494	3.64457
H	-3.39557	-4.16759	2.66946
H	-4.62813	-3.04462	2.05129
C	-3.47633	1.81522	-1.03163
H	-3.28165	1.06831	-1.80808
C	-4.84176	2.46095	-1.32638
H	-4.85407	2.86393	-2.34524
H	-5.03574	3.29377	-0.64183
H	-5.66564	1.74773	-1.22399
C	-2.36325	2.87149	-1.11967
H	-2.30685	3.27923	-2.13551
H	-1.37995	2.47403	-0.84777
H	-2.56706	3.70402	-0.43716
C	-0.65158	3.86099	2.07893
H	-0.46880	4.84274	1.62676
H	-0.73295	3.99384	3.16579
H	-1.61213	3.48819	1.70853
C	0.18036	0.96798	2.36535
H	-0.74652	0.60213	1.89691
H	-0.06475	1.09745	3.42635
H	0.96094	0.19215	2.33639
C	2.26874	3.18293	2.60788
H	3.07822	2.46066	2.46036
H	2.06593	3.26031	3.68393
H	2.62028	4.16175	2.25779
C	0.64081	5.16388	-1.18824
H	-0.44923	5.04895	-1.17729
H	0.91417	5.78221	-2.05324
H	0.92965	5.71072	-0.28129
C	3.36585	3.82716	-1.22740
H	3.68643	4.17617	-0.23808
H	3.63360	4.59540	-1.96471
H	3.93535	2.91932	-1.46013
C	1.12188	2.68543	-2.92246
H	1.62627	1.71327	-3.01846
H	1.47788	3.31227	-3.74970
H	0.04326	2.53416	-3.05296
C	2.84559	-1.09012	-3.71011
H	1.83048	-1.38487	-3.99767
H	3.52283	-1.41428	-4.51069
H	2.89098	0.00443	-3.65911
C	5.24501	-1.59285	-1.90350
H	5.52141	-0.55086	-2.10485
H	5.78709	-2.22796	-2.61661
H	5.59102	-1.84953	-0.89437

C	3.19958	-3.74728	-2.26889
H	3.54206	-4.26364	-1.36264
H	3.81470	-4.10266	-3.10655
H	2.16258	-4.04817	-2.46152
C	2.67215	-1.38055	0.56978
C	3.50550	-0.47642	1.29936
C	3.63654	-0.61233	2.68404
H	4.26961	0.09329	3.21766
C	2.97300	-1.61456	3.40388
C	2.15126	-2.49027	2.69018
H	1.62097	-3.27391	3.22646
C	1.98078	-2.38742	1.30336
C	4.19010	0.65740	0.59013
H	4.76544	0.30898	-0.26831
H	4.86221	1.19138	1.26891
H	3.45676	1.37951	0.21041
C	3.13107	-1.72811	4.89793
H	4.17634	-1.90820	5.17861
H	2.53220	-2.55243	5.29809
H	2.81471	-0.80782	5.40490
C	1.04471	-3.32378	0.58939
H	0.18138	-2.78969	0.16665
H	0.66131	-4.08815	1.27230
H	1.53632	-3.81575	-0.25250

3-Sr

Lowest frequency: 15.8655 cm^{-1}
Second frequency: 23.7314 cm^{-1}

Sr	8.17248040	9.09479433	7.55755421
Si	8.50088230	10.52726624	10.64084503
Si	6.35506961	11.75215710	8.68907027
Si	7.77411682	5.21280373	8.14339825
N	7.34630602	6.81359609	7.72633688
N	7.62749297	10.75125750	9.20494665
C	6.08900802	7.23143497	7.25533669
C	5.11899555	7.78004086	8.15097977
N	10.74059288	9.95927627	4.97597831
C	5.78149989	7.22855032	5.86206581
C	10.38555977	8.73047971	4.30090269
C	3.93212067	8.32160451	7.64774882
H	3.20685758	8.73142434	8.34714741
C	10.20928972	10.30928146	6.13062924
C	6.03639203	11.34851144	6.85096722
H	6.93651486	11.49985068	6.23443090
H	5.26820541	12.02061698	6.44787058
H	5.64853580	10.32891383	6.70442719
C	4.58904490	7.80006408	5.40209520
H	4.38329515	7.79606540	4.33325308
C	3.65172747	8.36204931	6.27554919
C	6.73626202	6.57321187	4.90647114
H	7.76918516	6.90008757	5.07013649
H	6.46434725	6.77609113	3.86700911
H	6.74776390	5.48662751	5.04895202
C	9.40015071	8.75966362	3.29196889
C	5.39489061	7.78267664	9.62876185
H	5.80011116	6.82158164	9.95427714
H	4.48445262	7.99946960	10.19645320
H	6.13649448	8.54879707	9.89185772
C	11.00919918	7.52774883	4.69812494
C	8.55307992	9.99213982	3.01571370
H	8.98404573	10.83106028	3.57072230
C	10.68724277	6.35922947	3.99564690
H	11.14391770	5.42090677	4.29025532
C	9.12693528	7.56813431	2.60905151
H	8.37206203	7.56535873	1.83056756
C	11.21424441	6.72383768	7.06735134
H	10.19154233	7.08665340	7.20971981
H	11.76809947	6.85882016	8.00252322
H	11.13162470	5.65034336	6.87335988
C	11.92418104	7.44589199	5.90924921
H	12.13028256	8.46474870	6.24478817
C	4.72635890	11.48298697	9.60503423
H	4.41492246	10.43693087	9.52920667
H	3.92481597	12.11223920	9.19659861
H	4.84164332	11.72497406	10.66891508
C	7.12576965	9.78125623	3.55184568
H	6.54131725	10.70337188	3.46771680
H	7.12115872	9.47676574	4.60149143
H	6.60959762	8.99516263	2.99171502
C	9.77495040	6.38153355	2.94338425
H	9.54001496	5.46632446	2.41079372
C	10.88639439	11.59666969	6.58118334
C	6.72107172	13.60756584	8.79659337
H	6.91555373	13.88954608	9.83947965
H	5.86781405	14.20022562	8.44106881
H	7.60152365	13.88683061	8.20718966
C	2.39497654	9.01479405	5.76104095
H	1.53605688	8.79241943	6.40414635
H	2.15476531	8.67437152	4.74831483
H	2.49837537	10.10792031	5.72560412
C	8.50770911	10.36574462	1.52650539
H	8.00224815	9.58960543	0.94130549
H	9.50968151	10.50069921	1.10923296
H	7.94744714	11.29713944	1.38875259
C	9.83654298	12.62194273	7.02702904
H	9.15466211	12.86983925	6.20536865
H	10.32722412	13.54579614	7.35478386

H	9.24194312	12.22623758	7.85812133
C	11.84756300	10.86658321	4.41417889
C	8.57488974	4.21087365	6.73353257
H	7.80334678	3.72564821	6.12188866
H	9.23099794	3.42300986	7.12787832
H	9.16206905	4.85688260	6.07186146
C	11.70460529	12.07937629	5.35168017
H	11.15623319	12.87832254	4.83996983
H	12.68110485	12.47943498	5.63977617
C	9.01109341	5.24825137	9.57193477
H	9.89620454	5.85242113	9.34239403
H	9.35076154	4.23443068	9.82031787
H	8.53673988	5.67650795	10.46359277
C	6.27241032	4.20015467	8.69068611
H	5.87979573	4.54725389	9.65378135
H	6.52493588	3.13641936	8.78952742
H	5.46650417	4.29203220	7.95114021
C	11.59747907	11.21364697	2.94892934
H	11.55538027	10.31239625	2.32977593
H	12.42290673	11.83540907	2.58677840
H	10.67168125	11.77707389	2.81976873
C	13.26555207	6.76312956	5.60398813
H	13.11971490	5.71441789	5.32301967
H	13.90596772	6.77952469	6.49284233
H	13.79918346	7.25846868	4.78743035
C	13.20322028	10.16472881	4.53469649
H	13.46511407	9.95037313	5.57273582
H	13.97937090	10.81429113	4.11733558
H	13.20509114	9.22878889	3.96896466
C	11.79148947	11.24343626	7.78147281
H	11.19475724	10.86222798	8.61379057
H	12.31009910	12.14579327	8.12363549
H	12.54400609	10.49111814	7.52326760
C	7.44684287	10.17380002	12.16943432
H	6.78483324	11.02371843	12.37850013
H	8.06735341	10.00032714	13.05832218
H	6.81503550	9.29149374	12.01374916
C	9.63052279	11.97992547	11.08729092
H	10.29209706	12.24673251	10.25503963
H	10.25161723	11.75762895	11.96484423
H	9.02141020	12.86335535	11.31800024
C	9.60559685	8.98838620	10.38952193
H	9.00134649	8.08884732	10.19997725
H	10.18481400	8.78713078	11.29936426
H	10.34215882	9.10086224	9.57767751

3-Ba

Lowest frequency: 12.4106 cm^{-1}

Second frequency: 18.4367 cm^{-1}

Ba	-0.52410	0.07382	-0.19072
N	3.12767	-0.92191	-0.59127
C	2.01168	-0.99981	-1.29189
Si	-0.93515	3.02474	1.69696
N	-0.98321	2.55103	0.06645
C	2.31123	-1.79208	-2.55483
Si	-1.45243	3.34288	-1.36235
N	-2.58809	-1.25931	-0.87140
C	3.85971	-1.93900	-2.58974
H	4.16936	-2.93635	-2.91559
H	4.28031	-1.21395	-3.29583
Si	-3.43094	-1.54056	-2.32132
C	4.36508	-1.62848	-1.16772
C	1.78651	-1.02353	-3.77703
H	2.04923	-1.55743	-4.69767
H	0.69793	-0.92664	-3.73985
H	2.22111	-0.01870	-3.82572
C	1.58716	-3.14959	-2.44611
H	0.50716	-3.00193	-2.35216
H	1.77536	-3.74211	-3.34836
H	1.92848	-3.72479	-1.57899
C	5.58619	-0.71349	-1.15088
H	5.44257	0.15876	-1.79015
H	5.81294	-0.37500	-0.13503
H	6.45313	-1.26728	-1.52595
C	4.67336	-2.89271	-0.36169
H	4.91828	-2.64542	0.67400
H	3.83848	-3.59715	-0.37033
H	5.54199	-3.39228	-0.80304
C	3.17964	-0.17597	0.65133
C	2.86370	-0.81105	1.87266
C	2.90974	-0.04201	3.04447
H	2.66233	-0.50833	3.99189
C	3.24147	1.30926	3.00942
H	3.26202	1.88829	3.92619
C	3.50628	1.92511	1.79143
H	3.72154	2.98670	1.76327
C	3.46887	1.20680	0.59140
C	2.41898	-2.26212	1.97345
H	2.49331	-2.71152	0.98003
C	3.30642	-3.06569	2.93985
H	3.16557	-2.72808	3.97254
H	3.04316	-4.12819	2.89939
H	4.36913	-2.96400	2.70045
C	0.94849	-2.36843	2.41125
H	0.66363	-3.41621	2.54841
H	0.76350	-1.83993	3.35178
H	0.25990	-1.96493	1.66506
C	3.65740	1.95833	-0.71680

H	3.64330	1.22947	-1.53283
C	2.49165	2.93406	-0.95369
H	1.51144	2.46368	-0.83441
H	2.52164	3.76209	-0.23748
H	2.55358	3.35807	-1.96208
C	4.99432	2.71728	-0.76291
H	5.12879	3.18264	-1.74572
H	5.01291	3.51773	-0.01519
H	5.84727	2.06034	-0.56992
C	-2.53956	3.79340	2.33687
H	-3.38677	3.12354	2.15153
H	-2.48965	3.99981	3.41408
H	-2.74284	4.74030	1.82052
C	-0.60961	1.47924	2.77333
H	-1.40809	0.72557	2.69877
H	0.36569	1.01156	2.56945
H	-0.57698	1.78887	3.82621
C	0.46071	4.23542	2.10937
H	1.43146	3.78172	1.88112
H	0.36425	5.14941	1.51040
H	0.45235	4.51794	3.17045
C	-3.30006	3.72885	-1.46443
H	-3.89244	2.81101	-1.37286
H	-3.59847	4.39905	-0.64880
H	-3.56102	4.21158	-2.41542
C	-1.07562	2.16849	-2.81809
H	-0.01248	1.88847	-2.86619
H	-1.69026	1.25752	-2.76849
H	-1.32330	2.65252	-3.77110
C	-0.53571	4.96582	-1.69208
H	-0.77705	5.70005	-0.91244
H	0.54967	4.81668	-1.68410
H	-0.81861	5.39885	-2.66061
C	-4.61413	-0.14893	-2.82980
H	-5.49974	-0.12408	-2.18257
H	-4.12150	0.82860	-2.75876
H	-4.95913	-0.28304	-3.86400
C	-4.45009	-3.13505	-2.28458
H	-3.80697	-4.02281	-2.32375
H	-5.03660	-3.18577	-1.35832
H	-5.14679	-3.18352	-3.13133
C	-2.16005	-1.67071	-3.71870
H	-1.56350	-0.75222	-3.79488
H	-1.47956	-2.51224	-3.53991
H	-2.65002	-1.82550	-4.68832
C	-2.86036	-1.61731	0.44912
C	-2.31855	-2.81302	1.00988
C	-2.46714	-3.08261	2.37597
H	-2.04952	-4.00478	2.77624
C	-3.13003	-2.20551	3.23980
C	-3.66729	-1.03333	2.69023
H	-4.19481	-0.33582	3.33820
C	-3.55132	-0.72623	1.33116
C	-1.58211	-3.77632	0.12114
H	-2.22815	-4.15095	-0.67957
H	-0.73354	-3.29528	-0.38291
H	-1.20445	-4.63023	0.69331
C	-3.25561	-2.49952	4.71272
H	-4.30162	-2.46794	5.04142
H	-2.86105	-3.49219	4.95311
H	-2.70569	-1.76723	5.31857
C	-4.14355	0.54185	0.78226
H	-4.80029	0.33193	-0.06692
H	-4.71923	1.06702	1.55064
H	-3.37503	1.23391	0.41150

4-Ca

Lowest frequency: 20.1982 cm^{-1}

Second frequency: 32.8154 cm^{-1}

Ca	2.42793373	17.23441552	9.71620103
Si	4.30581710	16.02319822	7.18104634
Si	5.79834900	17.26150177	9.53863592
Si	1.24135862	19.94309235	7.81028440
N	1.12989666	14.56149736	11.58314429
N	4.38386604	16.70407789	8.75027459
N	1.32399615	19.18773093	9.36238201
C	1.51689342	15.81847082	11.69717151
C	1.26651086	16.25148079	13.13795939
C	0.92827862	14.94094269	13.89275792
H	1.82469057	14.56259244	14.39612566
H	0.15856700	15.09497677	14.65282971
C	0.47870159	13.93183586	12.82664500
C	0.97855783	12.51639975	13.09423189
H	0.80817234	11.86416858	12.23230253
H	0.42593840	12.10436515	13.94506350
H	2.03922144	12.50263289	13.34620329
C	-1.04042854	13.88921175	12.63151978
H	-1.46098495	14.87898429	12.44652666
H	-1.50506958	13.48525525	13.53682939
H	-1.30156740	13.23383128	11.79655963
C	2.52232646	16.93223271	13.69916250
H	2.69803291	17.88156998	13.18489397
H	3.41056894	16.30219973	13.58781334
H	2.37956854	17.15064697	14.76376420
C	0.10652032	17.26310777	13.16933653
H	-0.02456848	17.63342786	14.19165840
H	-0.83721873	16.81890369	12.84418463
H	0.31633921	18.11916707	12.52662986

C	1.26061698	13.81058606	10.35208031
C	0.26876592	13.91909132	9.35400381
C	0.41844490	13.14647126	8.19318388
H	-0.32912278	13.22081924	7.41102397
C	1.51382741	12.30689420	8.02036094
H	1.61194708	11.72030032	7.11330610
C	2.50277408	12.24827734	8.99903663
H	3.37555697	11.62428322	8.84290776
C	2.40735923	13.00297051	10.17409133
C	3.56758238	12.98055811	11.15689980
H	3.27790438	13.56330709	12.03733951
C	3.92937161	11.55477896	11.60598237
H	4.70110625	11.59179782	12.38300243
H	4.33677789	10.97680104	10.76913802
H	3.06697807	11.00940281	11.99938242
C	4.80212705	13.66383876	10.54369571
H	5.58148264	13.78497738	11.30470678
H	4.56153412	14.64433350	10.12066385
H	5.21698231	13.05506023	9.73328467
C	-0.91663652	14.86792110	9.44397998
H	-0.91484346	15.33058691	10.43400589
C	-2.25668762	14.13575146	9.25911545
H	-3.08630757	14.82046115	9.46521745
H	-2.34719461	13.27260547	9.92526097
H	-2.37011541	13.77613424	8.23039255
C	-0.79767458	15.99900900	8.40771101
H	-1.72501001	16.57645907	8.36713306
H	-0.59486668	15.60550169	7.40714812
H	-0.00638634	16.71410215	8.65676844
C	2.47196681	15.93035296	6.70536479
H	1.92121879	15.27925306	7.39874521
H	2.35146427	15.49417089	5.70584035
H	2.00028773	16.92080703	6.68007519
C	4.96301269	14.25501065	7.03925769
H	4.34412661	13.56669610	7.62263364
H	5.99409235	14.18577591	7.40495595
H	4.94910016	13.92549457	5.99184912
C	5.23185434	17.01106573	5.85955380
H	4.89850499	18.05307156	5.81925674
H	5.09179833	16.56540355	4.86592753
H	6.30836247	17.01183325	6.07583405
C	7.32181257	16.17736809	9.25237257
H	7.13505856	15.14408013	9.56715392
H	8.18618403	16.55650438	9.81281065
H	7.59272481	16.16088617	8.18912366
C	5.46835211	17.26922908	11.40765626
H	4.57289607	17.84564233	11.67337965
H	6.30299502	17.72545951	11.95434827
H	5.33419144	16.24620431	11.77769784
C	6.25865680	19.02362015	9.03614821
H	6.49408061	19.06083571	7.96553034
H	7.12677652	19.39903892	9.59342016
H	5.41622508	19.70350110	9.20760110
C	0.02754406	19.11303148	6.60976680
H	0.20162727	18.03526533	6.51310924
H	0.13863767	19.55784205	5.61156007
H	-1.01146719	19.25892755	6.92787419
C	2.94933087	19.82639876	7.01631992
H	3.61815152	20.58537875	7.43905015
H	2.91058450	19.96743127	5.92871785
H	3.41211767	18.85448076	7.22062494
C	0.71897147	21.76474396	7.92562604
H	-0.11212119	21.97935804	7.24271925
H	1.54611882	22.43479915	7.66201380
H	0.39626496	22.01462866	8.94352546
C	0.83905364	19.76918068	10.55135777
C	1.75692443	20.31280050	11.50915738
C	1.27969438	20.83917210	12.71502056
H	1.98460377	21.24480826	13.43367189
C	-0.08468994	20.88375862	13.00143034
H	-0.43660455	21.30983102	13.93567541
C	-0.98707360	20.38443654	12.06536849
H	-2.05276584	20.42528823	12.27636638
C	-0.55351399	19.81638493	10.86204620
C	-1.58148725	19.25400710	9.89847957
H	-1.01605716	18.77683914	9.09320108
C	-2.45230029	20.36279182	9.28549986
H	-3.03910894	20.87096401	10.06051238
H	-3.15213895	19.94587915	8.55027432
H	-1.83545699	21.11434496	8.78599390
C	-2.46491210	18.18228214	10.55648428
H	-1.85775207	17.34867859	10.92277384
H	-3.19504226	17.78810680	9.83866006
H	-3.02332173	18.58971275	11.40730938
C	3.23315970	20.43273905	11.16427494
H	3.50289237	19.58134937	10.52160589
C	3.47109820	21.69647433	10.31761309
H	3.20516085	22.58761109	10.89825055
H	2.85671295	21.68254194	9.41732172
H	4.52386389	21.77483707	10.02133839
C	4.17482465	20.44772022	12.37381395
H	5.21335520	20.37132687	12.03498927
H	3.98115643	19.62103412	13.06466051
H	4.08057673	21.38385737	12.93643219

4-Sr

Lowest frequency: 21.4865 cm^{-1}

Second frequency: 27.5963 cm^{-1}

Sr	2.33130993	5.55569493	9.58638856
H	1.97128938	5.84573931	6.61414347
H	4.49566199	4.96538342	11.53988696
Si	4.31168145	6.75015170	7.03418399
Si	5.81847351	5.55165519	9.45448579
Si	1.13145085	2.71437842	7.65777165
N	0.97288718	8.30060786	11.52299145
N	4.44844044	6.09156903	8.59788848
N	1.19508915	3.45911400	9.20193732
C	1.36616524	7.04924549	11.66707043
C	1.08642541	6.63242696	13.10310519
C	0.71596984	7.95000402	13.83737889
H	-0.08567926	7.80049229	14.56629113
H	1.59165466	8.33123802	14.37589707
C	0.30850623	8.95535998	12.74534808
C	-1.20489313	9.02447911	12.52144789
H	-1.43848107	9.66625686	11.66781911
H	-1.67843811	9.45662345	13.40759727
H	-1.64115240	8.03837831	12.35115474
C	0.83753647	10.36226708	13.00109198
H	1.89808799	10.35363260	13.25524920
H	0.29256616	10.79702492	13.84556934
H	0.68503374	11.00857829	12.13127498
C	-0.06758142	5.61173962	13.09850156
H	-0.24075473	5.25029025	14.11765698
H	0.17361817	4.74875531	12.47434519
H	-0.99953985	6.04691456	12.72767763
C	2.33415293	5.96446197	13.69917335
H	3.21803664	6.60462642	13.60860106
H	2.53569446	5.01620797	13.19101137
H	2.16830775	5.74636242	14.76038149
C	1.11762339	9.01463779	10.27034105
C	2.27627457	9.80038837	10.06873241
C	2.38975652	10.51035776	8.86718985
H	3.27263552	11.11547395	8.69452434
C	1.40765101	10.42866009	7.88380548
H	1.51892878	10.98108256	6.95708493
C	0.30377043	9.60463062	8.07723315
H	-0.43651466	9.50739631	7.29061741
C	0.13706627	8.87447736	9.26320673
C	-1.05285023	7.93067169	9.36482173
H	-1.05935563	7.48610279	10.36346466
C	-0.93461908	6.78124849	8.34625337
H	-1.87324908	6.22383360	8.29048645
H	-0.16993060	6.04781639	8.62287353
H	-0.69852550	7.15486064	7.34540880
C	-2.38876238	8.66495910	9.15655116
H	-2.49196519	9.00704441	8.12079645
H	-2.48049552	9.53979487	9.80669357

H	-3.22305982	7.98764545	9.36801039
C	3.43755757	9.83862688	11.04983084
H	3.15073100	9.26760584	11.93886999
C	3.79668883	11.27185091	11.47772636
H	4.19068000	11.84157959	10.62883483
H	4.57728829	11.24860553	12.24634230
H	2.93578488	11.81690263	11.87436593
C	4.67390955	9.15335975	10.44000692
H	5.45473596	9.04005539	11.20054835
H	5.08701620	9.75747167	9.62501014
H	4.44366131	8.16842399	10.02089648
C	5.17501727	5.75019075	5.68144846
H	6.25963064	5.75192661	5.85335174
H	4.99228780	6.17663803	4.68638609
H	4.84031786	4.70731004	5.67697723
C	2.45167982	6.83432216	6.62328474
H	1.91777290	7.50110883	7.31741283
H	2.30464825	7.25808958	5.62191162
C	4.94339270	8.52533409	6.85993069
H	4.33642834	9.20744145	7.46413580
H	4.89793546	8.85823633	5.81462591
H	5.98361242	8.60151075	7.19861746
C	7.34901017	6.64121697	9.23524035
H	7.67060811	6.64129439	8.18571729
H	8.18956827	6.28545878	9.84497192
H	7.13233002	7.67813508	9.51884496
C	6.30212802	3.77979302	9.00564050
H	5.44953462	3.10489513	9.15093866
H	7.14271983	3.41067225	9.60761561
H	6.58485747	3.72315883	7.94713080
C	5.39231061	5.55734475	11.30905588
H	5.22417908	6.58055759	11.66498251
H	6.20547755	5.11876260	11.90088454
C	-0.06769174	3.57151874	6.46132422
H	-1.10951439	3.43239567	6.77415820
H	0.04078547	3.15383009	5.45134012
H	0.12214247	4.65035346	6.39664707
C	2.84658654	2.85031894	6.87701936
H	3.26670608	3.85567538	7.00009535
H	2.82956026	2.62083663	5.80375942
H	3.54148319	2.15812471	7.36746506
C	0.62611369	0.88665797	7.74717927
H	0.28304197	0.63119352	8.75724004
H	1.46807096	0.22751709	7.50301445
H	-0.18624096	0.66322893	7.04477262
C	0.72847201	2.95688619	10.42234981
C	1.65860232	2.46906567	11.40141509
C	1.19655617	2.01650810	12.64269177
H	1.90983919	1.64850396	13.37342517

C	-0.16370582	1.99551482	12.95075707
H	-0.50408776	1.62351642	13.91197425
C	-1.07735068	2.45193676	12.00279773
H	-2.13949742	2.43225568	12.23388049
C	-0.65994770	2.94889828	10.76373888
C	-1.69326984	3.46888781	9.78286048
H	-1.13061852	3.93873854	8.97006677
C	-2.53240551	2.32742919	9.18538720
H	-3.10558167	1.81600555	9.96850798
H	-1.89315402	1.58566880	8.69932387
H	-3.24168715	2.71233542	8.44170038
C	-2.60559631	4.53387114	10.41109665
H	-3.32485237	4.91117370	9.67345801
H	-2.01847738	5.37912764	10.78476775
H	-3.17917989	4.12728167	11.25214683
C	3.12925418	2.32428177	11.04057568
H	3.39721433	3.14322993	10.35519198
C	3.35029763	1.02572211	10.24358178
H	3.10245089	0.15966776	10.86844576
H	4.39551599	0.93796988	9.92364851
H	2.71202471	1.00129236	9.35976925
C	4.08561886	2.36180721	12.23804268
H	3.89775143	3.21914005	12.89284407
H	5.12055822	2.42323441	11.88557211
H	3.99692937	1.45202839	12.84310045

4-Ba

Lowest frequency: 18.3484 cm^{-1}

Second frequency: 22.5738 cm^{-1}

Ba	11.43333972	10.00305616	20.72555404
Si	14.09768938	12.08240821	19.36537335
Si	13.25335670	12.55325965	22.32661283
Si	8.44935636	9.66051777	18.50297700
N	13.23119376	11.79297461	20.80210593
N	9.10599336	10.47808482	19.82968178
N	12.99484430	6.25101669	20.79022474
C	12.91902172	7.47362913	21.27870418
C	13.91913953	7.56730800	22.42677545
C	14.35211664	6.10430516	22.71843831
H	15.42236296	6.03096585	22.93238949
H	13.81180910	5.72904038	23.59529068
C	13.96621349	5.27610779	21.47920569
C	15.08787722	8.44717181	21.93135431
H	14.73613935	9.44522635	21.64790628
H	15.58645115	8.00603743	21.06246775
H	15.82794558	8.55914815	22.73183137
C	13.27740622	8.22192624	23.65697443
H	13.97003272	8.18845742	24.50558837
H	12.35337062	7.70914317	23.94833519
H	13.05238910	9.27609416	23.46889960
C	15.15780006	4.96895891	20.56868511
H	14.82464795	4.52170596	19.62805408
H	15.81188838	4.24910906	21.07159003
H	15.74713603	5.86165501	20.34870147
C	13.25905965	3.96957353	21.83525810
H	12.43555624	4.13646592	22.53234572
H	13.97654187	3.29602363	22.31548862
H	12.87108800	3.47174470	20.94130114
C	12.22655313	5.81272347	19.64622978
C	12.76070248	5.97772469	18.35189001
C	12.01985803	5.49064651	17.26779935
H	12.40816718	5.61381706	16.26297092
C	10.78371421	4.87950411	17.45723195
H	10.22449811	4.50846691	16.60486017
C	10.24740535	4.78409290	18.73623653
H	9.25920599	4.35944401	18.87247956
C	10.94836983	5.25483472	19.85190897
C	10.26113755	5.25722996	21.20691875
H	10.99305800	5.55835310	21.96289586
C	9.14331385	6.31318301	21.20805500
H	9.53836615	7.30852366	20.98605482
H	8.64606022	6.35128986	22.18219152
H	8.39030330	6.08575157	20.44657555
C	9.70174068	3.88048066	21.59544270
H	8.89268529	3.57705658	20.92202321
H	9.28885458	3.91412911	22.60979013
H	10.47380621	3.10600140	21.56189275
C	14.02798160	6.77288844	18.08540646
H	14.54366123	6.92682007	19.03587923
C	13.64848055	8.16464381	17.55760126
H	13.06784579	8.09455815	16.63179926
H	14.53846514	8.77217168	17.36947266
H	13.03522196	8.68854273	18.29454413
C	14.99638793	6.06954350	17.12481603
H	15.92485042	6.64491819	17.04296474
H	14.57250589	5.98656920	16.11806071
H	15.24636920	5.06050252	17.46763166
C	12.99032698	11.69397232	17.86475209
H	12.48575611	10.71921830	17.91144388
H	12.21171045	12.45817078	17.76721128
H	13.58258952	11.69112534	16.94026647
C	14.68855832	13.86176803	19.12491145
H	13.84620281	14.56118584	19.17628985
H	15.17959412	13.98604986	18.15081435
H	15.40857991	14.14244338	19.90399049
C	15.63425130	10.97916599	19.21663671
H	16.32707027	11.18971985	20.04162520
H	16.16635395	11.14585428	18.27060918

H	15.36186848	9.91841141	19.27589895
C	13.07197115	14.43357406	22.30849576
H	13.87419566	14.91006901	21.73429632
H	13.09953403	14.83384907	23.33061230
H	12.11551492	14.71893940	21.85656759
C	14.80374227	12.15474044	23.34175159
H	14.90141326	11.07501459	23.50859790
H	14.78611347	12.65266681	24.32010232
H	15.70093811	12.48985469	22.80513612
C	11.75810368	11.95497429	23.35339936
H	10.80074324	12.22702823	22.88704075
H	11.78530260	12.47786893	24.31839959
H	11.74224667	10.88340480	23.60393727
C	6.90581041	8.63579429	18.89948708
H	7.09601656	7.91691573	19.70443288
H	6.55593646	8.08259271	18.01783666
H	6.09216514	9.29474469	19.22853166
C	9.81539377	8.48256514	17.90901898
H	10.66562720	9.04771046	17.50265896
H	9.44439882	7.84236143	17.09958925
H	10.18187251	7.80516466	18.69250289
C	7.99408025	10.71918221	17.00205490
H	7.17577246	11.40946075	17.23808758
H	7.66970079	10.08633824	16.16491469
H	8.85016516	11.31733930	16.66713703
C	8.71984711	11.44102512	20.75929592
C	8.95202066	12.83676715	20.52754137
C	8.70035656	13.76616311	21.54046880
H	8.88405065	14.81924320	21.34522182
C	8.22035707	13.37756524	22.79205343
H	8.03399376	14.11633150	23.56483526
C	7.98771038	12.02551107	23.03205246
H	7.61287546	11.71212622	24.00358040
C	8.22973246	11.05686789	22.05121587
C	7.95334159	9.59690733	22.36416266
H	8.22702810	9.03719143	21.46333905
C	8.80642755	9.08855824	23.53961673
H	9.88226490	9.16184235	23.32808268
H	8.58233982	8.03983704	23.76684470
H	8.62289321	9.67825643	24.44481209
C	6.46115033	9.34669545	22.63379922
H	6.12667777	9.88096121	23.53133781
H	6.26515657	8.27725686	22.78179416
H	5.85722415	9.69388187	21.79023046
C	9.44366977	13.31862326	19.17592181
H	9.70315692	12.41996143	18.60446213
C	8.32607981	14.06051500	18.42283480
H	7.42675355	13.44181831	18.34760945
H	8.64933673	14.32625363	17.40899517
H	8.05567357	14.98508470	18.94808296
C	10.69890118	14.19587510	19.29095288
H	10.49181454	15.11948609	19.84489986
H	11.05986261	14.48492547	18.29621067
H	11.50094719	13.66148360	19.80974809

7

Lowest frequency: 20.5522 cm^{-1}

Second frequency: 24.9454 cm^{-1}

Ca	12.59392046	6.80110480	4.99308342
Si	10.67363783	4.87253757	3.24883569
Si	15.85279087	5.83057664	5.15792527
N	12.24095400	10.29086625	5.07257635
N	10.53269533	5.93315770	4.59581574
N	14.36413182	5.87882824	5.99407643
C	12.60426154	10.40035154	6.47199889
C	12.25672698	9.71318435	2.85038380
C	12.54683036	9.27514698	4.28732399
C	11.73368114	9.95961653	7.48480636
C	12.06597715	10.26436336	8.81152252
H	11.40193284	9.93855052	9.60440758
C	14.06098305	5.37335458	7.27642414
C	8.39170980	7.07993893	5.04233558
C	11.49585809	11.46933123	4.41835336
C	9.32659582	6.05065542	5.32717387
C	7.86110832	5.34004520	7.15140570
H	7.67732009	4.67164675	7.98999897
C	13.56323406	4.05344340	7.44937667
C	10.55740225	9.03519151	7.23519134
H	10.42766376	8.90396063	6.15452228
C	14.91836651	11.21920391	5.72384656
H	14.43870168	11.19494563	4.74423982
C	13.84817887	10.99993836	6.77927614
C	9.04750763	5.18829159	6.42537657
C	13.25988141	4.43274072	9.84894790
C	14.13954899	11.26975796	8.12005052
H	15.08874058	11.72678062	8.37521371
C	14.14250815	6.21376727	8.42013355
C	9.64633619	5.35751985	1.73397212
H	8.58260811	5.39448214	2.00190761
H	9.76352006	4.61622905	0.93264472
H	9.93076018	6.33707166	1.33390911
C	13.61027681	10.15403165	2.24015868
H	14.03222380	11.02342394	2.75038094
H	13.45470452	10.41662475	1.18758933
H	14.34171571	9.34182009	2.28816606
C	8.67741958	8.07996154	3.95932904
H	9.65396381	8.55189250	4.12164698
H	7.91307110	8.86407913	3.93597299

H	8.71999740	7.61400086	2.96966944
C	7.21640290	7.19875759	5.79464878
H	6.51710083	7.99603064	5.54930200
C	11.28413152	10.90979657	2.99767350
H	11.45798033	11.67384263	2.23412627
H	10.25318058	10.55585726	2.88957513
C	12.31919042	12.75795202	4.45886291
H	12.59192959	13.01256164	5.48710330
H	11.71186190	13.57608910	4.05813369
H	13.22899555	12.69132168	3.85939637
C	6.91930062	6.32994935	6.84845643
C	15.61054104	6.89024508	3.59816177
H	15.02763172	6.35926831	2.83464480
H	16.58811732	7.11998680	3.15582956
H	15.12350477	7.84874770	3.82035000
C	12.50364949	4.91217947	2.72157915
H	12.82497248	5.87276942	2.29713155
H	12.62723863	4.17527613	1.91813547
H	13.20272016	4.61118384	3.51586596
C	13.23794828	10.93993501	9.12961620
H	13.47085596	11.16920719	10.16420863
C	13.17954685	3.61146537	8.71926443
H	12.79177269	2.60019309	8.82382938
C	11.68998978	8.58449590	1.98577356
H	12.46936939	7.85048213	1.75600570
H	11.32751591	8.98518643	1.03241138
H	10.86804083	8.06537693	2.48091839
C	10.17541025	11.68543605	5.15581509
H	9.57607717	10.77253386	5.16148161
H	9.60456479	12.46764274	4.64421137
H	10.34551227	12.00492608	6.18805137
C	9.22945179	9.54765416	7.81108525
H	9.27818494	9.61801844	8.90373028
H	8.43027275	8.84650203	7.55265969
H	8.96798398	10.53550907	7.42159228
C	10.88457741	7.66064752	7.85011700
H	11.84652872	7.25752960	7.51062032
H	10.09483788	6.94523025	7.61882346
H	10.97950445	7.73432190	8.93838250
C	10.24593212	3.05894930	3.56331340
H	10.97322783	2.57760093	4.22652208
H	10.22756755	2.50212880	2.61731264
H	9.25414605	2.97295749	4.02401475
C	14.63481143	7.62673757	8.28479409
H	15.68795750	7.66050009	7.98848796
H	14.52328225	8.17310589	9.22533145
H	14.08580819	8.17767131	7.51224769
C	10.06619807	4.18128634	6.87530641
H	10.26024774	3.41669256	6.12021852
H	9.74174188	3.68102768	7.79291378
H	11.02969596	4.65975870	7.08370657
C	13.74085378	5.73374884	9.67129844
H	13.80406723	6.39904302	10.53041511
C	5.63373709	6.44969555	7.62570446
H	4.86945110	5.75663819	7.24838976
H	5.21868890	7.46114050	7.55680680
H	5.78411646	6.21873889	8.68637972
C	16.40203658	4.12800313	4.53615246
H	16.60729364	3.44969341	5.37370506
H	17.32152449	4.21332430	3.94129521
H	15.63112307	3.66756043	3.90619517
C	15.90734359	10.04027516	5.76798508
H	16.43818696	10.01932239	6.72638111
H	16.64694834	10.12397269	4.96384105
H	15.38203902	9.08797545	5.65936954
C	13.40848427	3.14616315	6.26150488
H	12.77824057	3.60989306	5.49403652
H	12.94353851	2.19890906	6.55223555
H	14.36973903	2.92769765	5.78746742
C	15.65055261	12.56041455	5.85414841
H	14.95185597	13.40324378	5.86352671
H	16.33855976	12.69292858	5.01184314
H	16.24813713	12.60610715	6.77105669
C	17.32636715	6.50819991	6.13195377
H	17.24157211	7.58659106	6.30321590
H	18.26869330	6.31852897	5.60166969
H	17.38427269	6.01379161	7.11015771
C	12.85602651	3.92662305	11.21004369
H	11.99743176	3.24865428	11.14601557
H	12.58376116	4.75189585	11.87691149
H	13.67241428	3.37202135	11.69290047

8

Lowest frequency: 17.6858 cm^{-1}

Second frequency: 26.9063 cm^{-1}

Ca	3.98741193	7.09869573	13.22564808
Si	6.64681995	8.22531229	12.00710290
Si	4.38994584	9.98857682	11.04601721
Si	3.18110008	9.23981742	15.76216593
Si	4.18830491	6.45169494	16.50675685
N	4.98454022	8.61556956	11.88753133
N	3.66326129	7.63186631	15.38627482
N	2.25938190	4.14581834	11.81525429
C	6.76273353	6.50478851	12.83657617
H	6.51637453	6.53024268	13.90840358
H	7.80501670	6.16797153	12.78440295
H	6.16782754	5.72880522	12.32950303
C	7.53866015	8.08239162	10.34639520

H	7.02926503	7.37216384	9.68413596
H	8.57679722	7.75088938	10.47729905
H	7.55875306	9.05554403	9.83986335
C	7.66395884	9.35507873	13.12355498
H	7.72713176	10.36953935	12.71504178
H	8.68559442	8.97378271	13.25181505
H	7.19264141	9.42386584	14.11107651
C	4.30956996	9.72357337	9.16931645
H	5.29877620	9.45527638	8.77864812
H	3.98313944	10.64244976	8.66445940
H	3.61552215	8.92195953	8.89413661
C	2.64801293	10.43679963	11.63113278
H	1.94802640	9.59177289	11.59596795
H	2.23027806	11.23248060	11.00074795
H	2.67822770	10.80385856	12.66247077
C	5.44653281	11.53907600	11.29543216
H	5.55540463	11.76996154	12.36149994
H	4.98737808	12.40565333	10.80193019
H	6.45108924	11.40817390	10.87297086
C	1.48396056	9.60803344	14.99841420
H	1.26239897	10.68283456	15.01315930
H	1.41044523	9.26935847	13.95788535
H	0.70589399	9.09129186	15.57372591
C	4.43150160	10.48445871	15.08809937
H	5.35703232	10.43000881	15.67606387
H	4.05984152	11.51640493	15.13038704
H	4.68875020	10.24455340	14.05008788
C	2.97811534	9.60698393	17.60932045
H	2.17978009	9.00017590	18.05284052
H	2.71396866	10.66365734	17.74897440
H	3.90154017	9.41714005	18.16863001
C	5.74089401	6.95220840	17.46655303
H	6.53276643	7.24935942	16.76654012
H	6.11833195	6.12513035	18.08244090
H	5.54623917	7.80470115	18.12778954
C	2.88397620	5.90033643	17.76204672
H	2.50546373	6.74565581	18.34698602
H	3.30454890	5.16422274	18.46006231
H	2.03645546	5.43426754	17.24654659
C	4.65907513	4.88182894	15.55148094
H	3.90759717	4.61235566	14.79745200
H	4.71036148	4.03110177	16.24163539
H	5.64001684	4.95934836	15.06820966
C	2.89532435	5.28984228	11.65072266
C	2.81079661	5.65962577	10.17322279
C	2.20937847	4.41136022	9.47343409
H	1.49697506	4.68278317	8.68913165
H	3.01406628	3.83179638	9.00658223
C	1.54313992	3.57444987	10.57598579
C	1.90749859	6.90272446	10.04815366
H	0.90843766	6.73194552	10.46190454
H	1.80022220	7.17639265	8.99313583
H	2.35704416	7.75415298	10.56491932
C	4.21454632	5.99260844	9.64339921
H	4.61975313	6.87127043	10.15751565
H	4.16479492	6.21133549	8.57045847
H	4.89810809	5.14797164	9.79034003
C	0.03962286	3.84086584	10.69902437
H	-0.46034397	3.47778261	9.79528435
H	-0.18403494	4.90377803	10.80643200
H	-0.37775746	3.30503909	11.55446537
C	1.75723497	2.07246823	10.41123259
H	2.79549011	1.82714956	10.18479743
H	1.14185900	1.72061072	9.57674034
H	1.45020332	1.52811078	11.30943662
C	2.17085161	3.48304761	13.09911048
C	1.20163474	3.89389948	14.03809153
C	1.17059179	3.23718159	15.27600942
H	0.44292154	3.54709521	16.01783972
C	2.06462620	2.21367895	15.57398246
H	2.02481442	1.72282605	16.54052941
C	3.03013964	1.84259719	14.64186309
H	3.75080549	1.07242625	14.89374495
C	3.11397822	2.47476233	13.39606586
C	0.23133436	5.03790511	13.79586165
H	0.39931986	5.41713463	12.78388430
C	-1.23381280	4.57979616	13.90669481
H	-1.45506039	3.72680379	13.25863779
H	-1.90615019	5.40179151	13.63721722
H	-1.46801763	4.28413885	14.93549492
C	0.47995324	6.20100241	14.76978036
H	0.22197579	5.91347744	15.79533185
H	-0.14750389	7.05642277	14.49808304
H	1.52326972	6.53539999	14.79137291
C	4.26142604	2.11646882	12.46291502
H	4.09250375	2.61909510	11.50635237
C	4.35347201	0.60367383	12.20674428
H	5.11692523	0.39559143	11.44879619
H	3.40139373	0.18998122	11.86155958
H	4.64114047	0.06676350	13.11731111
C	5.59791132	2.64692605	13.00868769
H	5.81514990	2.22698244	13.99659663
H	5.57710365	3.73341816	13.11012446
H	6.41667288	2.37772638	12.33163561

Monovalent

3-Ca⁻

Lowest frequency: 22.6500 cm^{-1}
Second frequency: 26.4922 cm^{-1}

Ca	0.65751	0.16002	-0.50761
Si	0.93162	2.54307	1.70160
N	1.22220	2.32428	0.04164
C	-1.46718	-0.65952	-1.32890
Si	1.80383	3.35186	-1.17321
N	2.41984	-1.30310	-0.77224
C	-1.70368	-1.18699	-2.76370
Si	3.25748	-2.01144	-2.06613
N	-2.70506	-0.86136	-0.64542
C	-3.22680	-1.46453	-2.83860
H	-3.73792	-0.57479	-3.22748
H	-3.47463	-2.30546	-3.49632
C	-3.69461	-1.70190	-1.38696
C	-0.86685	-2.45291	-3.04091
H	0.19107	-2.23827	-2.86293
H	-0.98461	-2.79133	-4.08130
H	-1.14363	-3.27473	-2.37574
C	-1.30262	-0.12647	-3.80767
H	-1.85727	0.80453	-3.64783
H	-1.49401	-0.47837	-4.83281
H	-0.23449	0.10710	-3.72895
C	-3.58852	-3.19664	-1.02029
H	-2.54131	-3.49812	-0.94443
H	-4.07365	-3.81096	-1.78946
H	-4.07736	-3.40776	-0.06596
C	-5.12959	-1.22879	-1.15563
H	-5.25627	-0.19645	-1.49107
H	-5.38966	-1.27518	-0.09247
H	-5.82987	-1.86429	-1.71103
C	-3.03743	-0.05384	0.47714
C	-3.09675	-0.60671	1.78088
C	-3.41755	0.22111	2.86666
H	-3.45962	-0.20837	3.86379
C	-3.64263	1.58204	2.69443
H	-3.87027	2.21442	3.54742
C	-3.56094	2.13132	1.41354
H	-3.73813	3.19396	1.27387
C	-3.26815	1.33794	0.30018
C	-2.77912	-2.06607	2.04040
H	-2.43624	-2.48457	1.09025
C	-1.63871	-2.23318	3.05629
H	-0.73217	-1.72637	2.71994
H	-1.40433	-3.29656	3.19025
H	-1.91025	-1.82356	4.03707
C	-4.02536	-2.83486	2.51221
H	-4.35038	-2.47176	3.49569
H	-3.81076	-3.90736	2.60223
H	-4.86417	-2.70606	1.81979
C	-3.24369	1.98306	-1.07376
H	-3.01474	1.19042	-1.79058
C	-4.60513	2.60339	-1.43344
H	-4.58608	2.98966	-2.46037
H	-4.84407	3.44044	-0.76570
H	-5.41761	1.87355	-1.35388
C	-2.13642	3.04059	-1.18229
H	-2.04866	3.39975	-2.21542
H	-1.16736	2.63942	-0.87090
H	-2.35495	3.90683	-0.54468
C	-0.31643	3.91022	2.10908
H	0.03804	4.88697	1.75730
H	-0.49334	3.97483	3.19144
H	-1.27190	3.69500	1.61977
C	0.19572	0.92984	2.37907
H	-0.74337	0.66136	1.87337
H	-0.06572	1.05046	3.43754
H	0.90402	0.08997	2.33104
C	2.48365	2.91193	2.72521
H	3.20417	2.09487	2.61516
H	2.24408	3.02186	3.79169
H	2.96200	3.83904	2.38260
C	1.06053	5.09891	-1.13622
H	-0.03448	5.04982	-1.13921
H	1.38624	5.69307	-2.00105
H	1.37093	5.62651	-0.22473
C	3.68751	3.59060	-1.16190
H	4.02520	3.93326	-0.17548
H	4.00394	4.32872	-1.91197
H	4.19611	2.64279	-1.37665
C	1.37895	2.60150	-2.86347
H	1.83377	1.60785	-2.97797
H	1.75801	3.22432	-3.68458
H	0.29369	2.49980	-2.98589
C	2.90633	-1.01015	-3.62962
H	1.83041	-0.92185	-3.82120
H	3.36404	-1.48631	-4.50682
H	3.32116	0.00057	-3.53394
C	5.14595	-2.06123	-1.84500
H	5.58579	-1.06254	-1.95692
H	5.61172	-2.72496	-2.58631
H	5.40175	-2.43358	-0.84432
C	2.79663	-3.81785	-2.45166
H	3.08396	-4.47289	-1.61836
H	3.31949	-4.16914	-3.35254
H	1.71846	-3.92737	-2.61420

C	2.58182	-1.53224	0.60182
C	3.46987	-0.72588	1.37707
C	3.54017	-0.89364	2.76200
H	4.21688	-0.25789	3.33040
C	2.75342	-1.83372	3.43950
C	1.88021	-2.61800	2.68050
H	1.25082	-3.34958	3.18337
C	1.77040	-2.48120	1.29088
C	4.27296	0.34593	0.69938
H	4.85007	-0.05137	-0.13858
H	4.95868	0.82889	1.40405
H	3.60441	1.11419	0.29092
C	2.80898	-1.95582	4.94090
H	3.84087	-1.91583	5.31116
H	2.36549	-2.89926	5.27840
H	2.25750	-1.14047	5.42970
C	0.77659	-3.31078	0.52343
H	0.05351	-2.68087	-0.01748
H	0.21869	-3.96925	1.19762
H	1.26915	-3.92164	-0.23893

3-Sr

Lowest frequency: 17.2807 cm^{-1}
Second frequency: 23.3071 cm^{-1}

Sr	8.25274050	9.15726296	7.51548224
Si	8.45692322	10.50388444	10.69197858
Si	6.32167704	11.73839278	8.74180802
Si	7.77681346	5.23935342	8.13452225
N	7.33594557	6.82618204	7.72084714
N	7.55933939	10.71764564	9.27915518
C	6.08770471	7.25062574	7.25406794
C	5.11505453	7.79573065	8.14913024
N	10.74166568	9.92845741	4.91344301
C	5.77771760	7.24803108	5.86055114
C	10.39426510	8.71205651	4.26664269
C	3.92622794	8.33319190	7.64731728
H	3.20308309	8.74649515	8.34803701
C	10.12775980	10.28045575	6.15019991
C	6.00704984	11.37695900	6.89955167
H	6.92293310	11.50471102	6.30131552
H	5.26266566	12.07477001	6.49386460
H	5.60222374	10.36690177	6.74314778
C	4.58493900	7.81812389	5.40149432
H	4.38582971	7.82374090	4.33092599
C	3.64584481	8.37652776	6.27532572
C	6.73883932	6.60362432	4.90452145
H	7.76435075	6.96123728	5.04597287
H	6.45249717	6.78634864	3.86523150
H	6.78264488	5.51981206	5.06346214
C	9.40320819	8.70859929	3.25309382
C	5.39974904	7.81224468	9.62417970
H	5.82253182	6.85977294	9.95420982
H	4.49049098	8.02323500	10.19782535
H	6.13485885	8.59235032	9.86466832
C	10.98560803	7.49145309	4.67715443
C	8.57491197	9.94841089	2.96061306
H	9.00705319	10.76128260	3.55217354
C	10.66313535	6.31213026	3.99298508
H	11.11813555	5.37552093	4.30237280
C	9.12485646	7.51203237	2.57793268
H	8.37437866	7.50699709	1.79237826
C	11.25151965	6.66586086	7.03543671
H	10.22401010	7.00228783	7.19993458
H	11.81541479	6.80966832	7.96464445
H	11.20568671	5.59119357	6.82620839
C	11.90861973	7.43815171	5.88094055
H	12.03891857	8.46913833	6.21809769
C	4.66893971	11.49858796	9.63928819
H	4.33561775	10.46098340	9.53905112
H	3.88777157	12.15512611	9.23211725
H	4.78120415	11.71780937	10.70910721
C	7.12698485	9.76018857	3.44523200
H	6.55728649	10.68945245	3.32482883
H	7.09265242	9.48352703	4.50125725
H	6.61884574	8.96919173	2.88097241
C	9.75911588	6.32327029	2.93067688
H	9.52294455	5.40180178	2.40661062
C	10.86976637	11.55946689	6.58865903
C	6.71069487	13.59162924	8.88537894
H	6.86651466	13.86207432	9.93830344
H	5.88833512	14.20602720	8.49333533
H	7.62488291	13.84238893	8.33598918
C	2.39572797	9.04205573	5.75960889
H	1.54480971	8.87291105	6.43044329
H	2.12370518	8.66530195	4.76688812
H	2.52831689	10.12977813	5.67247446
C	8.57970907	10.33315545	1.47292742
H	8.08516107	9.56316932	0.86743421
H	9.59707715	10.45810616	1.09075037
H	8.03459284	11.27295234	1.32039734
C	9.89011990	12.61593583	7.12022536
H	9.17235684	12.89514810	6.34017031
H	10.41604821	13.52212657	7.45595826
H	9.32157668	12.21323152	7.96622441
C	11.80819375	10.83856624	4.40130401
C	8.56546510	4.21131629	6.73415790
H	7.78693799	3.73193459	6.12582674

H 9.21427154 3.41975553 7.13491140
H 9.15918244 4.84793003 6.06982191
C 11.59124086 12.06575252 5.30936986
H 10.93415385 12.77691588 4.79191252
H 12.53424340 12.58223856 5.52812983
C 9.02143253 5.26442940 9.55969474
H 9.90025454 5.87457825 9.32397766
H 9.36607911 4.24927951 9.79874056
H 8.55026919 5.68881571 10.45505397
C 6.28385839 4.21280941 8.70632578
H 5.89887765 4.56933122 9.66956390
H 6.54406458 3.15065247 8.81076918
H 5.47207979 4.29789137 7.97198006
C 11.62535152 11.18872923 2.91885855
H 11.62336748 10.28167116 2.30380445
H 12.44948635 11.82889257 2.58041787
H 10.68838701 11.72518250 2.75486202
C 13.28051223 6.82781624 5.55714255
H 13.18271133 5.77631882 5.25957673
H 13.92992056 6.86379072 6.44073441
H 13.77901609 7.36273342 4.74336847
C 13.21347002 10.22841656 4.56017896
H 13.42925898 9.97804582 5.60037326
H 13.97603550 10.93672401 4.21182168
H 13.29481737 9.31662342 3.95893732
C 11.88399108 11.24271197 7.71196792
H 11.34982824 10.91263724 8.60898400
H 12.48007983 12.13055911 7.97500357
H 12.56806793 10.43960506 7.42054816
C 7.42175405 10.12572675 12.23789985
H 6.74406505 10.96255628 12.45250181
H 8.05272764 9.95930474 13.12177906
H 6.80691522 9.23185044 12.07715697
C 9.56382608 11.97612476 11.14792252
H 10.22912159 12.22919103 10.31411010
H 10.17934506 11.76642743 12.03351673
H 8.94668661 12.85922046 11.35913430
C 9.59963059 8.99998778 10.43887221
H 9.01287721 8.09423895 10.22946137
H 10.18915870 8.80002948 11.34291581
H 10.32101337 9.15315951 9.62072020

3-Ba^{-}

Lowest frequency: 19.3868 cm^{-1}
Second frequency: 21.3883 cm^{-1}
Ba -0.5400880 -0.0300640 -0.5200170
N 3.1057890 -1.0290440 -0.3862550
C 1.8270770 -1.3080850 -0.9507620
Si -0.8857750 3.1509210 1.1261130
N -1.0053480 2.5632370 -0.4562310
C 2.0717240 -2.5407600 -1.8397850
Si -1.3813320 3.2575440 -1.9483060
N -2.6674740 -1.5830340 -0.5376850
C 3.6065370 -2.5583840 -2.0800310
H 4.0056760 -3.5706790 -2.2221170
H 3.8282320 -1.9817730 -2.9878270
Si -3.3588910 -2.4534860 -1.8019600
C 4.2548360 -1.8511190 -0.8705210
C 1.3108310 -2.4286950 -3.1706790
H 1.5029200 -3.2937480 -3.8222850
H 0.2292150 -2.3881710 -2.9880850
H 1.6002820 -1.5134460 -3.6993530
C 1.5988950 -3.8294390 -1.1288630
H 0.5123650 -3.7979730 -0.9941720
H 1.8490690 -4.7279190 -1.7144290
H 2.0474110 -3.9271160 -0.1357950
C 5.4524880 -0.9927050 -1.2935890
H 5.1803720 -0.3216060 -2.1106910
H 5.8140000 -0.3928080 -0.4507120
H 6.2754840 -1.6334100 -1.6341720
C 4.7531620 -2.8467610 0.1936460
H 5.1186310 -2.3035790 1.0713140
H 3.9633880 -3.5300030 0.5116190
H 5.5821500 -3.4441990 -0.2068370
C 3.2227000 -0.0299830 0.6222570
C 2.9696710 -0.3533760 1.9803140
C 3.1131130 0.6389540 2.9592490
H 2.9236390 0.3905960 3.9995810
C 3.4663490 1.9425680 2.6161700
H 3.5668250 2.7027050 3.3850900
C 3.6450190 2.2740840 1.2781990
H 3.8683610 3.3010230 1.0059000
C 3.5072230 1.3138040 0.2668470
C 2.4893040 -1.7326890 2.3950050
H 2.4839960 -2.3488570 1.4928300
C 3.4026090 -2.3789630 3.4490440
H 3.3673720 -1.8226270 4.3939790
H 3.0770140 -3.4059530 3.6556370
H 4.4445710 -2.4086930 3.1166300
C 1.0453780 -1.6791750 2.9179210
H 0.7055970 -2.6784310 3.2120930
H 0.9510190 -1.0135090 3.7834650
H 0.3572820 -1.3189790 2.1507880
C 3.5875420 1.7589810 -1.1826670
H 3.4286950 0.8658700 -1.7958470
C 2.4626390 2.7560250 -1.5088990
H 1.4800370 2.4024560 -1.1831030

H	2.6321620	3.7171580	-1.0073610
H	2.4213880	2.9436410	-2.5889540
C	4.9445590	2.3961850	-1.5289050
H	4.9864790	2.6451350	-2.5967480
H	5.0861740	3.3276350	-0.9662360
H	5.7817500	1.7332580	-1.2949920
C	-2.5099950	3.8546340	1.8111520
H	-3.2972170	3.0950910	1.7439750
H	-2.4124970	4.1629200	2.8611350
H	-2.8298760	4.7262880	1.2247990
C	-0.3833630	1.7379570	2.2967610
H	-1.1446500	0.9469230	2.3564910
H	0.5912230	1.3047080	2.0307130
H	-0.2677300	2.1333870	3.3145940
C	0.4322260	4.4961530	1.3662250
H	1.4179830	4.0928450	1.1080250
H	0.2357030	5.3587260	0.7170970
H	0.4648950	4.8455620	2.4076140
C	-3.2231890	3.6520790	-2.1943560
H	-3.8227520	2.7349970	-2.1390480
H	-3.5761100	4.3281370	-1.4050430
H	-3.4099320	4.1282120	-3.1669860
C	-0.9409620	2.0234180	-3.3340400
H	0.1107500	1.7078490	-3.2785370
H	-1.5810460	1.1295310	-3.2993490
H	-1.0946550	2.4794410	-4.3210250
C	-0.4588720	4.8730230	-2.3307260
H	-0.7642560	5.6531220	-1.6207210
H	0.6231880	4.7343560	-2.2293290
H	-0.6720280	5.2335060	-3.3465190
C	-5.2458940	-2.6136340	-1.6623880
H	-5.5149190	-3.0421460	-0.6880910
H	-5.7234670	-1.6285660	-1.7358290
H	-5.6604360	-3.2588680	-2.4487640
C	-2.7002940	-4.2201530	-2.0522350
H	-1.6032580	-4.2183060	-2.0741540
H	-3.0182470	-4.8824960	-1.2371540
H	-3.0614780	-4.6469930	-2.9985260
C	-2.9715660	-1.5398890	-3.4171320
H	-3.4016250	-0.5306860	-3.3884710
H	-1.8883310	-1.4441210	-3.5744450
H	-3.3831340	-2.0669100	-4.2881720
C	-2.9357490	-1.3703880	0.8045390
C	-2.4044050	-2.2277230	1.8164160
C	-2.5417500	-1.8965610	3.1682600
H	-2.1088560	-2.5601380	3.9154570
C	-3.2035810	-0.7366990	3.5866270
C	-3.7574800	0.0869350	2.5985170
H	-4.2838720	0.9920880	2.8967360
C	-3.6372020	-0.1988400	1.2354060
C	-1.6944670	-3.4853140	1.4039580
H	-2.3631890	-4.1504530	0.8454950
H	-0.8554690	-3.2705380	0.7313270
H	-1.3105140	-4.0272020	2.2749530
C	-3.2760580	-0.3599870	5.0441910
H	-4.2294410	0.1243260	5.2875460
H	-3.1700830	-1.2398840	5.6893550
H	-2.4766310	0.3437970	5.3164240
C	-4.2072220	0.7399150	0.2087440
H	-4.6447590	0.1857110	-0.6259640
H	-4.9699130	1.3898820	0.6518090
H	-3.4278050	1.3932710	-0.2095770

4-Ca⁻

Lowest frequency: 24.4800 cm^{-1}
Second frequency: 28.0070 cm^{-1}

Ca	2.40332942	17.19414280	9.78203529
Si	4.29871634	16.03460972	7.21462994
Si	5.84933994	17.40557007	9.44164117
Si	1.32913517	19.99717521	7.86547695
N	0.86577300	14.58553201	11.42727037
N	4.42383544	16.80822616	8.72659079
N	1.44633708	19.27912312	9.41918951
C	1.47865280	15.86609391	11.58444633
C	1.44040138	16.13880435	13.10766675
C	0.89804943	14.82697225	13.74244523
H	1.73978958	14.20645674	14.07569265
H	0.26071848	15.01717877	14.61351189
C	0.14404475	14.07401791	12.62759085
C	0.25564246	12.55506039	12.76581158
H	-0.15465855	12.05197433	11.88275413
H	-0.30521667	12.21745155	13.64558283
H	1.29678897	12.24923954	12.88433728
C	-1.34909330	14.45527477	12.61335206
H	-1.47534532	15.52070415	12.41310425
H	-1.80636843	14.22866361	13.58521746
H	-1.88821165	13.89105667	11.84594573
C	2.86123819	16.42629349	13.63013165
H	3.24765869	17.34998682	13.18556545
H	3.54537141	15.61463962	13.35967016
H	2.86925168	16.54956850	14.72398436
C	0.56096556	17.35289312	13.46407964
H	0.61466107	17.57208294	14.54095391
H	-0.48676543	17.18715665	13.20319038
H	0.89434271	18.24032760	12.91793702
C	1.12281790	13.80795425	10.26504394
C	0.20161419	13.79140270	9.19017413

C	0.47300986	13.00075336	8.06270295
H	-0.23495019	12.99186001	7.23848308
C	1.64218623	12.25297675	7.97539309
H	1.84379435	11.65092428	7.09436632
C	2.56779960	12.30012475	9.01903387
H	3.48774176	11.72755407	8.94539066
C	2.33585440	13.07618525	10.16010883
C	3.38423280	13.11497717	11.25726160
H	2.96035353	13.70466923	12.07566025
C	3.73410873	11.70961019	11.77679972
H	4.40988830	11.78279154	12.63789732
H	4.24599830	11.12330382	11.00366843
H	2.84328999	11.15083289	12.08139970
C	4.66098808	13.82620372	10.78138377
H	5.34797852	13.97778475	11.62347929
H	4.43515532	14.79804219	10.33361245
H	5.18331379	13.22754561	10.02473523
C	-1.04965508	14.64721498	9.21255923
H	-1.06865873	15.14728382	10.18239174
C	-2.32559438	13.80324569	9.06801822
H	-3.21610462	14.43084440	9.19586676
H	-2.35629902	13.00266015	9.81531551
H	-2.38261181	13.33644002	8.07655273
C	-0.99720666	15.73985900	8.13262436
H	-1.93505042	16.30551704	8.10449345
H	-0.81929174	15.31639628	7.13735689
H	-0.19589410	16.45495447	8.34005898
C	2.45626948	15.85460754	6.81289207
H	1.94784683	15.22991591	7.56030561
H	2.30519161	15.36054908	5.84487421
H	1.96427410	16.83426274	6.75688443
C	5.02708003	14.28768132	7.13052113
H	4.49099141	13.62786095	7.81873800
H	6.08804994	14.28818568	7.40716712
H	4.93413843	13.87590966	6.11593991
C	5.11716347	16.97715928	5.78258074
H	4.71052829	17.98984980	5.68825426
H	4.97081103	16.45225942	4.82848182
H	6.19793942	17.06421507	5.95817704
C	7.38298811	16.33166411	9.12170870
H	7.21039367	15.31075647	9.48438880
H	8.26626262	16.73688878	9.63384237
H	7.60767962	16.27545294	8.04867176
C	5.64881484	17.48546937	11.32212756
H	4.77305601	18.07701413	11.61275705
H	6.52281441	17.95472000	11.79308134
H	5.52382534	16.48207120	11.74257762
C	6.28128717	19.16048306	8.87022902
H	6.43795120	19.18293674	7.78454659
H	7.18630737	19.54297026	9.36185765
H	5.44995590	19.83884263	9.09531779
C	0.12074974	19.13275396	6.67432182
H	0.29488222	18.05287571	6.60879608
H	0.23021031	19.55145499	5.66408377
H	-0.91771261	19.28300109	6.99395948
C	3.02319735	19.94726295	7.03497231
H	3.66668819	20.74426268	7.42687659
H	2.96191300	20.05384787	5.94411140
H	3.51957050	18.99797718	7.26954732
C	0.69992095	21.78516348	7.94654965
H	0.42268065	22.14240962	6.94601444
H	1.44552835	22.47204026	8.36145923
H	-0.18585794	21.83801972	8.59116634
C	0.91795902	19.86884814	10.58212641
C	1.78365938	20.51513814	11.52016871
C	1.25380531	21.10265258	12.67398006
H	1.92272840	21.59443996	13.37445961
C	-0.11352983	21.07057025	12.94682258
H	-0.50694963	21.53187703	13.84814738
C	-0.96187689	20.41974288	12.05340866
H	-2.02672267	20.36386912	12.26960106
C	-0.47364640	19.81618913	10.89084366
C	-1.42788973	19.04311891	10.00063153
H	-0.83554134	18.68425635	9.15410483
C	-2.57273686	19.90887606	9.45726439
H	-3.20151856	20.29369498	10.27037783
H	-3.21418783	19.32244104	8.78633394
H	-2.18483068	20.76702513	8.89808189
C	-1.95929714	17.80866284	10.74328925
H	-1.11607894	17.16638433	11.01912718
H	-2.65151329	17.23317063	10.11632714
H	-2.48799307	18.09552036	11.66104469
C	3.27473848	20.59551053	11.24378896
H	3.52372927	19.74150365	10.59862643
C	3.62333844	21.85774189	10.43701585
H	3.33967617	22.75691193	10.99859828
H	3.08696454	21.86306033	9.48675920
H	4.69952681	21.90195788	10.22713103
C	4.13735780	20.52880181	12.51014086
H	5.19394433	20.42260398	12.24030899
H	3.85546122	19.68077442	13.14319835
H	4.03977353	21.44316972	13.10895016

4-Sr⁻

Lowest frequency: 21.9456 cm^{-1}

Second frequency: 28.0799 cm^{-1}

Sr	2.25843518	5.63191239	9.60344066

H	1.98566896	5.94462227	6.57984955
H	4.59987605	4.74376076	11.46598080
Si	4.33060289	6.75470072	7.04900873
Si	5.82803135	5.40796034	9.36883220
Si	1.20401650	2.69121562	7.71945160
N	0.71653196	8.27326575	11.36703042
N	4.46077404	6.01270225	8.56702437
N	1.30440118	3.37635517	9.27706784
C	1.32854083	6.99877637	11.56765829
C	1.26224021	6.75907977	13.09157087
C	0.68772589	8.07821118	13.68889133
H	0.02518620	7.89843395	14.54438986
H	1.51478648	8.71273708	14.03613913
C	-0.04053982	8.80541343	12.53566959
C	-1.52951862	8.41097884	12.49164516
H	-2.05629094	8.95726137	11.70330208
H	-2.01194881	8.64720420	13.44759291
H	-1.63956518	7.34039236	12.30627818
C	0.06270562	10.32719374	12.64042239
H	1.10122711	10.63694854	12.77398689
H	-0.51828612	10.68673396	13.49823316
H	-0.32774204	10.80748374	11.73578753
C	0.39111050	5.53663090	13.44210989
H	0.42001981	5.32826641	14.52211128
H	0.75015553	4.64801967	12.91285681
H	-0.65203035	5.68481820	13.15199846
C	2.67665672	6.49740829	13.64729318
H	3.35334013	7.31863513	13.38638970
H	3.08758912	5.57773765	13.21550148
H	2.66364407	6.38225625	14.74204386
C	0.99049344	9.00589551	10.17939141
C	2.21501205	9.71743058	10.05568925
C	2.46876796	10.44636446	8.88822528
H	3.39643125	11.00461783	8.80354432
C	1.55463848	10.46508712	7.83298314
H	1.77147897	11.03250018	6.93290164
C	0.37927931	9.72927139	7.93393513
H	-0.31828503	9.71280721	7.10090885
C	0.08729289	8.98187732	9.08669971
C	-1.16614221	8.12843751	9.11396383
H	-1.19152200	7.63830242	10.08931280
C	-1.11516848	7.02740169	8.04156602
H	-2.05151345	6.45944247	8.01967830
H	-0.31505409	6.30952375	8.24711662
H	-0.94003340	7.44320975	7.04282392
C	-2.43952417	8.97385727	8.95103475
H	-2.48869892	9.42500881	7.95192837
H	-2.46999382	9.78656975	9.68466651
H	-3.33329891	8.35223175	9.08474149
C	3.25673692	9.70575936	11.15932840
H	2.83648463	9.11923861	11.98168779
C	3.58338740	11.12236982	11.66364369
H	4.08022904	11.71029468	10.88190173
H	4.26388011	11.07034683	12.52258541
H	2.68358219	11.66699010	11.96683278
C	4.54660649	9.01085131	10.69290660
H	5.22937367	8.86660061	11.53944028
H	5.06755007	9.61594406	9.94023796
H	4.34156253	8.03656440	10.24051699
C	5.13729422	5.78653406	5.62855920
H	6.21914673	5.70277313	5.79928565
H	4.98321507	6.28326449	4.66073245
H	4.72879851	4.77124377	5.56805202
C	2.48233350	6.92408269	6.62718652
H	1.95622609	7.57508247	7.34070304
H	2.35433001	7.39076093	5.64213032
C	5.04537340	8.50689465	6.95142231
H	4.51098897	9.16122713	7.64760695
H	4.94891301	8.92475417	5.93982787
H	6.10722257	8.50673568	7.22669524
C	7.38825189	6.46720375	9.14287050
H	7.67900446	6.50629048	8.08465798
H	8.23699372	6.06959478	9.71571403
H	7.19470436	7.49441929	9.47667937
C	6.27105732	3.64298242	8.83516981
H	5.41529775	2.97960775	9.01094164
H	7.13745770	3.24618618	9.38189807
H	6.49289732	3.61425002	7.76084516
C	5.49494300	5.33473602	11.23402024
H	5.34361747	6.33894859	11.64437222
H	6.33028697	4.86326597	11.76839062
C	0.02099845	3.61271516	6.54541080
H	-1.02033445	3.49857702	6.87144717
H	0.10659160	3.21275785	5.52541414
H	0.23872057	4.68690907	6.50077534
C	2.90783974	2.76284372	6.90406963
H	3.38227777	3.73286945	7.10058399
H	2.86101836	2.61249993	5.81736731
H	3.56756632	1.99903205	7.33358460
C	0.57253847	0.90188733	7.73877667
H	-0.29160958	0.82383396	8.41012575
H	1.33160362	0.19855759	8.09919289
H	0.26117669	0.58503299	6.73477926
C	0.80787619	2.84712878	10.47259461
C	1.69711387	2.24044500	11.41766834
C	1.19885011	1.72034441	12.61627677
H	1.88487042	1.25794494	13.32034991
C	-0.15938941	1.78161306	12.92966067

H	-0.52854303	1.37087114	13.86499945
C	-1.02861204	2.39990764	12.03292289
H	-2.08494762	2.48148470	12.28098816
C	-0.57185003	2.94068924	10.82774033
C	-1.54199908	3.68674911	9.93189167
H	-0.95890384	4.03210971	9.07226180
C	-2.67710321	2.79297712	9.41299416
H	-3.28762137	2.40831184	10.24005058
H	-2.27762308	1.93419274	8.86296595
H	-3.33762059	3.35613827	8.74042528
C	-2.08810666	4.92892534	10.65204925
H	-2.77911232	5.48947793	10.01019191
H	-1.25512829	5.58424944	10.93104377
H	-2.62458809	4.65240645	11.56833723
C	3.17568744	2.13147702	11.09084837
H	3.41235722	2.96662010	10.41624946
C	3.47447851	0.84693242	10.29934531
H	3.22243881	-0.03577911	10.90074758
H	4.53648145	0.79362125	10.02763629
H	2.88170380	0.81858521	9.38352571
C	4.08727901	2.21514025	12.32097830
H	3.83838241	3.07888828	12.94673082
H	5.13334694	2.30662395	12.00787407
H	4.00477566	1.31396146	12.94178836

4-Ba⁻

Lowest frequency: 18.0947 cm^{-1}
Second frequency: 22.1701 cm^{-1}

Ba	11.55238433	9.91096435	20.74835957
Si	14.07743988	12.19558832	19.37236802
Si	13.25897323	12.64436095	22.34177942
Si	8.50212687	9.65736838	18.52076963
N	13.23871538	11.91148708	20.81506580
N	9.13008029	10.45248354	19.86631291
N	12.90895423	6.20395050	20.78008316
C	12.84883292	7.55152625	21.24053473
C	13.95319002	7.61190929	22.32357096
C	14.19888119	6.13406154	22.72922686
H	15.23166230	5.94921214	23.05058742
H	13.53845555	5.88425463	23.57024943
C	13.81270563	5.26650496	21.51602642
C	15.23194435	8.26724170	21.75429605
H	15.03004148	9.31333416	21.49934697
H	15.56605655	7.76493883	20.84142169
H	16.05576120	8.24162286	22.48487890
C	13.51748616	8.42782737	23.55031569
H	14.27513860	8.39076358	24.34655240
H	12.56876835	8.04801193	23.94714418
H	13.38909818	9.48415538	23.28806772
C	15.03448726	4.85414285	20.67481658
H	14.70711276	4.34702044	19.76090302
H	15.66654546	4.15966973	21.24271039
H	15.64260542	5.71680834	20.39579025
C	13.09295303	3.98116381	21.94670632
H	12.25999708	4.20683969	22.61642733
H	13.78909099	3.31878218	22.47628008
H	12.70629173	3.44259985	21.07414125
C	12.17675946	5.78552614	19.63527449
C	12.72306898	5.94850500	18.33835260
C	12.01103723	5.47219849	17.23077848
H	12.43072215	5.59344326	16.23623689
C	10.76630761	4.86404381	17.38369176
H	10.22714908	4.49604785	16.51547467
C	10.20067884	4.76912017	18.65031291
H	9.20533951	4.34942938	18.76334882
C	10.87825406	5.23989547	19.78222664
C	10.16201096	5.25910896	21.12058449
H	10.88267062	5.61672098	21.86269097
C	9.01256230	6.27789244	21.07496629
H	9.39103050	7.27180019	20.82466873
H	8.50691195	6.33891737	22.04545884
H	8.27065484	6.00215241	20.31630164
C	9.62830411	3.88018721	21.53764061
H	8.84860350	3.53494801	20.84718109
H	9.18297785	3.93169871	22.53897886
H	10.42182709	3.12726769	21.55113008
C	14.01828385	6.70880216	18.12111841
H	14.44083701	6.90217665	19.10947710
C	13.71742129	8.07607621	17.49069680
H	13.25186236	7.97020816	16.50325745
H	14.62985140	8.67341556	17.38711766
H	13.02598660	8.62461918	18.13335727
C	15.04050766	5.93750071	17.27480238
H	15.97894689	6.50153365	17.20954646
H	14.67536514	5.78305747	16.25185074
H	15.25958921	4.95533388	17.70528521
C	12.98862869	11.72866513	17.88106218
H	12.67500095	10.67668769	17.89629121
H	12.08519332	12.34757327	17.85186413
H	13.53814450	11.87483706	16.94124778
C	14.59850876	13.99990208	19.09168256
H	13.72785380	14.66455516	19.13784449
H	15.08179983	14.12751582	18.11337395
H	15.30828727	14.31829777	19.86651013
C	15.65855763	11.15932105	19.20267193
H	16.35357459	11.39272647	20.01979368
H	16.16969168	11.34413197	18.24754917

H	15.42032785	10.09074406	19.26748942
C	13.01005513	14.52563396	22.35215420
H	13.75932736	15.03175036	21.73225973
H	13.08304709	14.92160867	23.37455920
H	12.01785700	14.77391075	21.95813756
C	14.85888090	12.31646918	23.31199341
H	15.05045672	11.23887703	23.38238939
H	14.81992054	12.73107019	24.32876262
H	15.71029857	12.77124522	22.78759765
C	11.81555719	11.99215576	23.40297068
H	10.84646405	12.20898455	22.93293243
H	11.82124241	12.51934389	24.36653106
H	11.86483783	10.92098380	23.64523247
C	6.92908013	8.65123420	18.86917686
H	7.09092994	7.93220197	19.67936485
H	6.59931036	8.10019198	17.97774492
H	6.11623599	9.32358570	19.17438677
C	9.85374378	8.48521420	17.90026523
H	10.70212021	9.05569326	17.49746725
H	9.47788241	7.85491499	17.08506736
H	10.23068615	7.79789202	18.66836886
C	8.05704615	10.73819846	17.02178321
H	7.23290989	11.42345527	17.25379864
H	7.74791657	10.10920208	16.17508908
H	8.91728954	11.34081943	16.70503469
C	8.74869648	11.39964367	20.80141885
C	8.95653423	12.80283192	20.58272064
C	8.69126773	13.72250874	21.60022016
H	8.86434615	14.77923454	21.41028982
C	8.21740061	13.32142514	22.85053757
H	8.02499902	14.05241170	23.63021050
C	8.00648612	11.96312319	23.07982367
H	7.64064800	11.63444020	24.05053742
C	8.26214631	11.00520003	22.09346956
C	8.00879254	9.53942900	22.39190542
H	8.31201211	8.99568345	21.49156960
C	8.84872833	9.03114235	23.57517543
H	9.92423214	9.14126138	23.38866850
H	8.65638267	7.96845913	23.76485505
H	8.62101754	9.58930375	24.49158099
C	6.51595309	9.25891050	22.62599713
H	6.15176670	9.78351053	23.51883801
H	6.33883159	8.18451516	22.76536268
H	5.92472013	9.59749996	21.76935378
C	9.44507162	13.29851272	19.23572053
H	9.71619955	12.40306152	18.66453217
C	8.32177728	14.03110856	18.48237010
H	7.42890953	13.40296448	18.40491236
H	8.64313525	14.30332290	17.46875716
H	8.04199450	14.95176738	19.01124788
C	10.69063765	14.18732920	19.35700875
H	10.47216132	15.10812645	19.91276070
H	11.05526723	14.48016758	18.36398150
H	11.49262075	13.65515258	19.87814568

7⁻

Lowest frequency: 20.0883 cn^{-1}
Second frequency: 22.7984 cm^{-1}

Ca	12.46078550	6.88382643	5.01624822
Si	10.65535978	4.69763344	3.26347227
Si	15.76949356	5.84384586	5.05980898
N	12.27585202	10.35861184	5.14792652
N	10.48892835	5.71877641	4.61865295
N	14.30544469	5.85382054	5.92451534
C	12.63605620	10.46089767	6.52317800
C	12.36606002	9.72862791	2.91014527
C	12.52840139	9.19913660	4.34938346
C	11.78862281	9.98407484	7.54987706
C	12.12370891	10.23918913	8.88653794
H	11.46310117	9.88300633	9.67170860
C	14.03857367	5.36919207	7.21309395
C	8.43675755	7.01851945	5.03435530
C	11.48906071	11.44584963	4.48016682
C	9.29508786	5.92295934	5.33228799
C	7.78169099	5.34826925	7.16949874
H	7.55229736	4.70871140	8.02048291
C	13.51413578	4.06056779	7.41146149
C	10.61149168	9.07183539	7.26292134
H	10.50864650	8.99864171	6.17403283
C	14.91630510	11.32978481	5.80075596
H	14.38174310	11.33216330	4.85105229
C	13.88004894	11.05044891	6.87050381
C	8.95178185	5.10001810	6.44364150
C	13.30359118	4.45004097	9.81853185
C	14.18682902	11.27509868	8.21649186
H	15.13612739	11.73261591	8.47838586
C	14.17432388	6.21090680	8.35451081
C	9.60075408	5.18545651	1.76163296
H	8.54101055	5.23697370	2.04352509
H	9.70249529	4.45390622	0.94826746
H	9.89974034	6.16877567	1.37940474
C	13.69646497	10.29225407	2.35697717
H	14.09414823	11.08565963	2.99411331
H	13.55669100	10.70535454	1.34593595
H	14.44821586	9.49775105	2.30653738
C	8.80851774	7.98222414	3.94524361
H	9.82821695	8.36190887	4.09876944

H	8.11902191	8.83356413	3.92094920
H	8.80363391	7.50719723	2.95850151
C	7.27597365	7.23171644	5.78617951
H	6.64197152	8.08112827	5.53554460
C	11.31766321	10.86406470	3.05608899
H	11.42436297	11.64188381	2.28861667
H	10.31333189	10.43460519	2.95805956
C	12.22994743	12.79386808	4.48799327
H	12.49428422	13.07516632	5.51368583
H	11.58567588	13.57943842	4.07378791
H	13.14461316	12.75708560	3.89165781
C	6.91706839	6.40203520	6.85394691
C	15.50986814	6.89517633	3.50721748
H	14.97819825	6.33636124	2.72664423
H	16.47775672	7.20596922	3.09251352
H	14.93743578	7.80534558	3.73756227
C	12.46764681	4.79720855	2.71117928
H	12.70814200	5.76824760	2.25893720
H	12.64792634	4.03839987	1.93893098
H	13.17479410	4.59602378	3.52766618
C	13.29726429	10.90458052	9.22408037
H	13.53835855	11.09609077	10.26598032
C	13.16495632	3.62831778	8.69405940
H	12.75418201	2.62680268	8.81320419
C	11.89475497	8.64048105	1.93797497
H	12.67657827	7.87824970	1.82467058
H	11.69497331	9.05396200	0.93968553
H	10.98799568	8.14913728	2.30057372
C	10.12696963	11.64542920	5.15966843
H	9.56557205	10.70818802	5.16715187
H	9.53792915	12.39799174	4.61966265
H	10.25600829	11.98695622	6.19283911
C	9.27989846	9.56244419	7.84876066
H	9.32815366	9.60747712	8.94413878
H	8.48153505	8.86563624	7.57309974
H	9.01919977	10.55870699	7.48059014
C	10.91931726	7.66842367	7.82119624
H	11.91180919	7.30448408	7.52932768
H	10.15778219	6.95164832	7.50601531
H	10.94376636	7.68246147	8.91686091
C	10.26482777	2.86025960	3.54156291
H	10.97272114	2.40368133	4.24297617
H	10.31425716	2.30662809	2.59376019
H	9.25353491	2.74261782	3.95156875
C	14.67461609	7.61879896	8.19668915
H	15.74809262	7.64653552	7.97952242
H	14.49092586	8.20793702	9.09916634
H	14.18747658	8.12945956	7.35906469
C	9.89892361	4.02754559	6.89884405
H	10.07171167	3.27252082	6.12783455
H	9.52094659	3.52659215	7.79674804
H	10.88101266	4.45157021	7.13776166
C	13.80514194	5.74035615	9.61936408
H	13.90235492	6.41080928	10.47210020
C	5.64713623	6.63391845	7.63294424
H	4.79930568	6.07602577	7.20899981
H	5.36901422	7.69445998	7.63448028
H	5.75577669	6.31435260	8.67610886
C	16.33819706	4.13166095	4.45584752
H	16.53153319	3.46137543	5.30360706
H	17.26362367	4.21210610	3.86820132
H	15.57017656	3.66907258	3.82325344
C	15.91679142	10.16286277	5.74205312
H	16.46368388	10.06757647	6.68853034
H	16.64272820	10.31305074	4.93321807
H	15.38168077	9.22808995	5.55509940
C	13.29008388	3.16750725	6.22451534
H	12.58684362	3.63244260	5.52427387
H	12.87482272	2.20167692	6.53281506
H	14.21588821	2.99112806	5.66896485
C	15.63055960	12.67578990	5.96512763
H	14.91283094	13.50161831	6.02440257
H	16.29386323	12.85646652	5.11031397
H	16.25018822	12.69926008	6.87020747
C	17.26483882	6.53109799	6.00596085
H	17.16157506	7.60793892	6.17735739
H	18.19643430	6.35715795	5.45008729
H	17.35237727	6.03681749	6.98231991
C	12.93219109	3.95792740	11.19469914
H	12.05609614	3.29917135	11.16047037
H	12.69679945	4.79344985	11.86400242
H	13.74915578	3.38719780	11.66030506

8⁻

Lowest frequency: 17.1935 cm^{-1}
Second frequency: 26.9852 cm^{-1}

Ca	3.87042456	7.08290640	13.08173510
Si	6.74787481	8.32310864	12.18104283
Si	4.51903399	9.96663575	10.97954308
Si	3.05457253	9.11807389	15.64985902
Si	4.26798365	6.42900795	16.45346902
N	5.10125871	8.66657314	11.91569695
N	3.61401030	7.53689915	15.33679527
N	2.04737991	4.22041288	11.92496755
C	6.87227999	6.68610882	13.13598858
H	6.45842565	6.78926669	14.14775807
H	7.92406073	6.39779861	13.25629867

H	6.37499964	5.85298909	12.61844206
C	7.76443103	8.10898336	10.59291703
H	7.29184537	7.36582525	9.93883585
H	8.79037654	7.78211460	10.81046544
H	7.81885484	9.05604305	10.04032988
C	7.67475554	9.57531701	13.26012382
H	7.72467521	10.55984117	12.78103156
H	8.70039567	9.23832709	13.46489092
H	7.15034337	9.69471506	14.21551329
C	4.66864745	9.68365880	9.10660001
H	5.72588347	9.59510972	8.82592386
H	4.23345847	10.52011150	8.54202524
H	4.16602658	8.76044347	8.79939266
C	2.70062777	10.32458987	11.36930276
H	2.07026201	9.42907406	11.30054496
H	2.29533766	11.07007710	10.67193123
H	2.61129048	10.72388808	12.38605546
C	5.42874155	11.60666098	11.29386061
H	5.42155818	11.85112912	12.36298042
H	4.95214366	12.43106320	10.74558228
H	6.47519703	11.54405148	10.96742640
C	1.44110529	9.44710720	14.70061266
H	1.22283414	10.52111180	14.63349754
H	1.46920845	9.05110762	13.67740575
H	0.60758304	8.95550159	15.21616371
C	4.31167276	10.42554361	15.11006906
H	5.20183793	10.37124203	15.75085805
H	3.90929466	11.44643971	15.16091210
H	4.63389402	10.22000450	14.08185259
C	2.63510573	9.48317053	17.46755937
H	1.81726125	8.83885627	17.81302459
H	2.32084477	10.52944417	17.58442563
H	3.50066753	9.31333326	18.12015266
C	5.82063636	7.09526131	17.32662894
H	6.56700997	7.39950473	16.58115393
H	6.27522639	6.34205307	17.98498809
H	5.57792694	7.97829902	17.93213281
C	3.07934281	5.87271084	17.82505956
H	2.68238708	6.72458360	18.38874949
H	3.59014093	5.20062347	18.52863966
H	2.23691633	5.32809358	17.38376325
C	4.77882391	4.83460930	15.57315678
H	3.99547098	4.50116812	14.88225963
H	4.91468240	4.02769617	16.30439851
H	5.71523939	4.93269402	15.01284624
C	2.82054783	5.39533470	11.70299204
C	2.86987434	5.53787828	10.15737457
C	2.14594333	4.27821043	9.60234135

H	1.57028095	4.48938849	8.69282896
H	2.88757753	3.50630877	9.35484166
C	1.25435152	3.75976377	10.74966478
C	2.18616945	6.83298789	9.67918477
H	1.13537753	6.87218072	9.97988450
H	2.23873392	6.92955068	8.58416067
H	2.69090615	7.69813067	10.11654712
C	4.33738216	5.58017621	9.68420232
H	4.83858523	6.46239199	10.10114203
H	4.40588791	5.63483831	8.58678136
H	4.87308932	4.68694776	10.02523213
C	-0.13641061	4.42530109	10.71542320
H	-0.61117857	4.27161757	9.73791974
H	-0.05042346	5.49905090	10.89706112
H	-0.78936130	3.99871065	11.48180214
C	1.06412596	2.24169873	10.72034026
H	2.01860672	1.71890116	10.62683877
H	0.43645121	1.96388879	9.86517559
H	0.56994388	1.89503597	11.63488216
C	2.11954551	3.51051961	13.15479755
C	1.24596179	3.81402957	14.22303761
C	1.36214816	3.10151567	15.42542701
H	0.69620562	3.34052414	16.24951109
C	2.32310257	2.10705142	15.58102845
H	2.40490877	1.56919159	16.52112496
C	3.19699140	1.82273843	14.53247316
H	3.96524175	1.06661969	14.66527553
C	3.11964009	2.51892945	13.32118204
C	0.19056460	4.89423598	14.09438155
H	0.33740726	5.35914443	13.11484170
C	-1.22410381	4.29204120	14.16385379
H	-1.35111667	3.47433315	13.44618630
H	-1.98056561	5.05862447	13.95522603
H	-1.42074120	3.88774352	15.16517028
C	0.35292523	5.98795289	15.15991211
H	0.17464036	5.59125903	16.16765684
H	-0.37372917	6.79074378	14.98552540
H	1.35761369	6.42394949	15.14365727
C	4.13868317	2.24550043	12.22914917
H	3.77233328	2.73830567	11.32387401
C	4.31268778	0.74717684	11.94099422
H	4.96685004	0.60218568	11.07247528
H	3.35070292	0.26471197	11.73519904
H	4.77297811	0.22703350	12.78996550
C	5.48583084	2.89936766	12.57233548
H	5.88518921	2.51551989	13.51855446
H	5.34629733	3.97824113	12.66908745
H	6.22126672	2.71254691	11.77943704

5. References

(1) Sheldrick, G. M. *Program for Area Detector Absorption Correction*, Institute for Inorganic Chemistry, University of Göttingen: Göttingen, Germany, 1996.

(2) Sheldrick, G. M. A Short History of SHELX. *Acta Cryst. A* **2008**, *64* (1), 112–122. https://doi.org/10.1107/S0108767307043930.

(3) Sheldrick, G. M. SHELXT – Integrated Space-Group and Crystal-Structure Determination. *Acta Cryst. A* **2015**, *71* (1), 3–8. https://doi.org/10.1107/S2053273314026370.

(4) Dolomanov, O. V.; Bourhis, L. J.; Gildea, R. J.; Howard, J. A. K.; Puschmann, H. OLEX2: A Complete Structure Solution, Refinement and Analysis Program. *J. Appl. Cryst.* **2009**, *42* (2), 339–341. https://doi.org/10.1107/S0021889808042726.

(5) Vargas, W.; Englich, U.; Ruhlandt-senge, K. A Novel Group of Alkaline Earth Metal Amides : Syntheses and Characterization of $M[N(2,6\text{-}^{i}Pr_2C_6H_3)(SiMe_3)]_2(THF)_2$ (M = Mg, Ca, Sr, Ba) and the Linear , Two-Coordinate $Mg[N(2,6\text{-}^{i}Pr_2C_6H_3)(SiMe_3)]_2$. *Inorg. Chem.* **2002**, *41* (21), 5602–5608.

(6) Westerhausen, M.; Schwarz, W. Molekül- und Kristallstrukturen des dimeren Calcium-bis[bis(trimethylsilyl)amids] und des Calcium-bis[bis(trimethylsilyl)-amids]·DME. *Z. Für Anorg. Allg. Chem.* **1991**, *604* (1), 127–140. https://doi.org/10.1002/zaac.19916040116.

(7) Fischer, C. A.; Rösch, A.; Elsen, H.; Ballmann, G.; Wiesinger, M.; Langer, J.; Färber, C.; Harder, S. Lewis Acidic Alkaline Earth Metal Complexes with a Perfluorinated Diphenylamide Ligand. *Dalton Trans.* **2019**, *48* (20), 6757–6766. https://doi.org/10.1039/C9DT01272A.
